# Supplementary figures and images for: RIPK1 ablation in T cells results in spontaneous enteropathy and TNF-driven villus atrophy
Source: EMBO Rep. 2025 Apr 30;26(10):2654–82. doi: 10.1038/s44319-025-00441-5 (PMC12117051; doi:10.1038/s44319-025-00441-5)

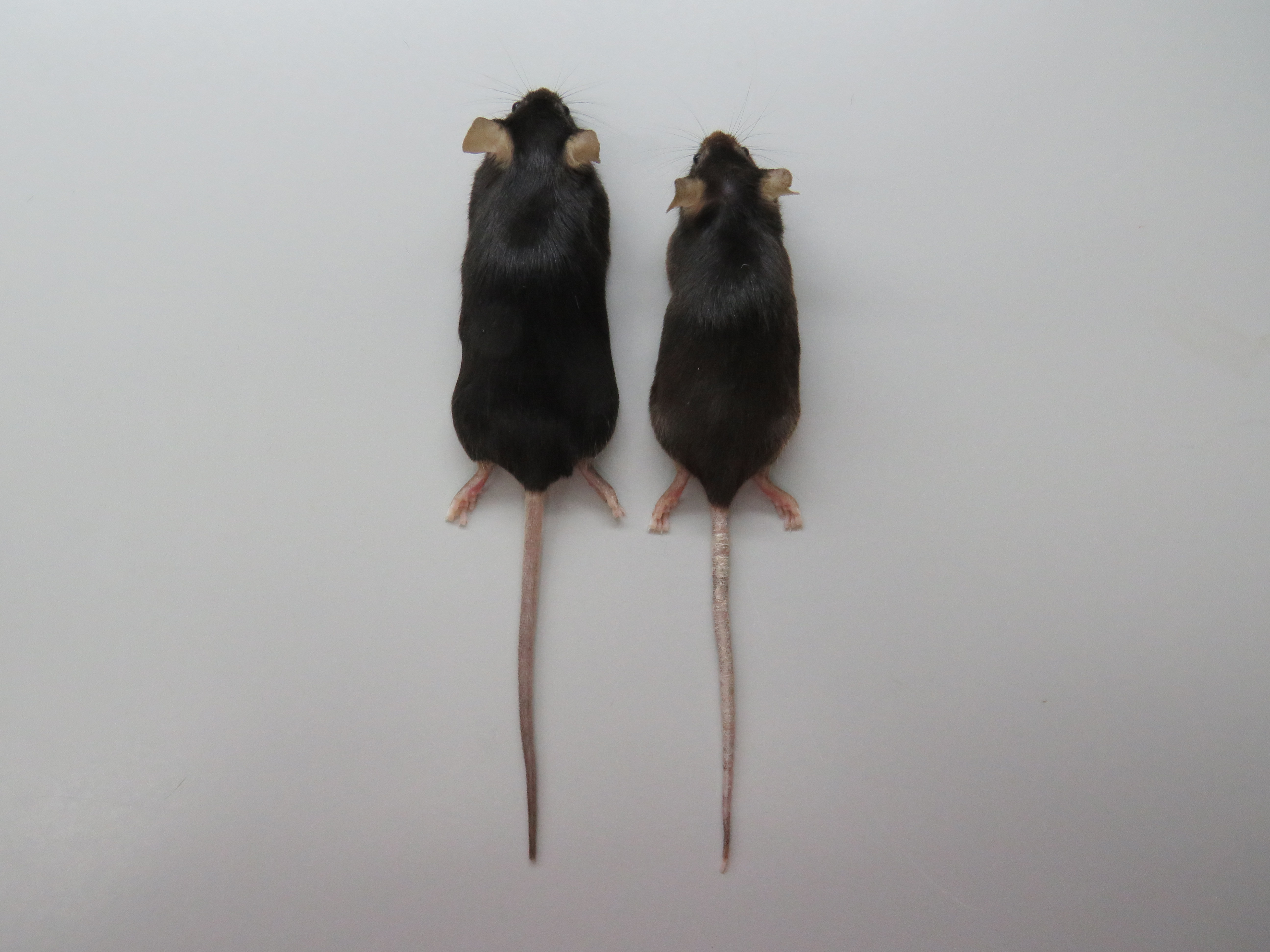

Supplement: Supplementary file 2 — Source data Fig. 1 [file 44319_2025_441_MOESM2_ESM.zip › Figure 1/1A/IMG_6591.JPG]

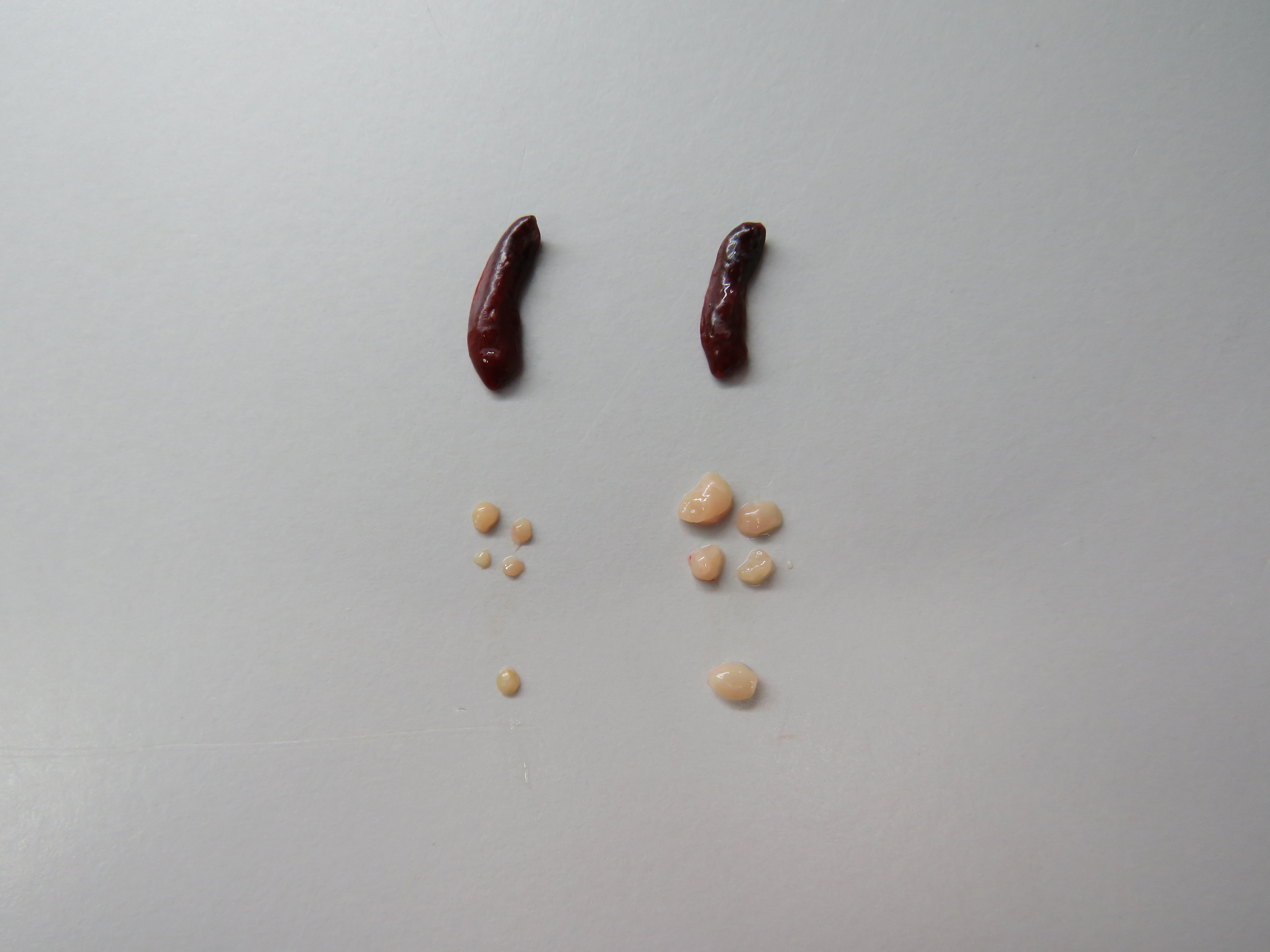

Supplement: Supplementary file 2 — Source data Fig. 1 [file 44319_2025_441_MOESM2_ESM.zip › Figure 1/1E/IMG_7683.JPG]

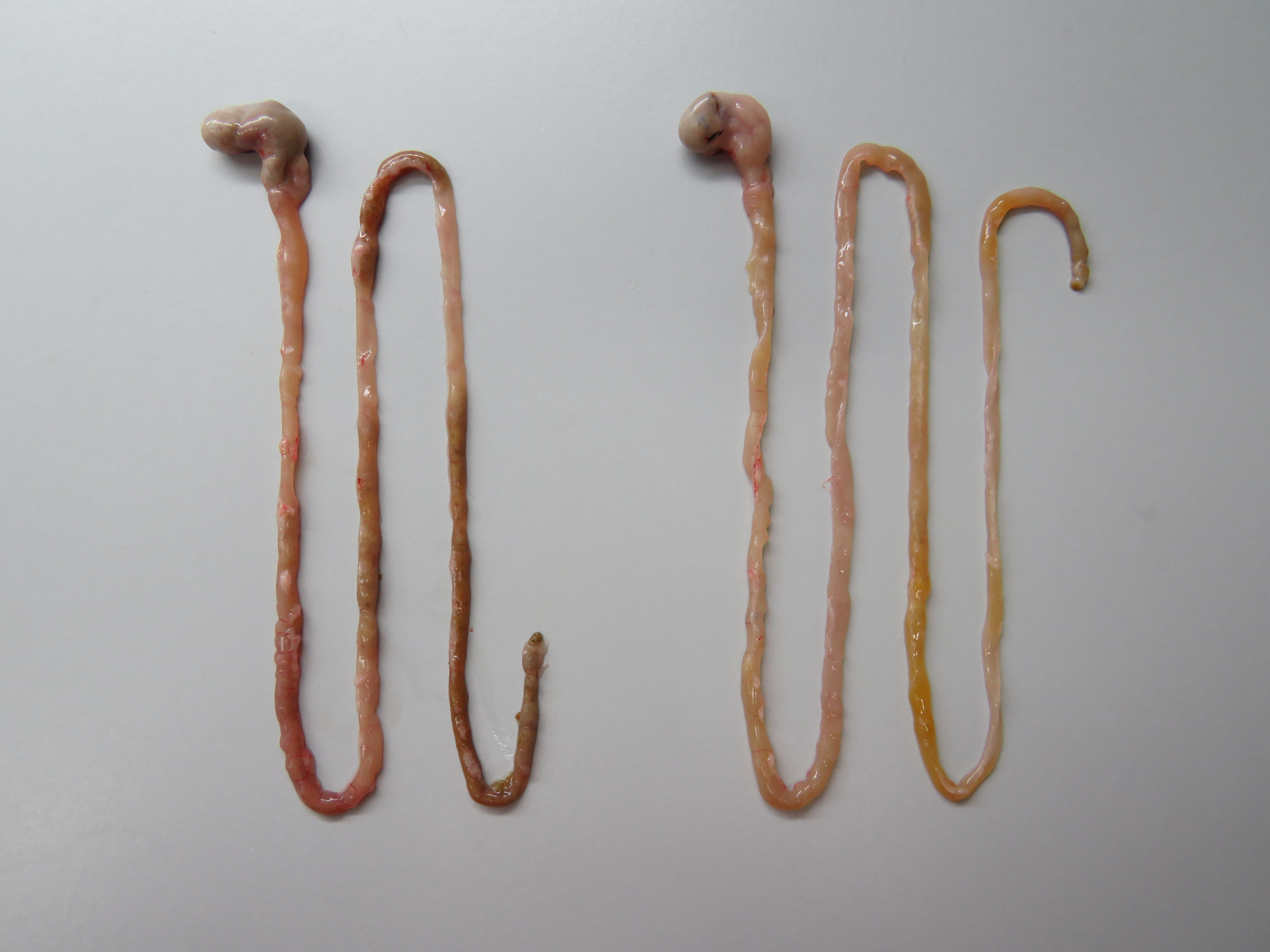

Supplement: Supplementary file 2 — Source data Fig. 1 [file 44319_2025_441_MOESM2_ESM.zip › Figure 1/1E/IMG_7690.JPG]

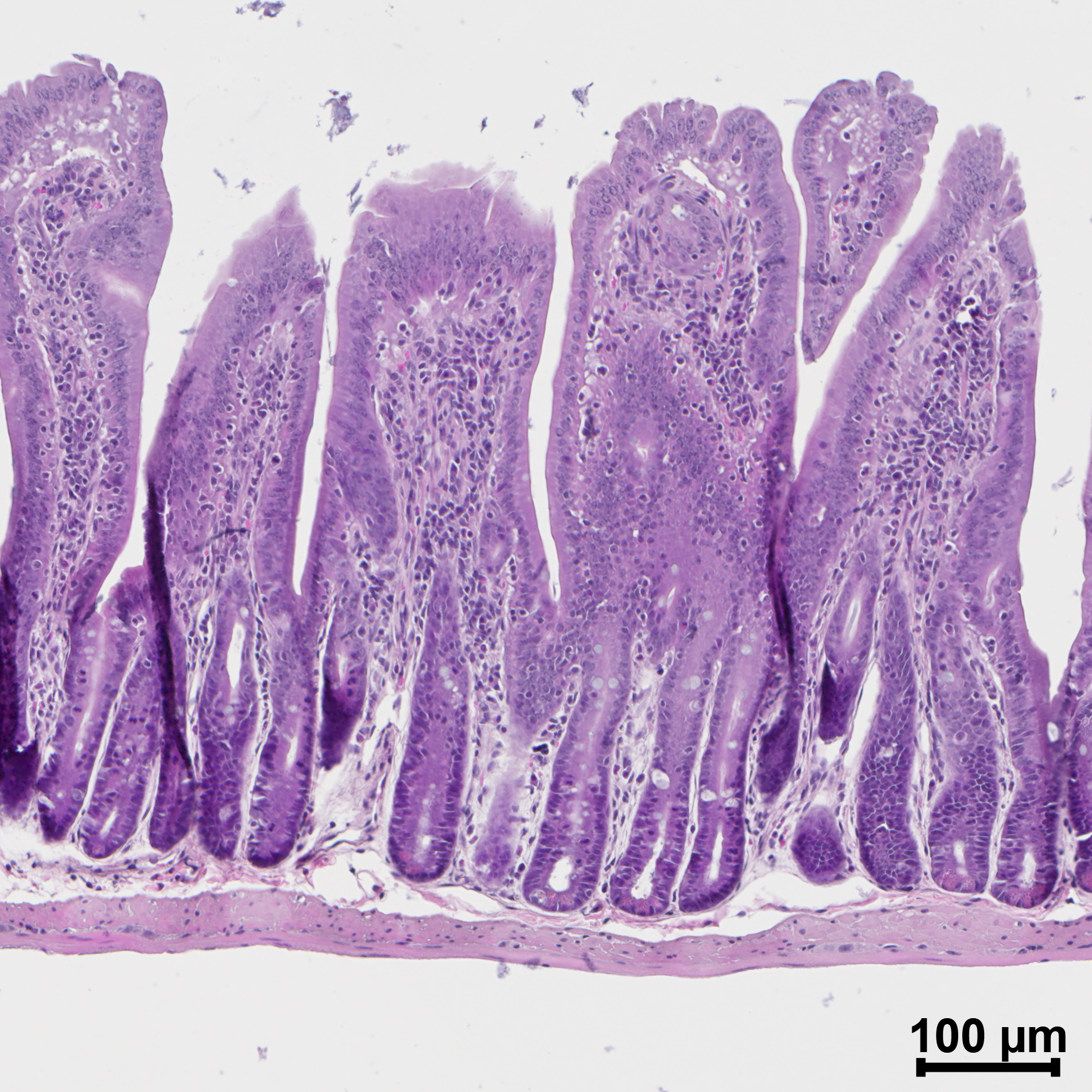

Supplement: Supplementary file 3 — Source data Fig. 2 [file 44319_2025_441_MOESM3_ESM.zip › Figure 2/2A/Duodenum_H&E_R64_mouse 8_KO.bmp]

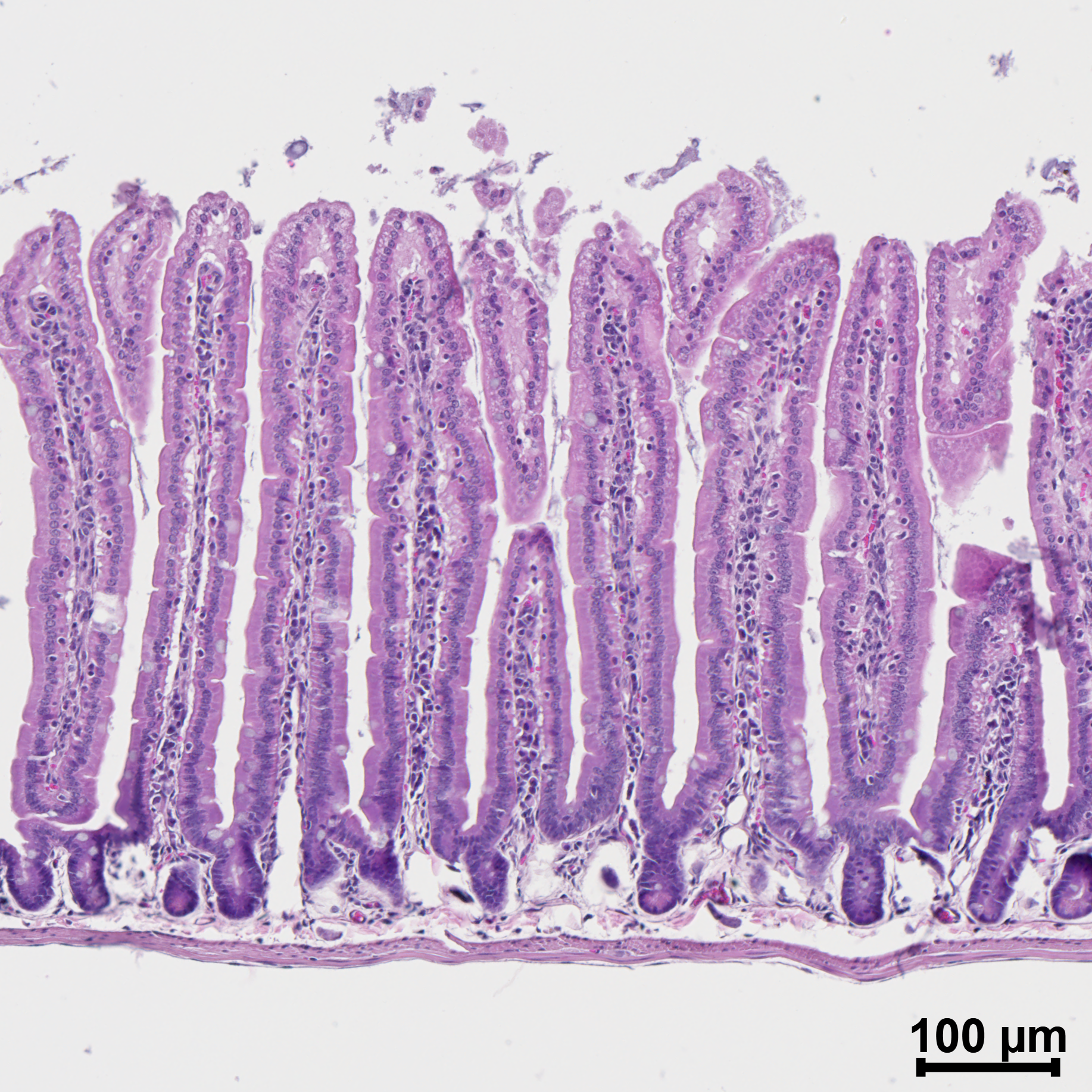

Supplement: Supplementary file 3 — Source data Fig. 2 [file 44319_2025_441_MOESM3_ESM.zip › Figure 2/2A/Duodenum_H&E_R64_mouse 9_WT.bmp]

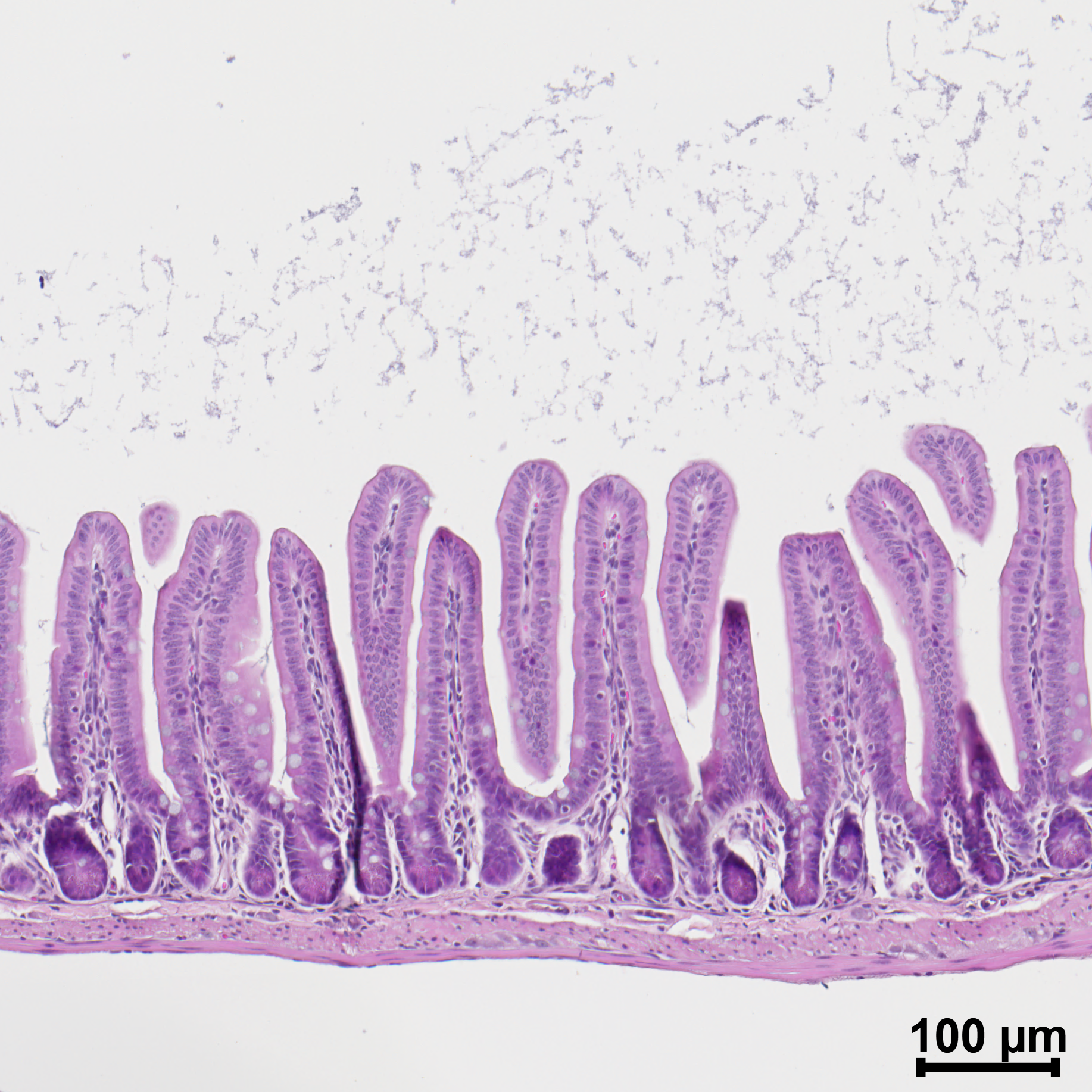

Supplement: Supplementary file 3 — Source data Fig. 2 [file 44319_2025_441_MOESM3_ESM.zip › Figure 2/2A/Ileum_H&E_R64_mouse 4_WT.bmp]

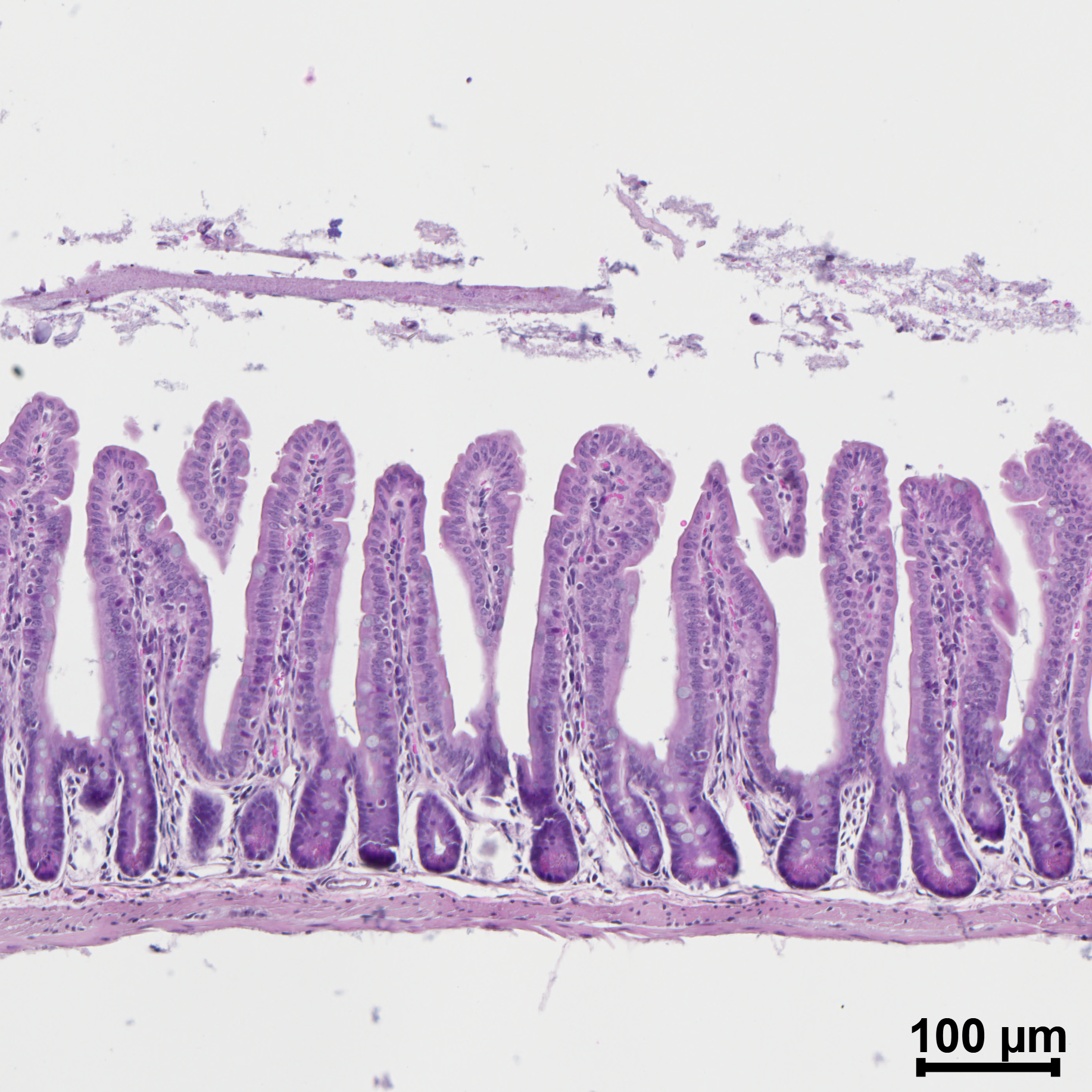

Supplement: Supplementary file 3 — Source data Fig. 2 [file 44319_2025_441_MOESM3_ESM.zip › Figure 2/2A/Ileum_H&E_R64_mouse 8_KO.bmp]

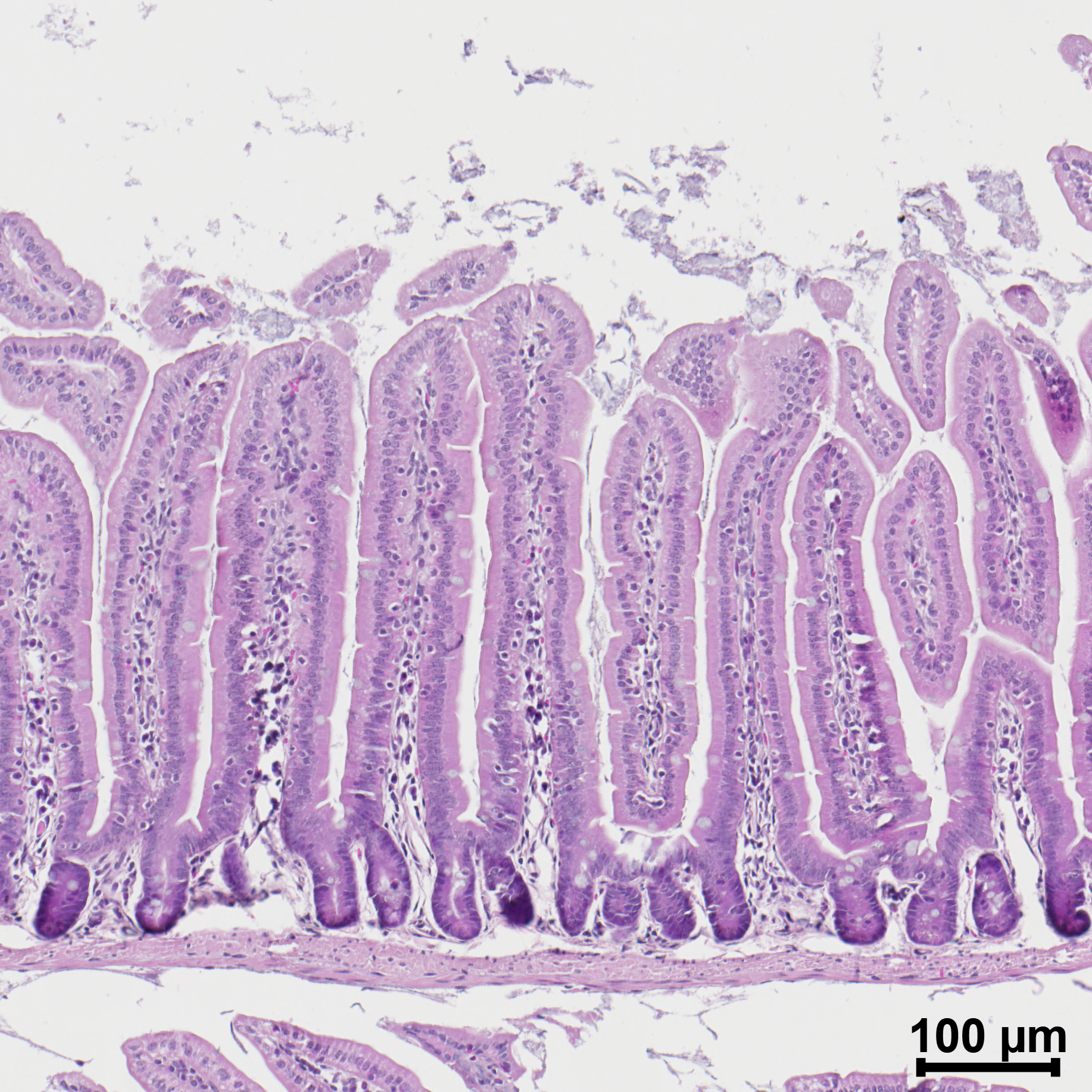

Supplement: Supplementary file 3 — Source data Fig. 2 [file 44319_2025_441_MOESM3_ESM.zip › Figure 2/2A/Jejunum_H&E_R64_mouse 4_WT.bmp]

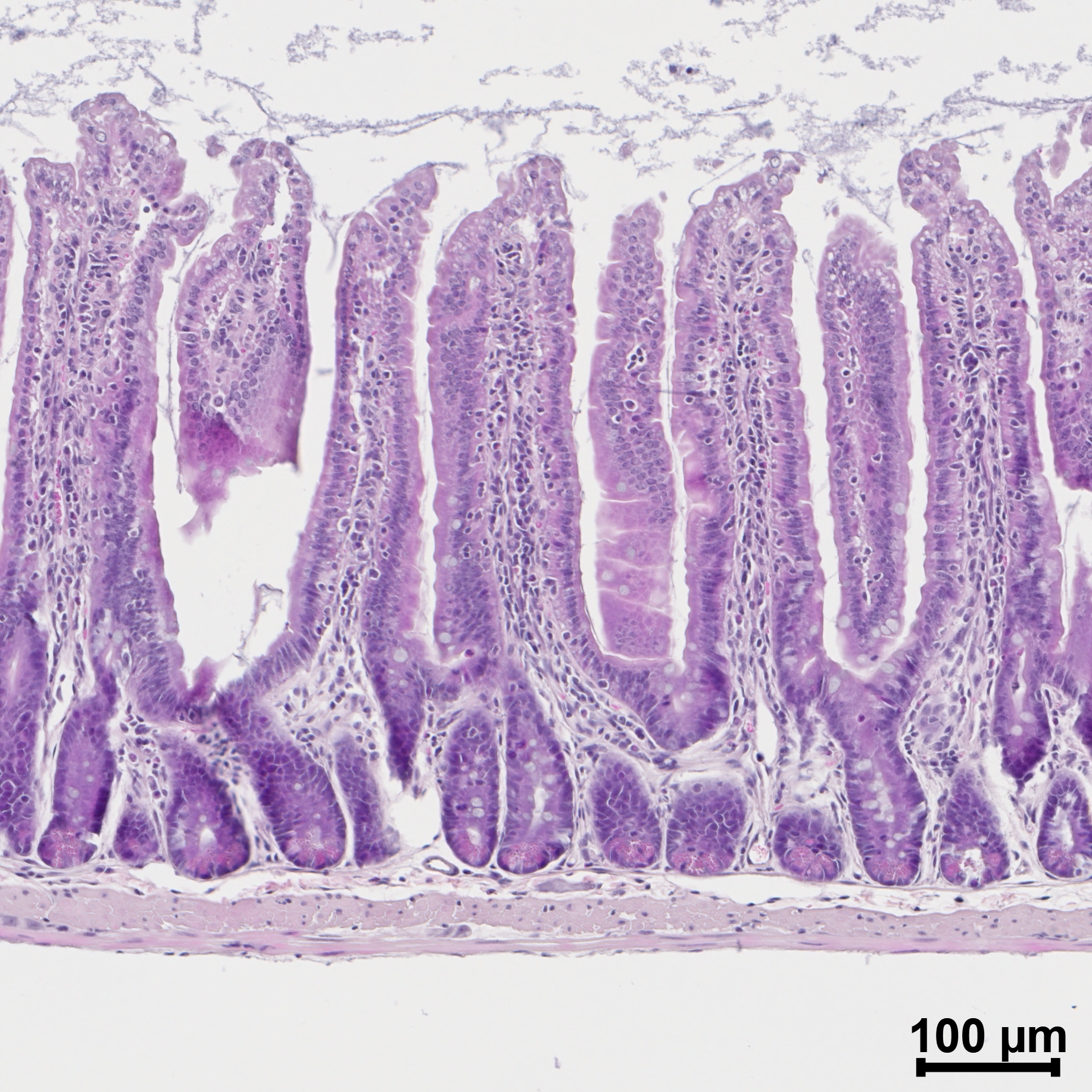

Supplement: Supplementary file 3 — Source data Fig. 2 [file 44319_2025_441_MOESM3_ESM.zip › Figure 2/2A/Jejunum_H&E_R64_mouse 8_KO.bmp]

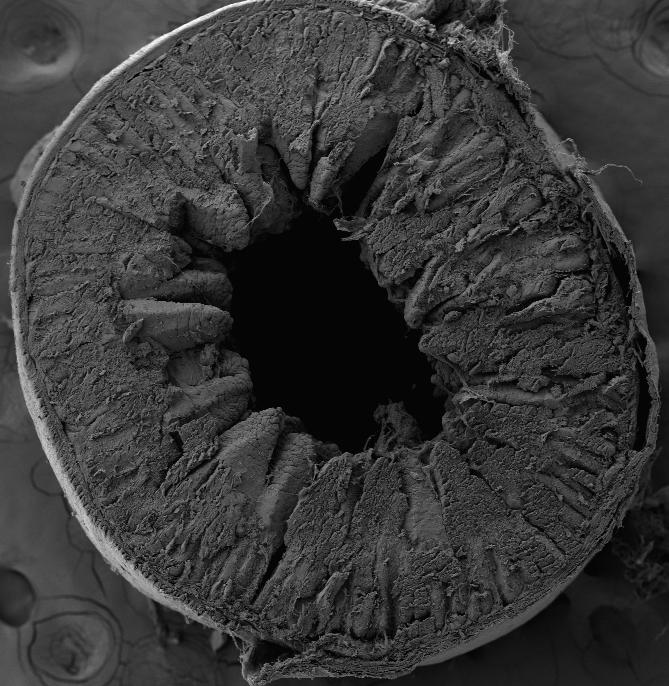

Supplement: Supplementary file 3 — Source data Fig. 2 [file 44319_2025_441_MOESM3_ESM.zip › Figure 2/2D/Duodenum_SEM 40x_R98_mouse 4_KO.png]

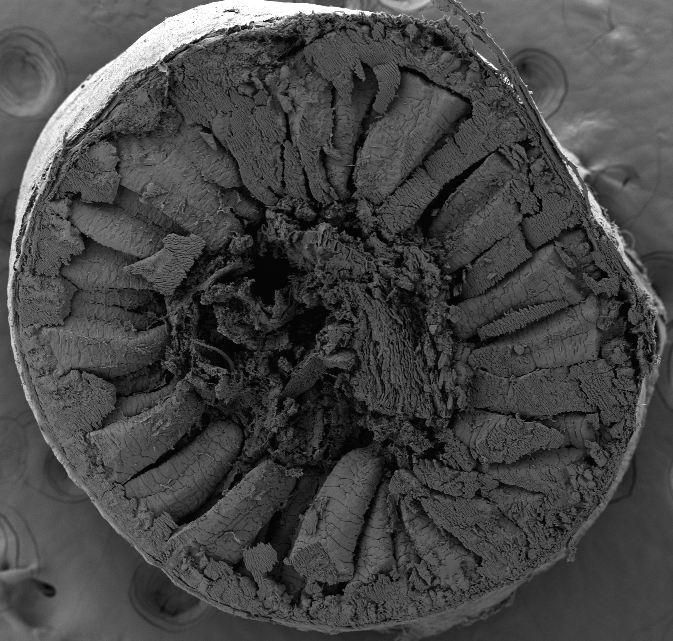

Supplement: Supplementary file 3 — Source data Fig. 2 [file 44319_2025_441_MOESM3_ESM.zip › Figure 2/2D/Duodenum_SEM 40x_R98_mouse 6_WT.png]

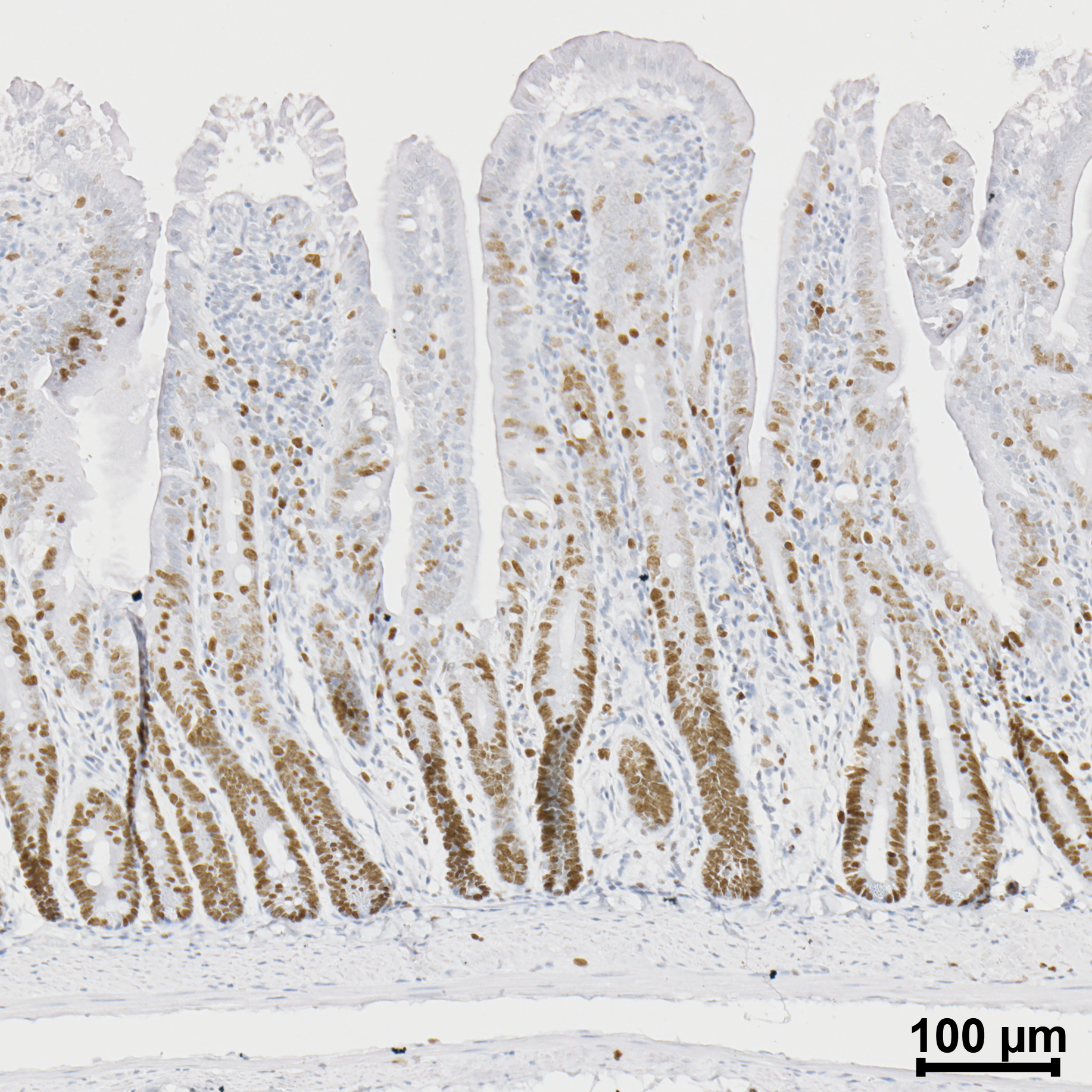

Supplement: Supplementary file 3 — Source data Fig. 2 [file 44319_2025_441_MOESM3_ESM.zip › Figure 2/2E/Duodenum_Ki-67_R64_mouse 2_KO.bmp]

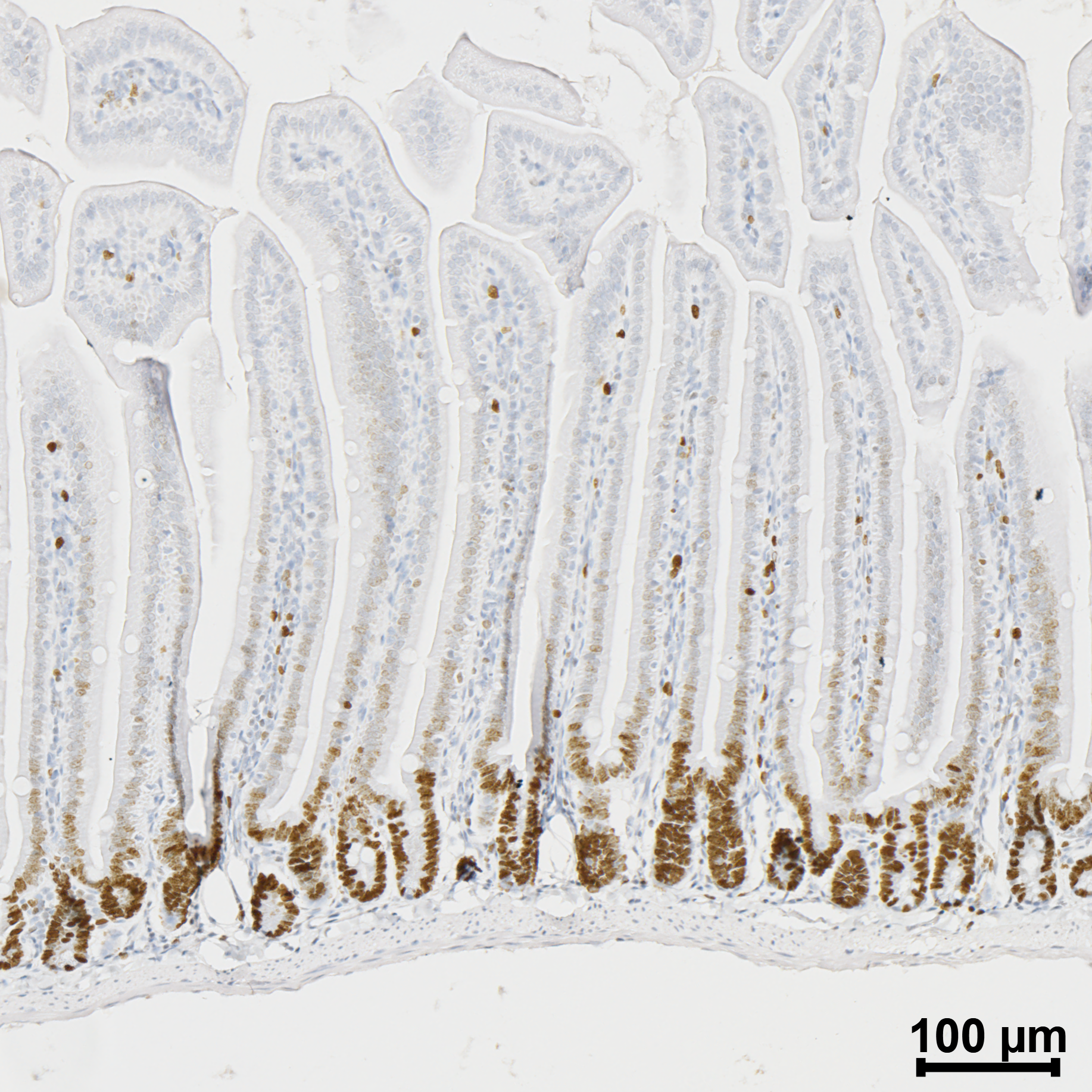

Supplement: Supplementary file 3 — Source data Fig. 2 [file 44319_2025_441_MOESM3_ESM.zip › Figure 2/2E/Duodenum_Ki-67_R64_mouse 4_WT.bmp]

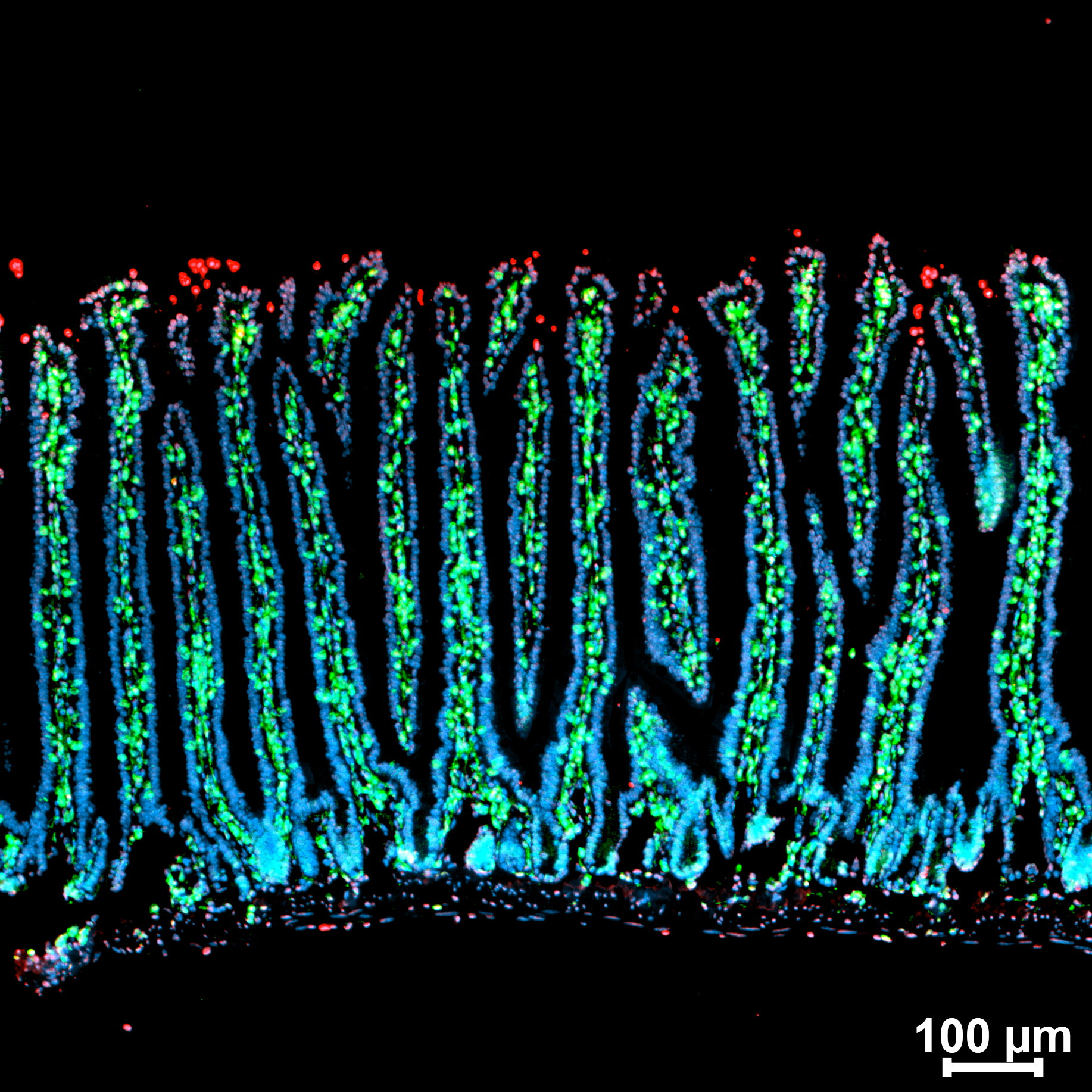

Supplement: Supplementary file 3 — Source data Fig. 2 [file 44319_2025_441_MOESM3_ESM.zip › Figure 2/2G/Duodenum_CD45-TUNEL_R64_mouse 3_WT.bmp]

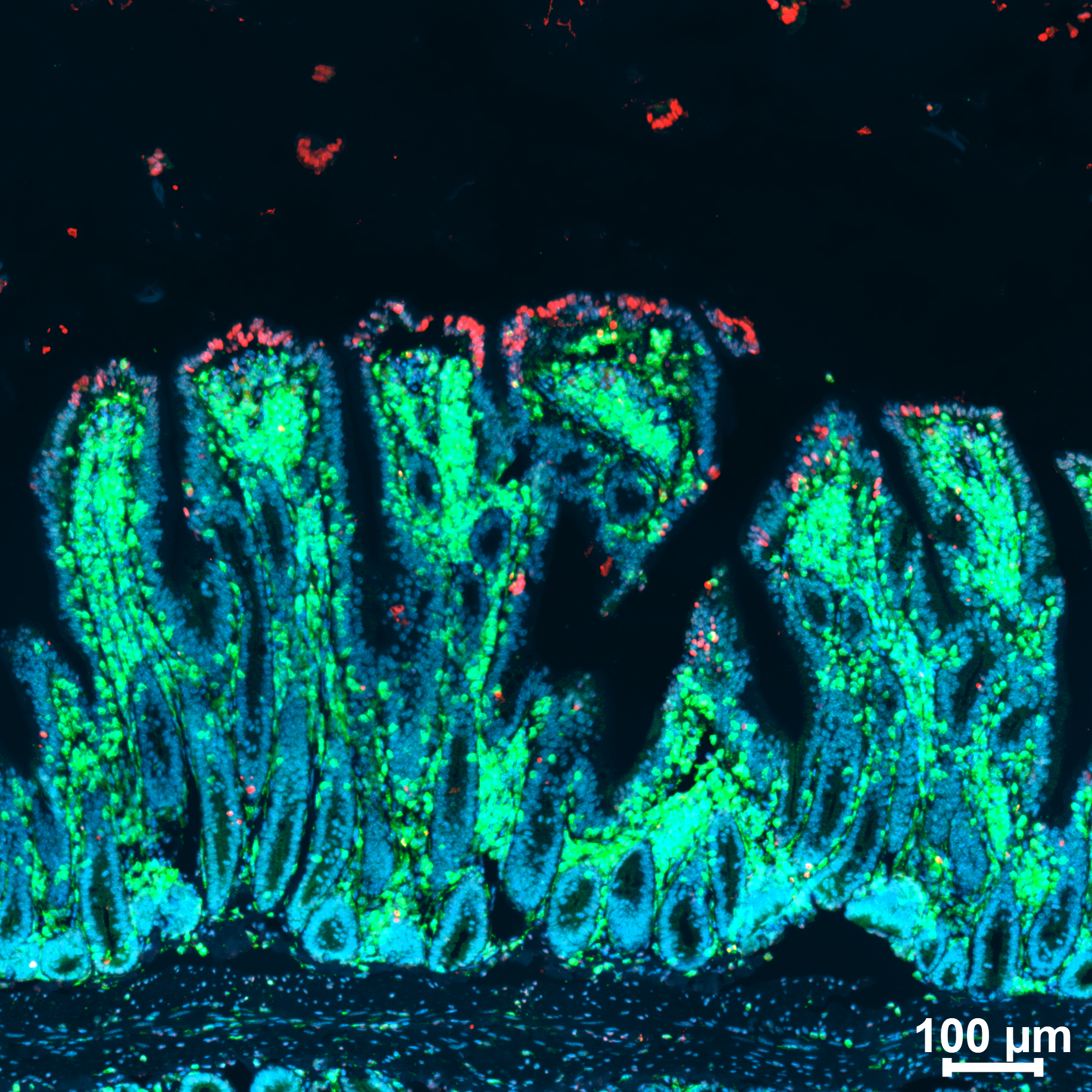

Supplement: Supplementary file 3 — Source data Fig. 2 [file 44319_2025_441_MOESM3_ESM.zip › Figure 2/2G/Duodenum_CD45-TUNEL_R64_mouse 8_KO.bmp]

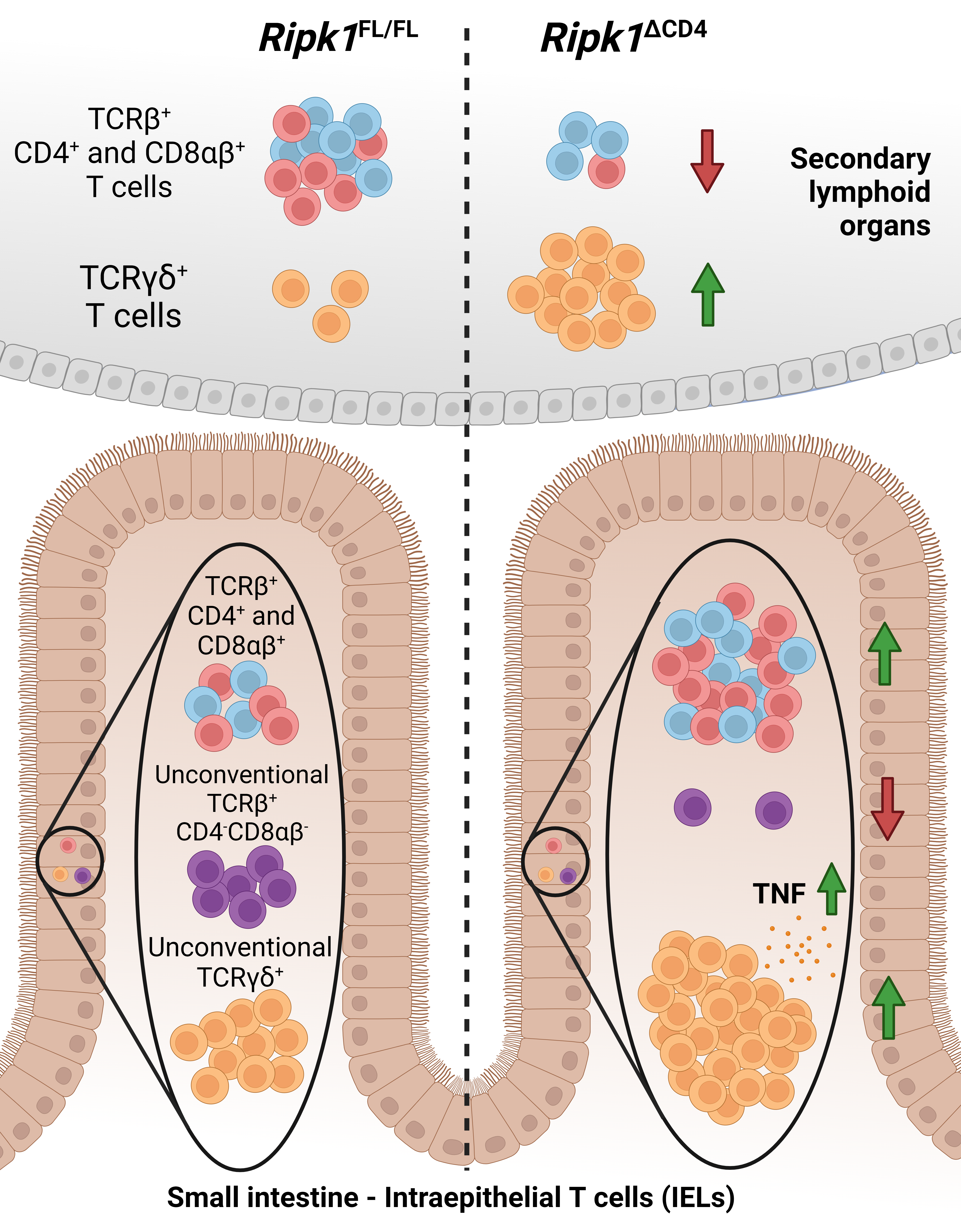

Supplement: Supplementary file 4 — Source data Fig. 3 [file 44319_2025_441_MOESM4_ESM.zip › Figure 3/3M/Schematic overview IELs (2).png]

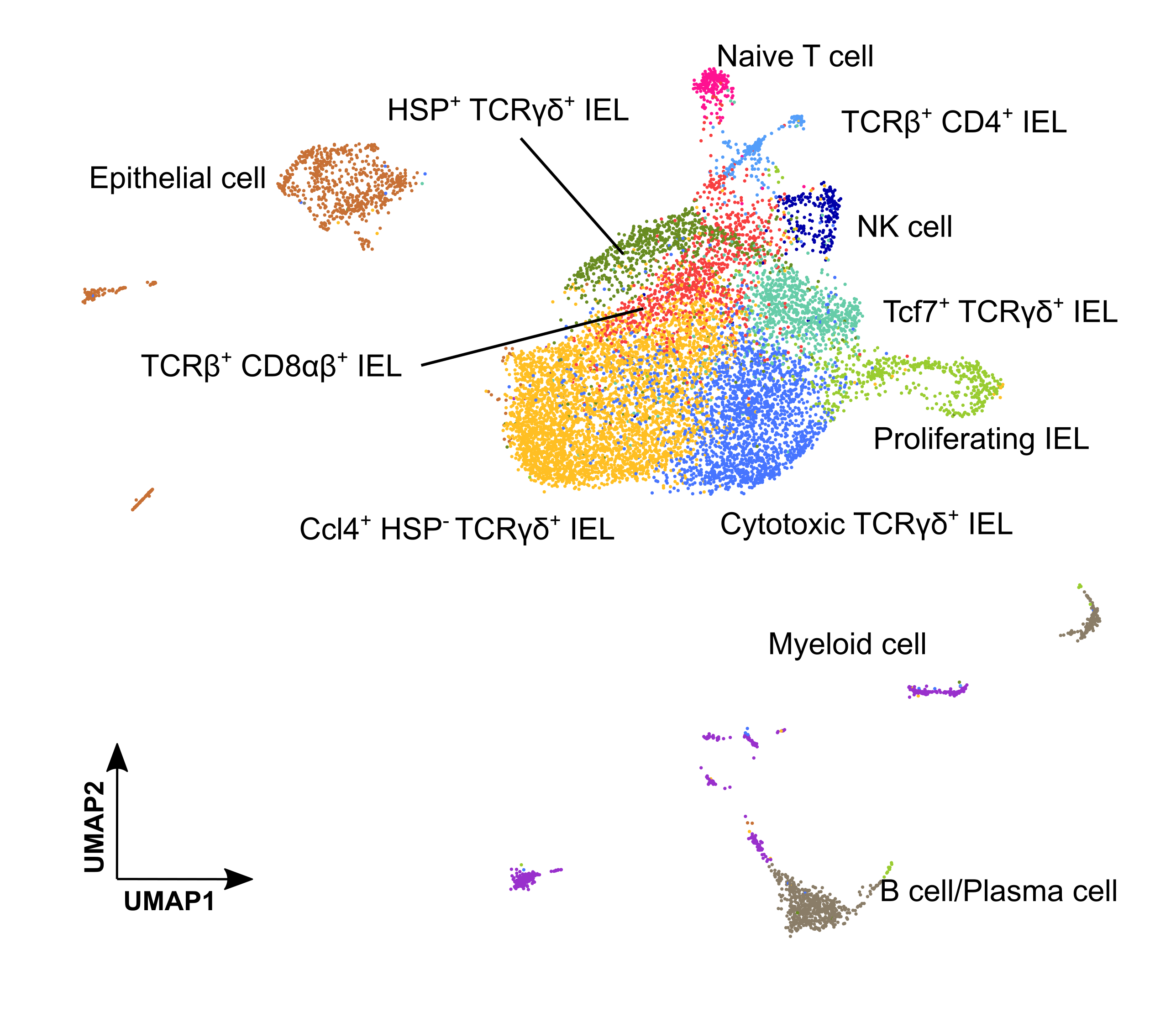

Supplement: Supplementary file 5 — Source data Fig. 4 [file 44319_2025_441_MOESM5_ESM.zip › Figure 4/4A/UMAP.png]

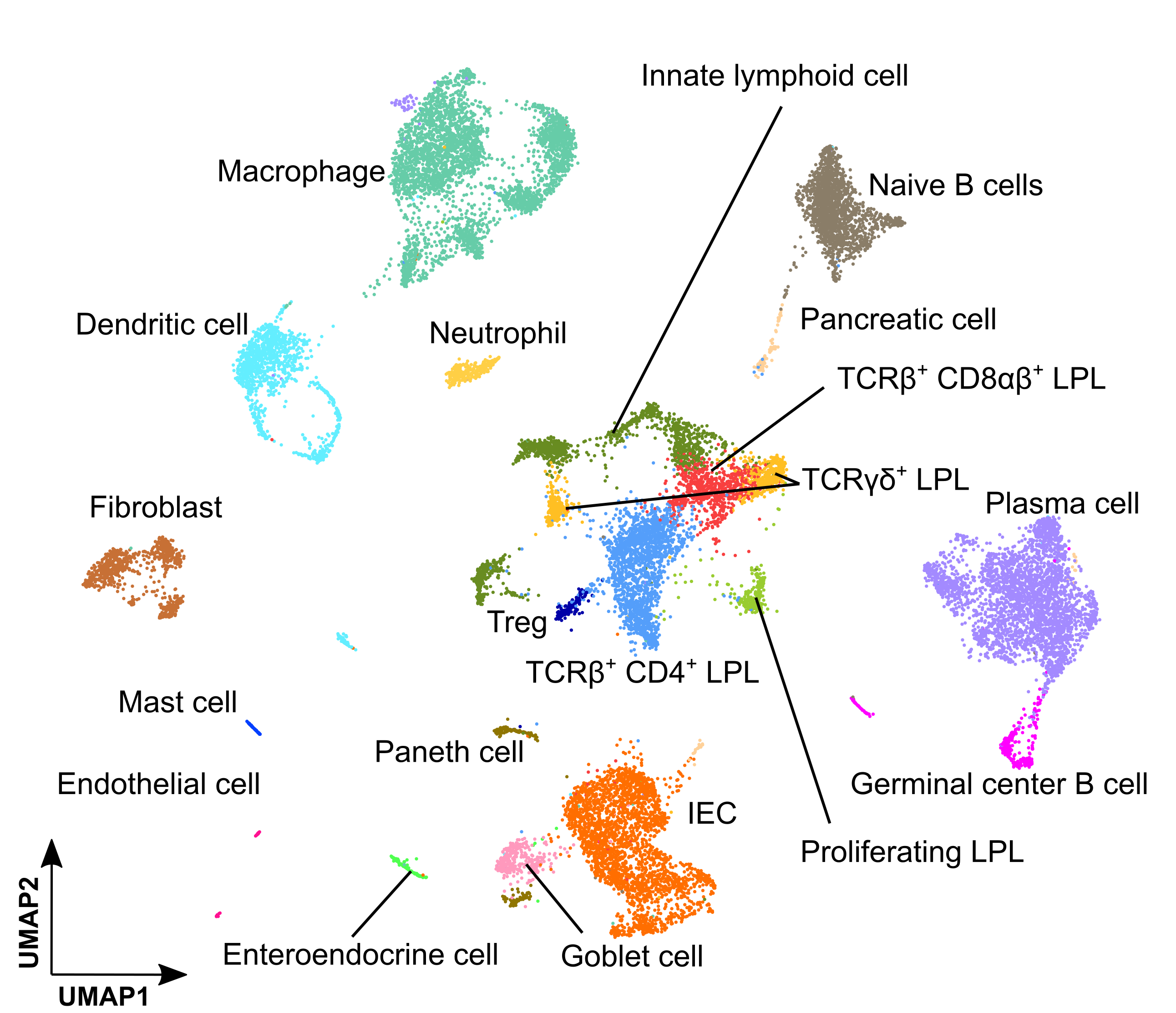

Supplement: Supplementary file 6 — Source data Fig. 5 [file 44319_2025_441_MOESM6_ESM.zip › Figure 5/5A/UMAP.png]

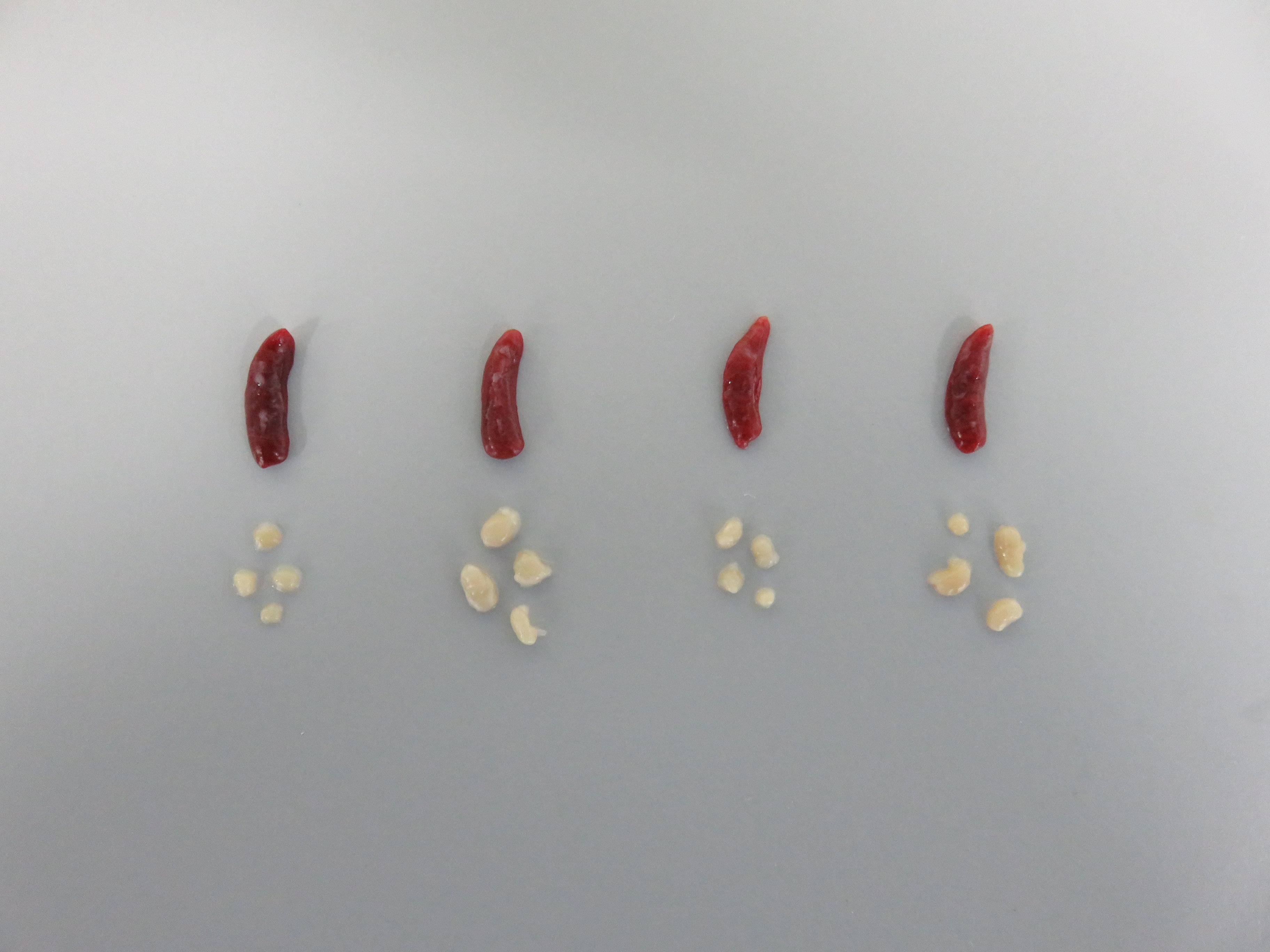

Supplement: Supplementary file 7 — Source data Fig. 6 [file 44319_2025_441_MOESM7_ESM.zip › Figure 6/6D/IMG_3592.JPG]

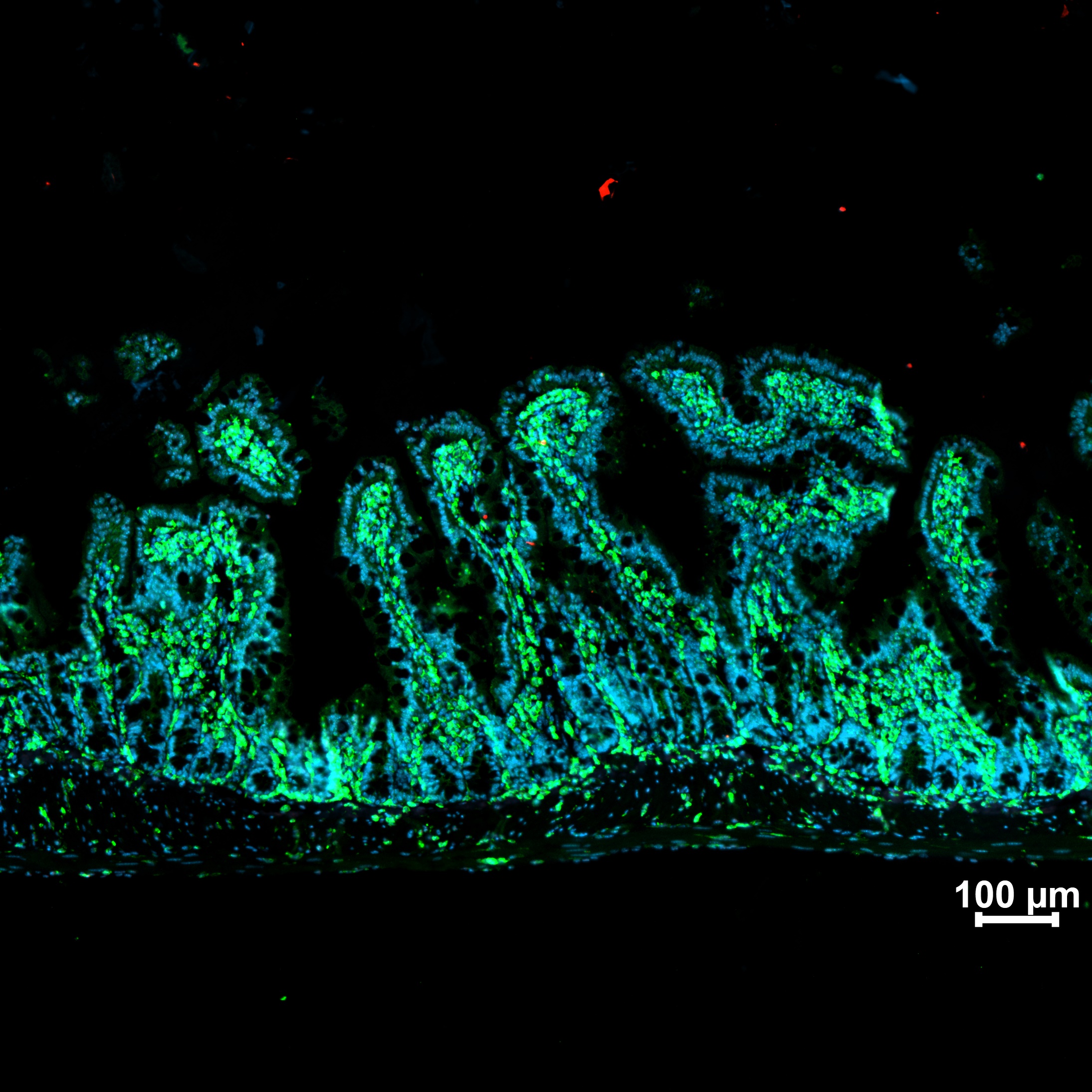

Supplement: Supplementary file 7 — Source data Fig. 6 [file 44319_2025_441_MOESM7_ESM.zip › Figure 6/6E/Duodenum_KO_ABX_CD45_R88_mouse 9.jpg]

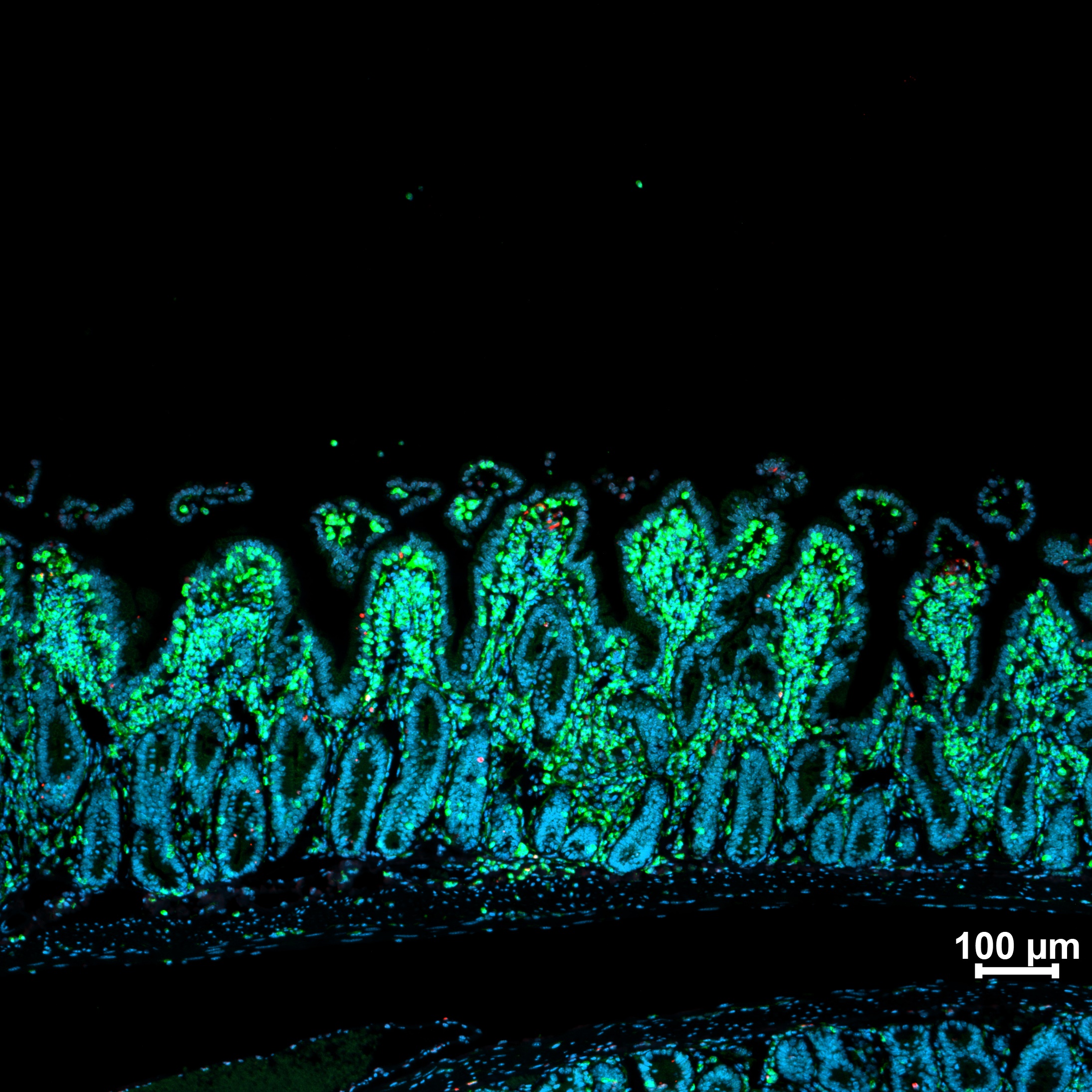

Supplement: Supplementary file 7 — Source data Fig. 6 [file 44319_2025_441_MOESM7_ESM.zip › Figure 6/6E/Duodenum_KO_Control_CD45_R88_mouse 11.jpg]

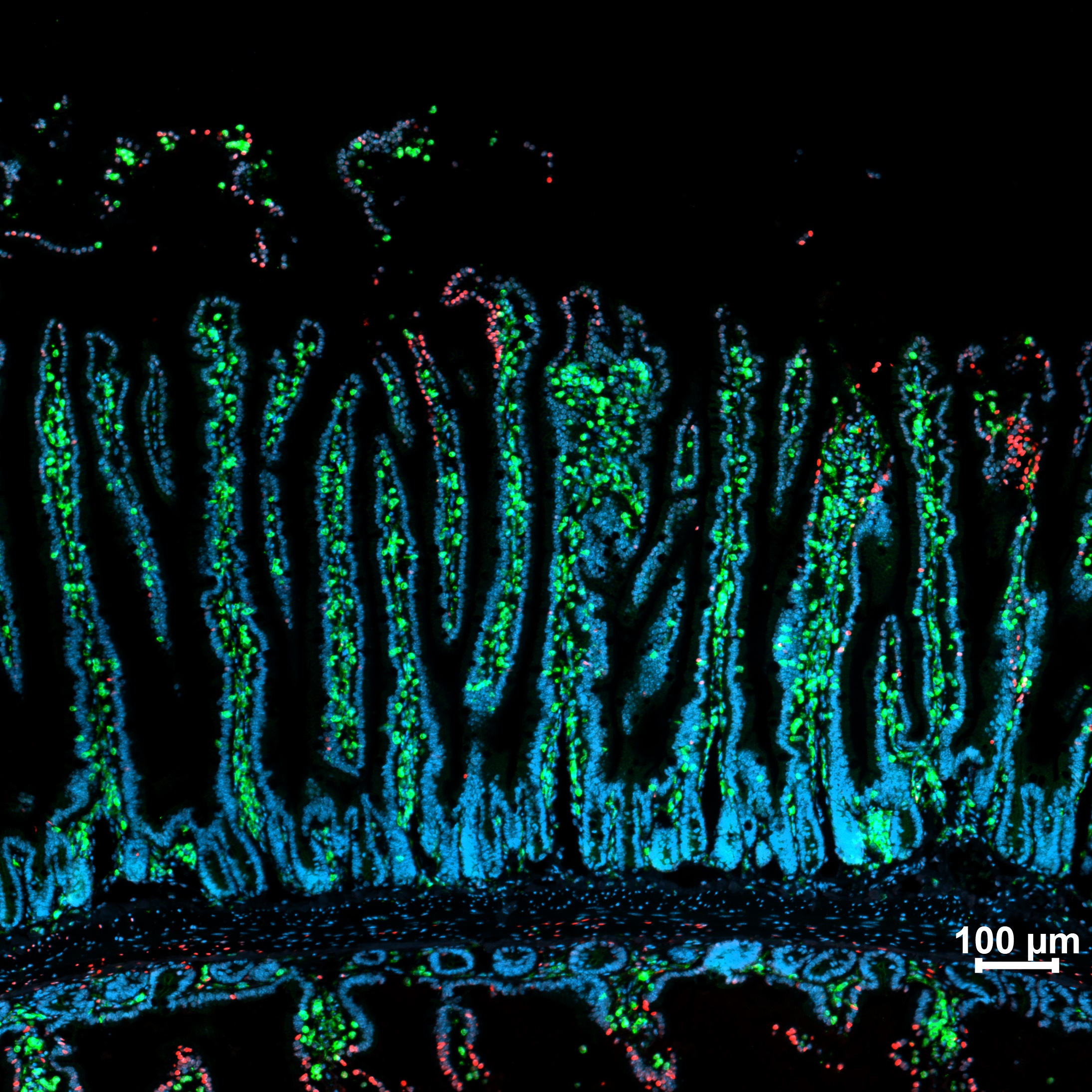

Supplement: Supplementary file 7 — Source data Fig. 6 [file 44319_2025_441_MOESM7_ESM.zip › Figure 6/6E/Duodenum_WT_ABX_CD45_R88_mouse 2.jpg]

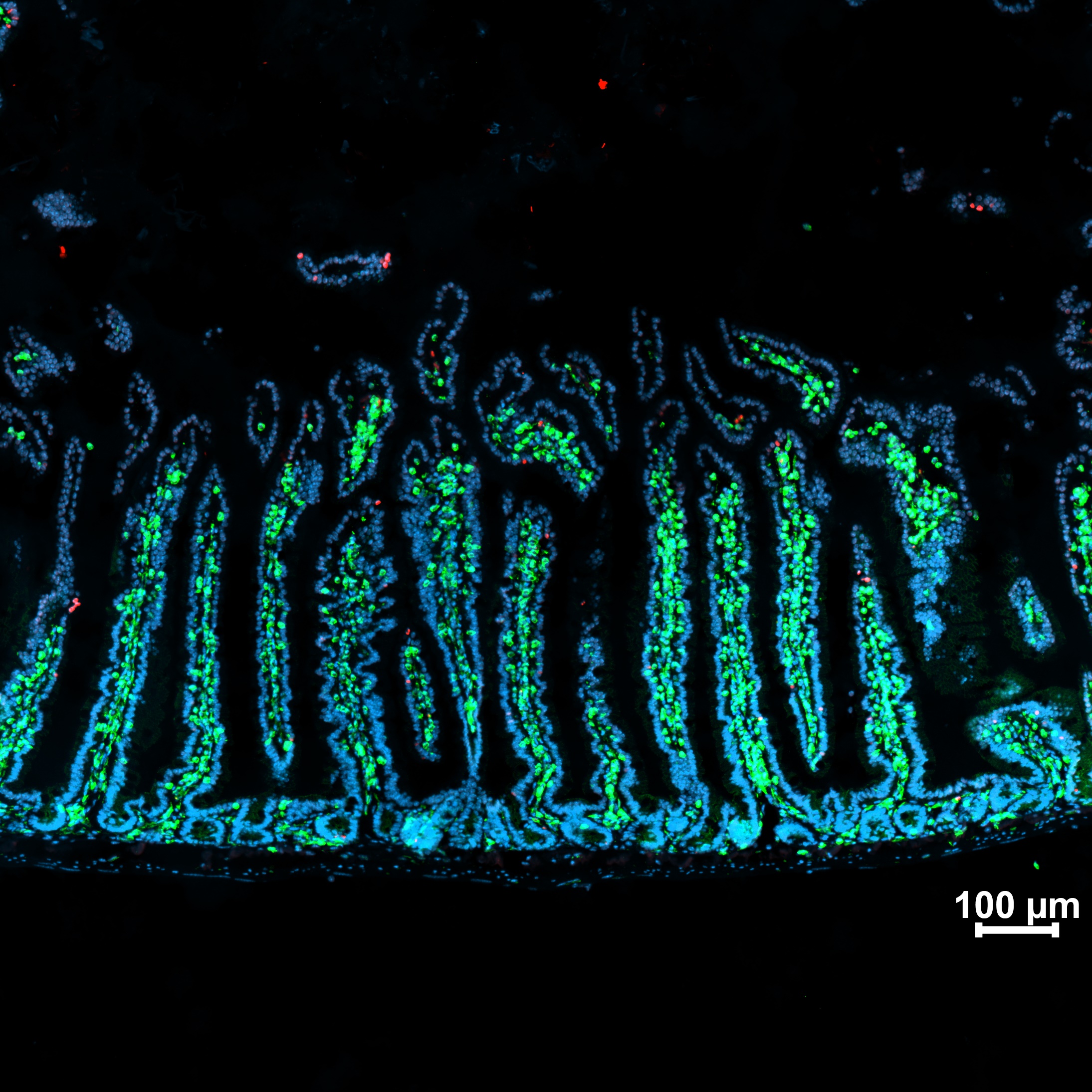

Supplement: Supplementary file 7 — Source data Fig. 6 [file 44319_2025_441_MOESM7_ESM.zip › Figure 6/6E/Duodenum_WT_Control_CD45_R88_mouse 4.jpg]

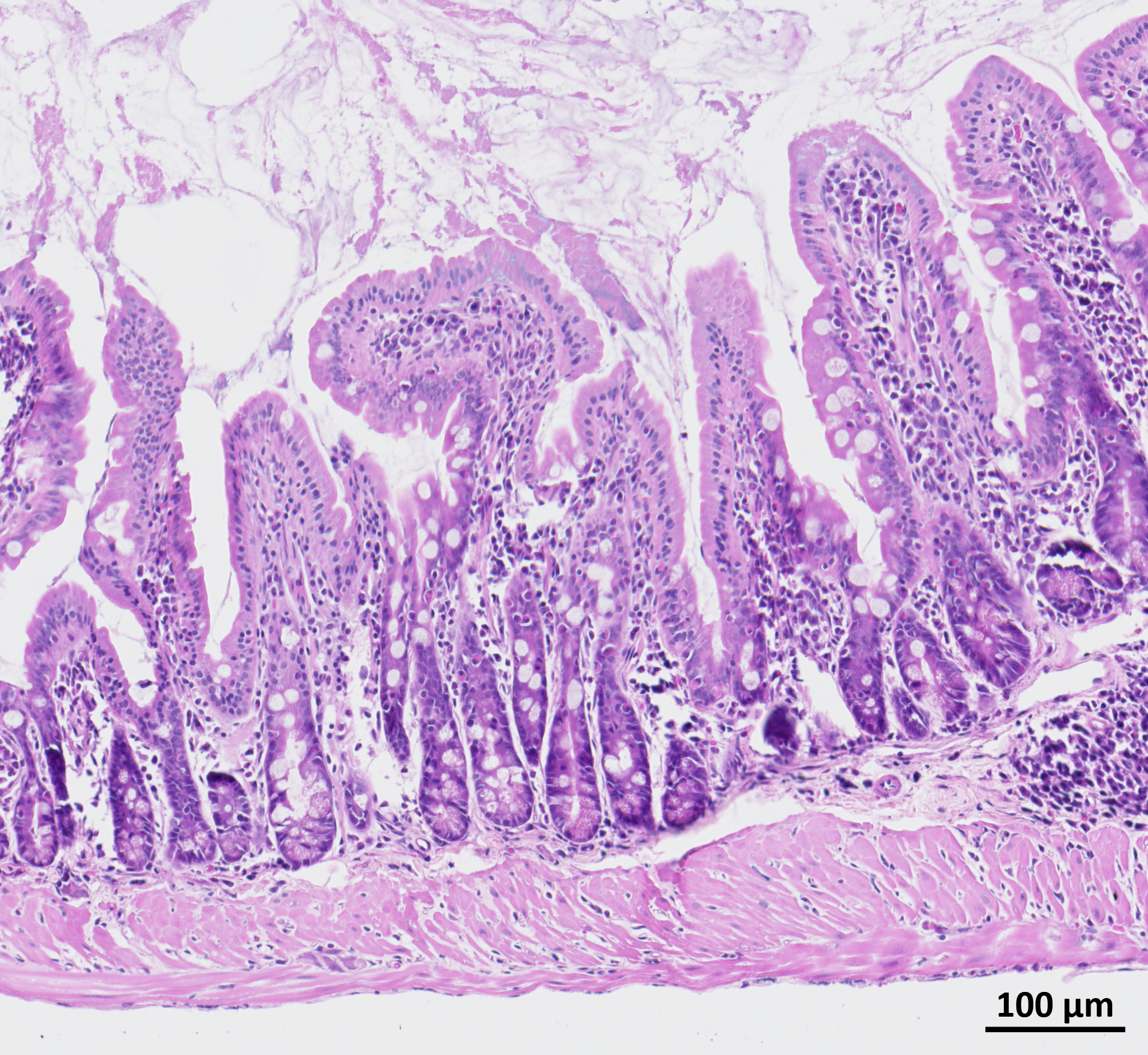

Supplement: Supplementary file 7 — Source data Fig. 6 [file 44319_2025_441_MOESM7_ESM.zip › Figure 6/6F/Duodenum_KO_ABX_H&E_R88_mouse 9.tif]

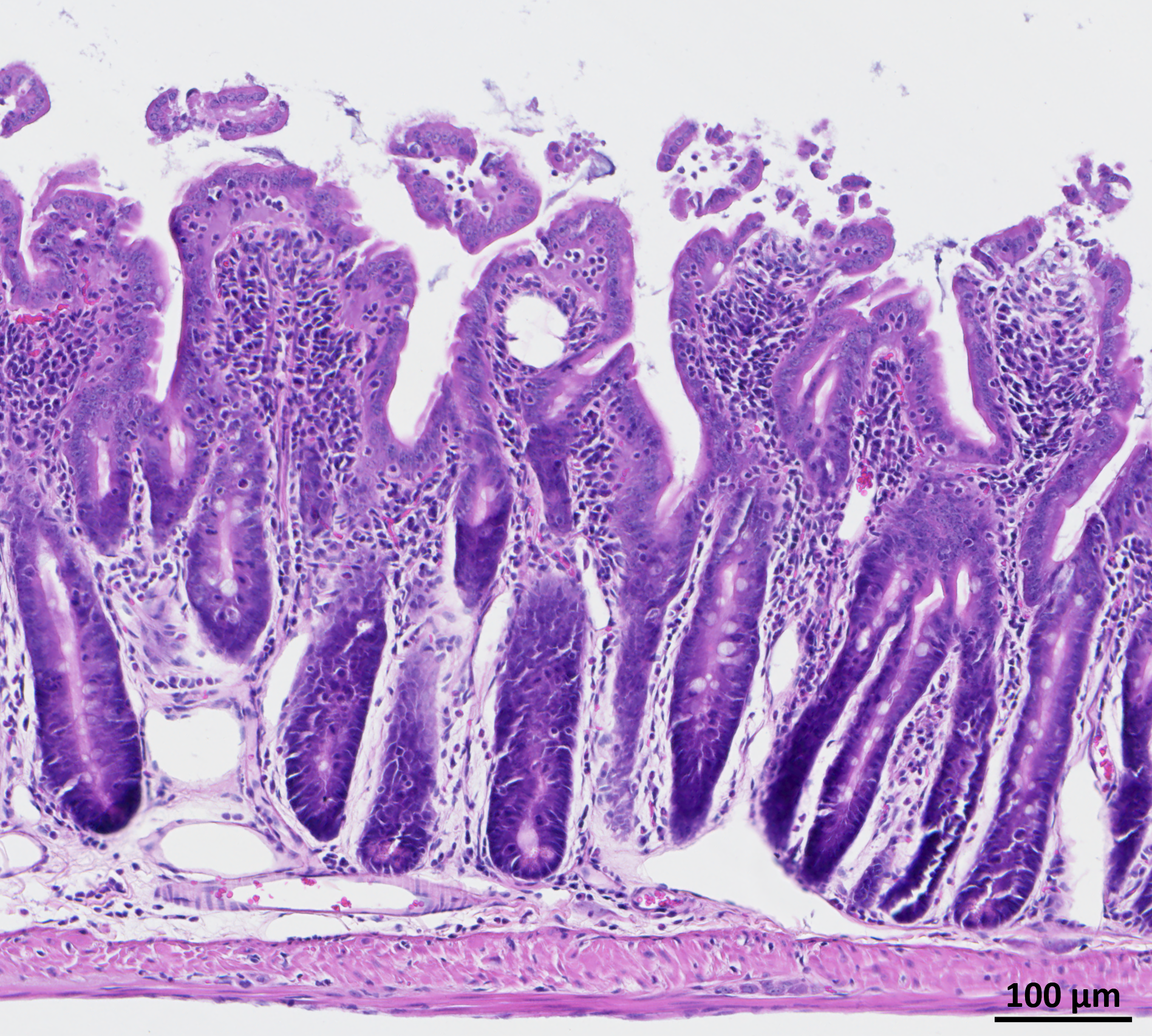

Supplement: Supplementary file 7 — Source data Fig. 6 [file 44319_2025_441_MOESM7_ESM.zip › Figure 6/6F/Duodenum_KO_Control_H&E_R88_mouse 11.tif]

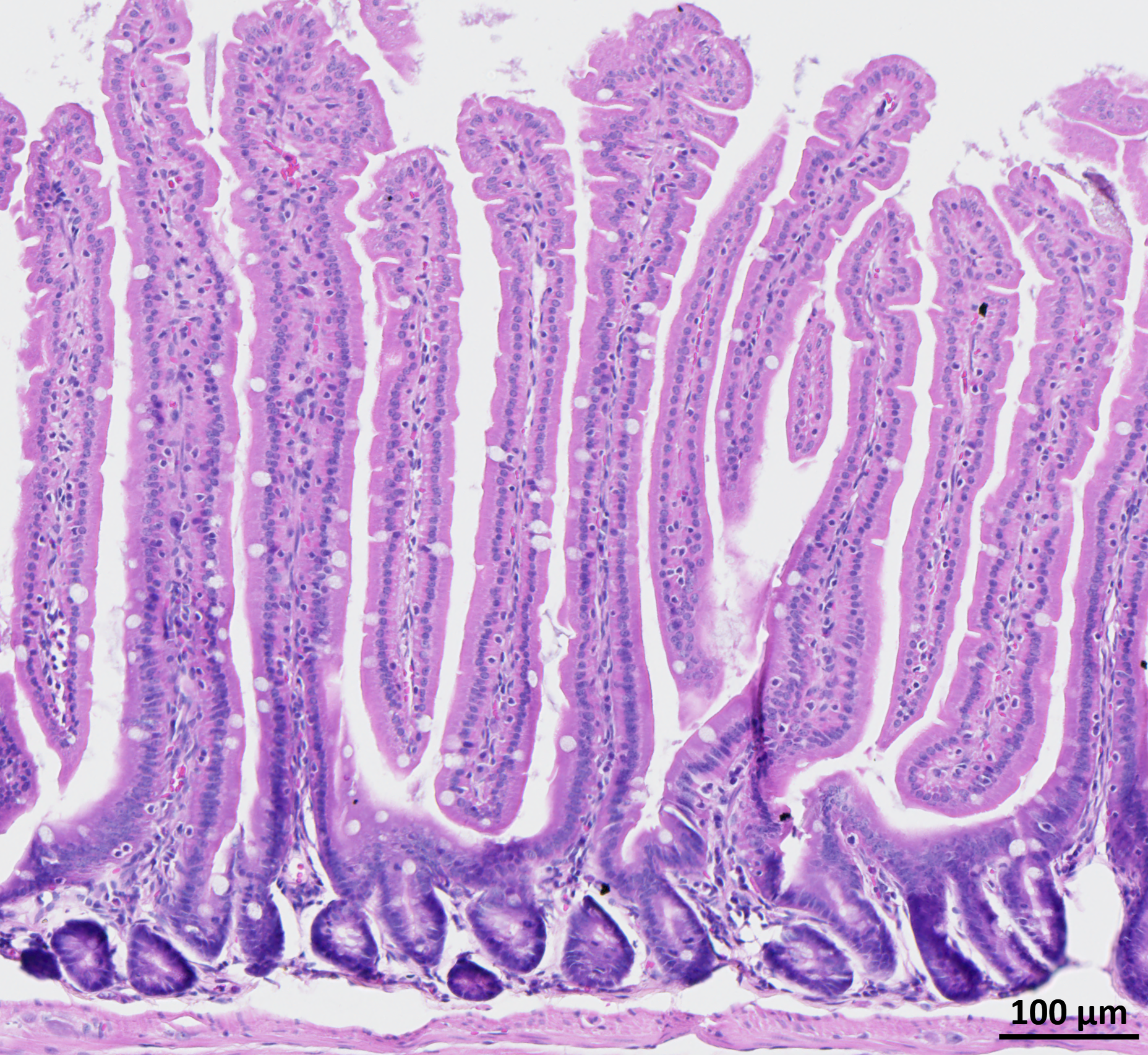

Supplement: Supplementary file 7 — Source data Fig. 6 [file 44319_2025_441_MOESM7_ESM.zip › Figure 6/6F/Duodenum_WT_ABX_H&E_R88_mouse 6.tif]

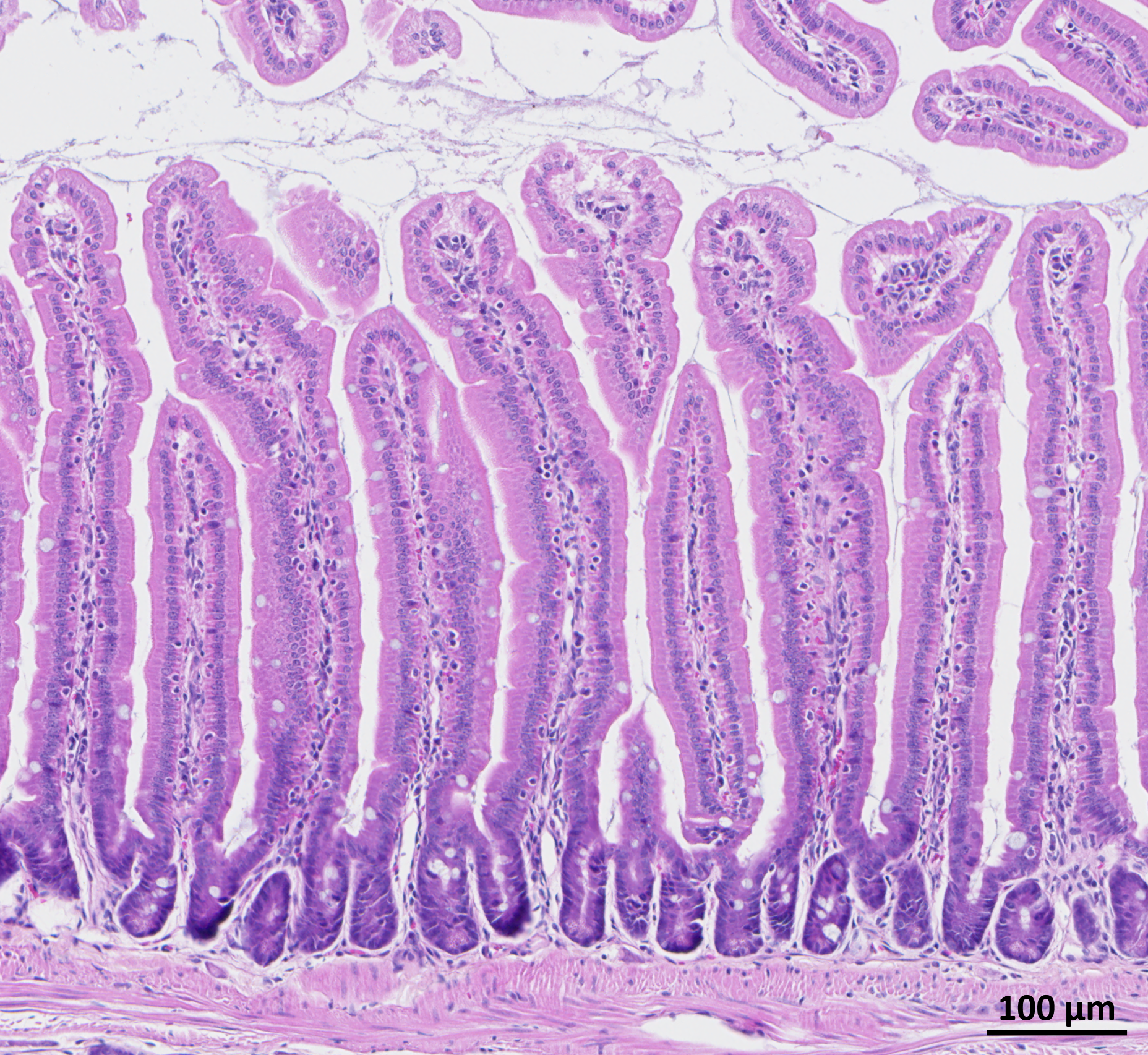

Supplement: Supplementary file 7 — Source data Fig. 6 [file 44319_2025_441_MOESM7_ESM.zip › Figure 6/6F/Duodenum_WT_Control_H&E_R88_mouse 10.tif]

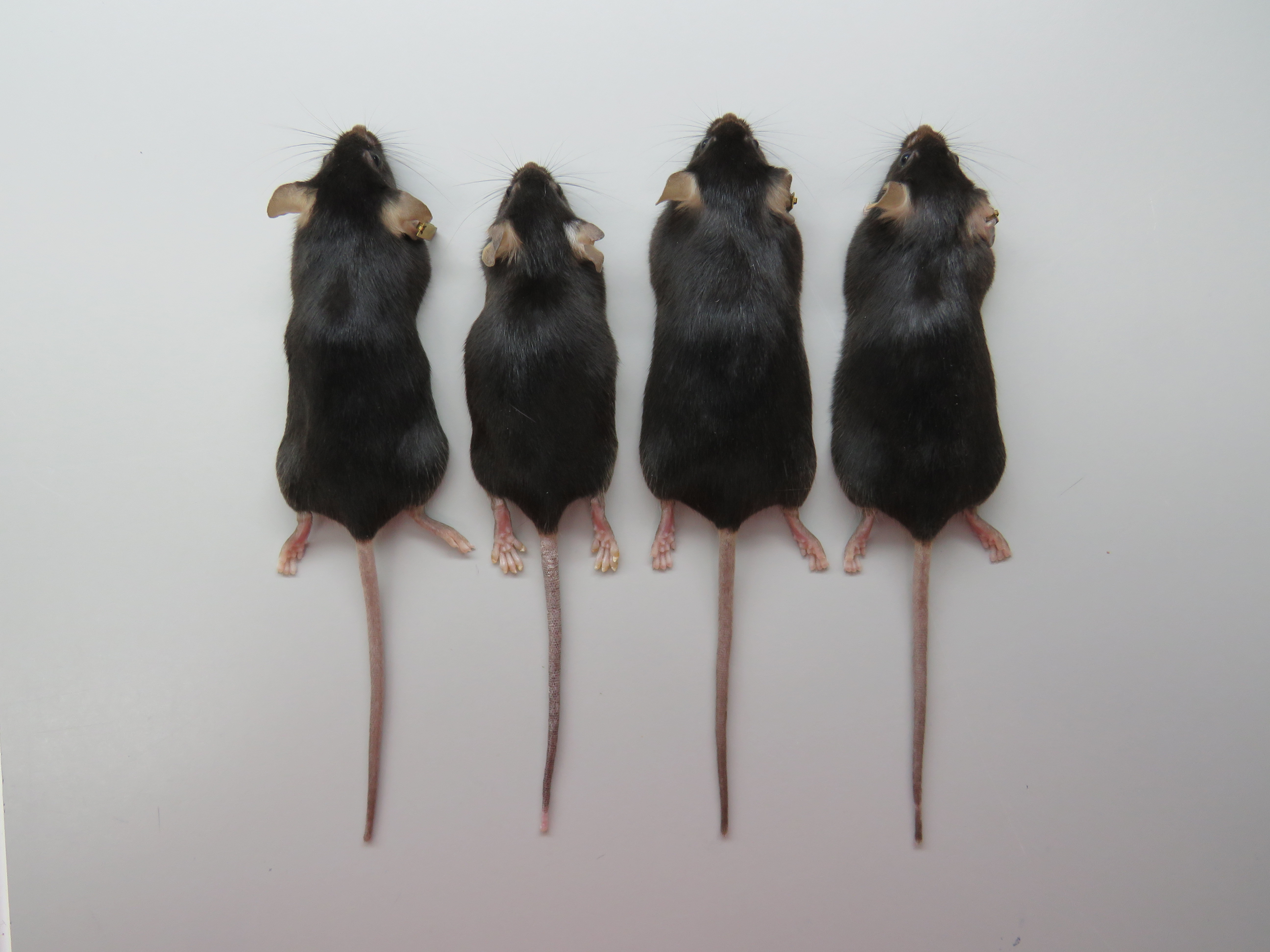

Supplement: Supplementary file 8 — Source data Fig. 7 [file 44319_2025_441_MOESM8_ESM.zip › Figure 7/7A/IMG_7717.JPG]

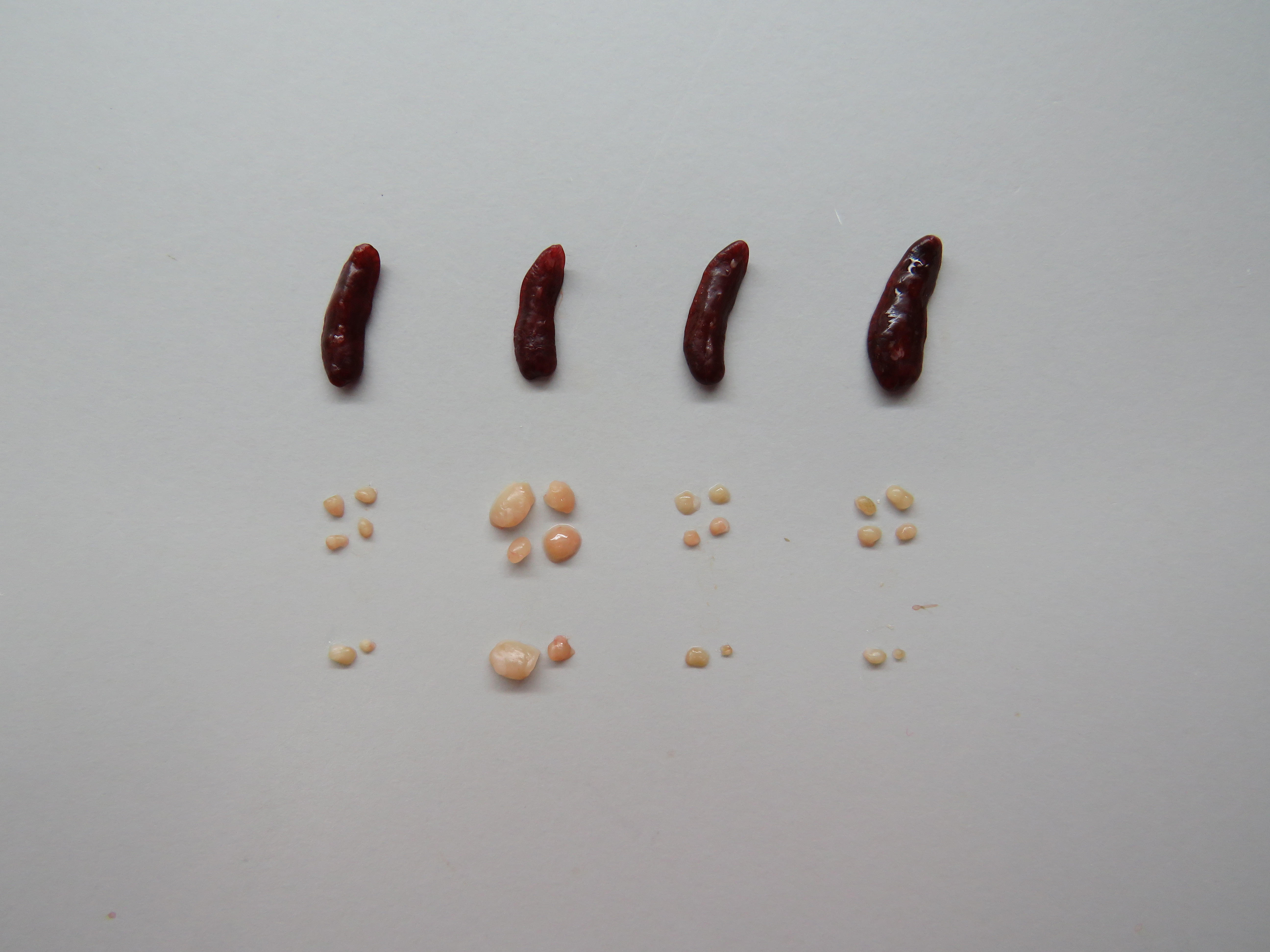

Supplement: Supplementary file 8 — Source data Fig. 7 [file 44319_2025_441_MOESM8_ESM.zip › Figure 7/7B/IMG_7731.JPG]

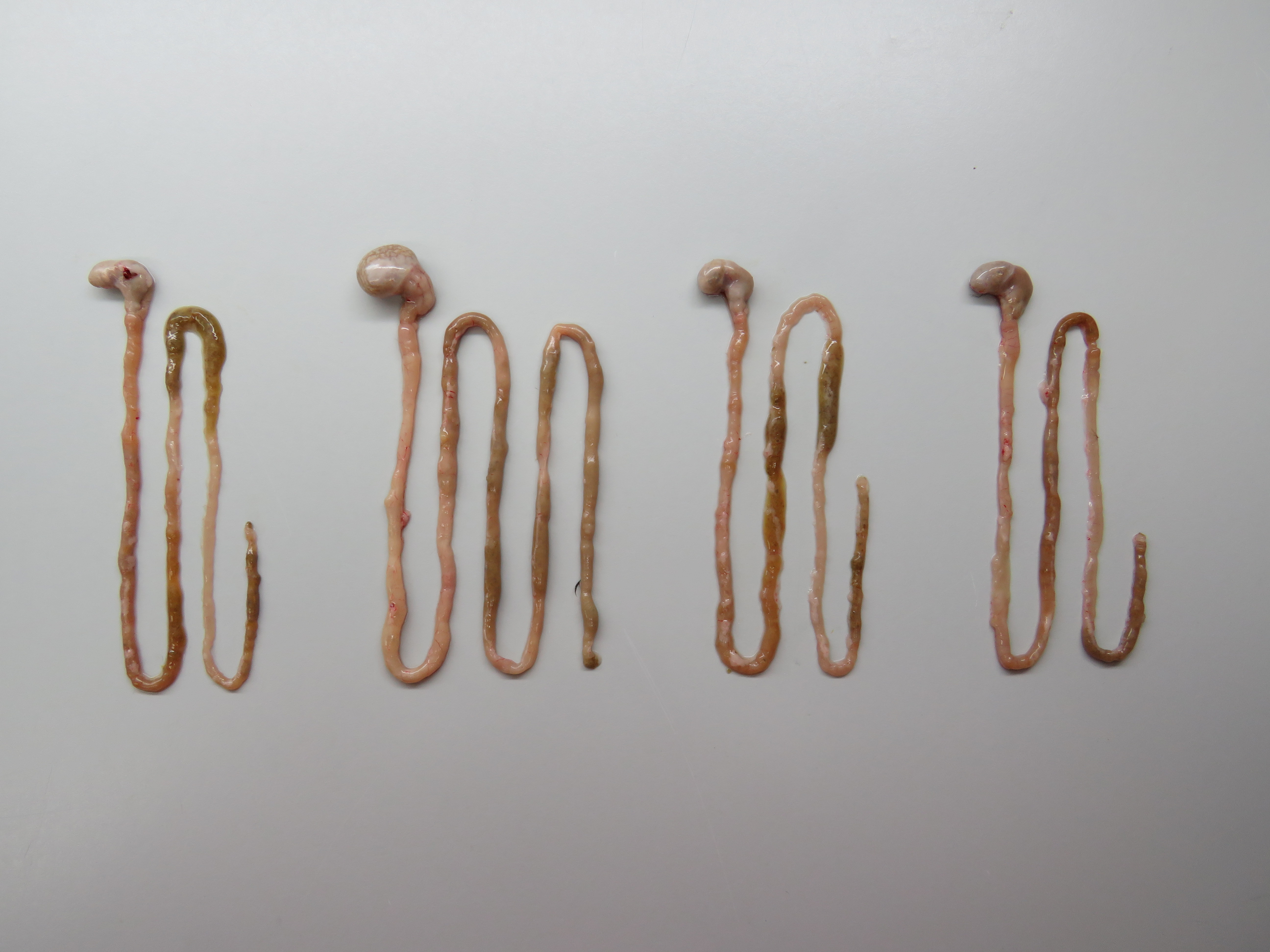

Supplement: Supplementary file 8 — Source data Fig. 7 [file 44319_2025_441_MOESM8_ESM.zip › Figure 7/7C/IMG_7733.JPG]

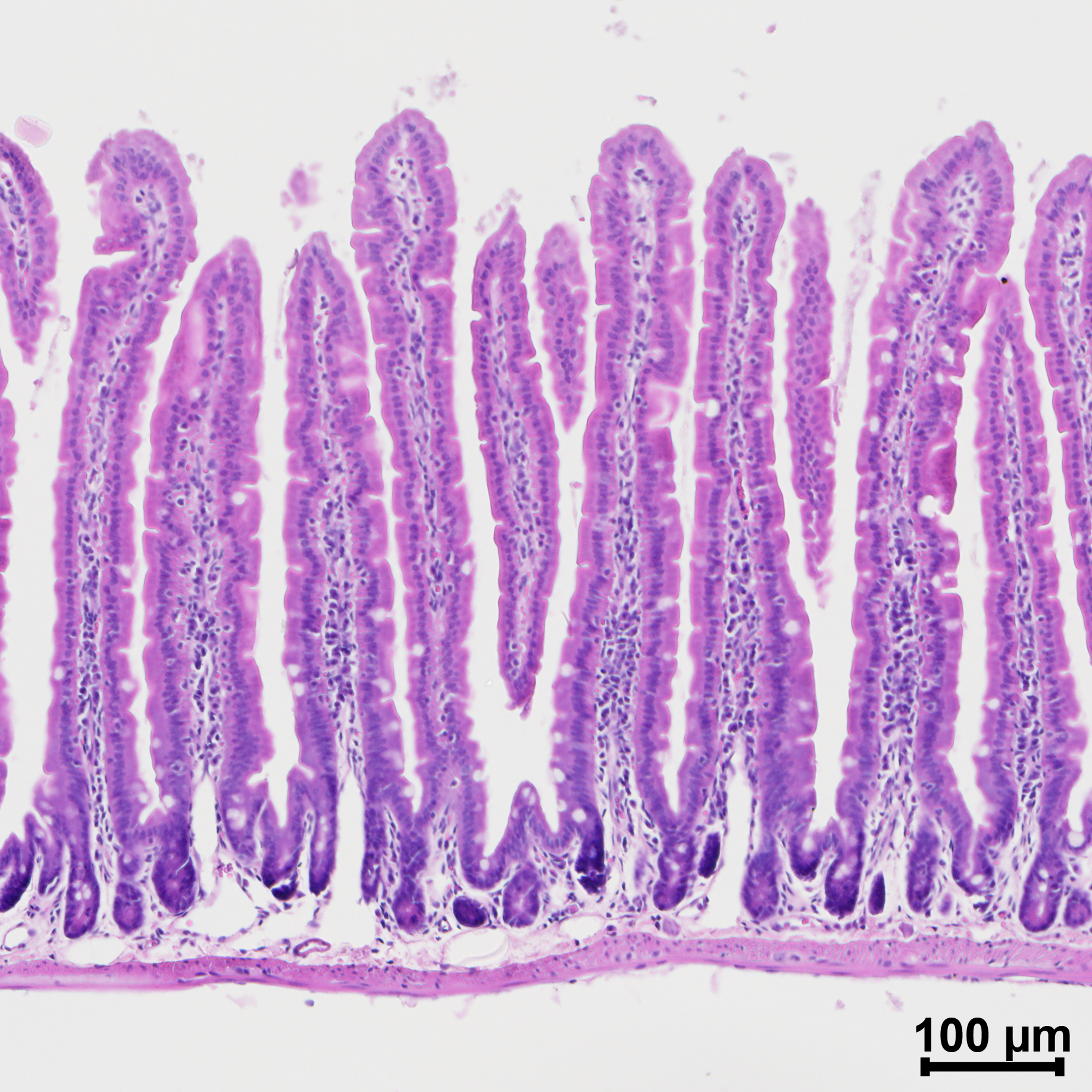

Supplement: Supplementary file 8 — Source data Fig. 7 [file 44319_2025_441_MOESM8_ESM.zip › Figure 7/7E/Duodenum_H&E_R117_mouse 1_DWT.bmp]

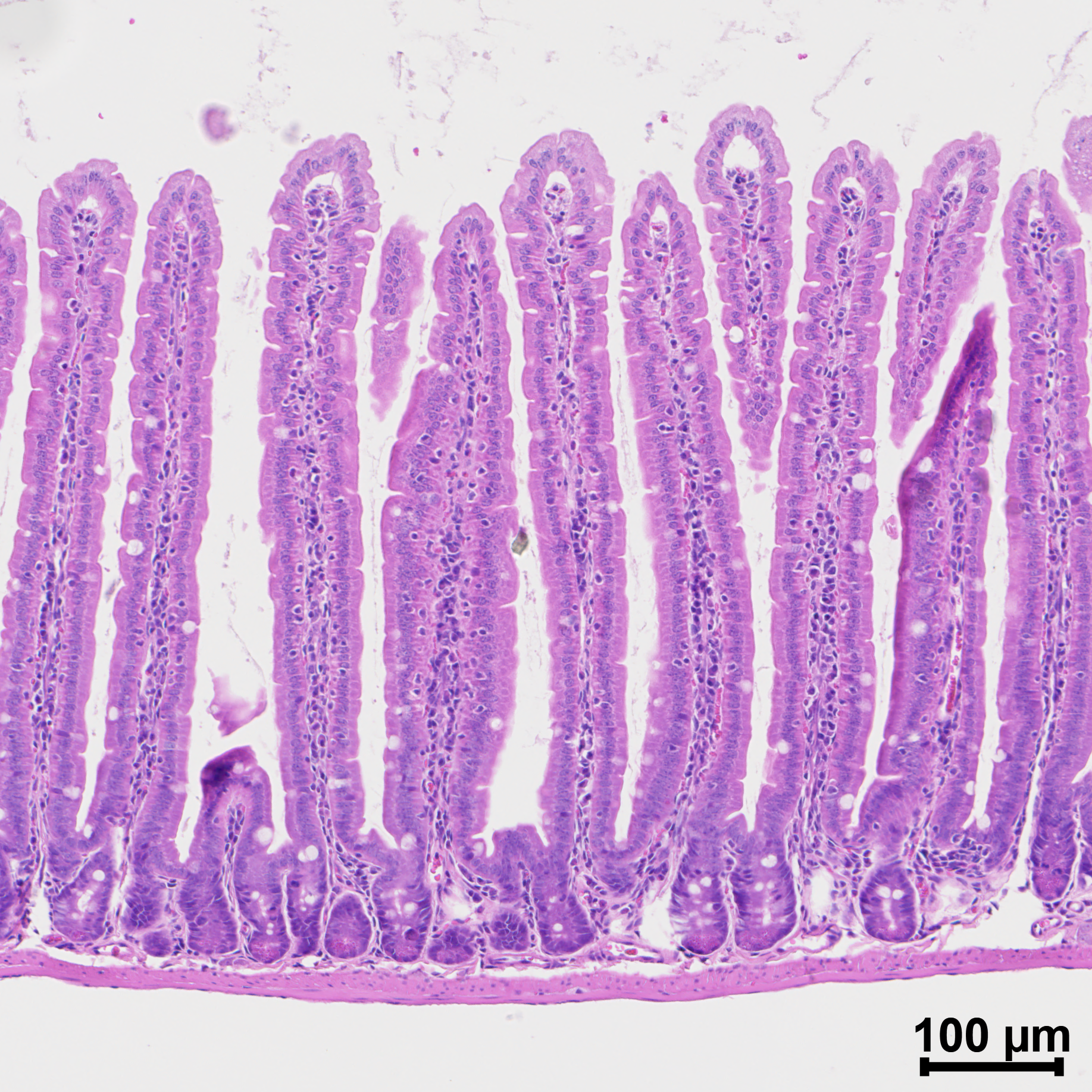

Supplement: Supplementary file 8 — Source data Fig. 7 [file 44319_2025_441_MOESM8_ESM.zip › Figure 7/7E/Duodenum_H&E_R117_mouse 3_DKO.bmp]

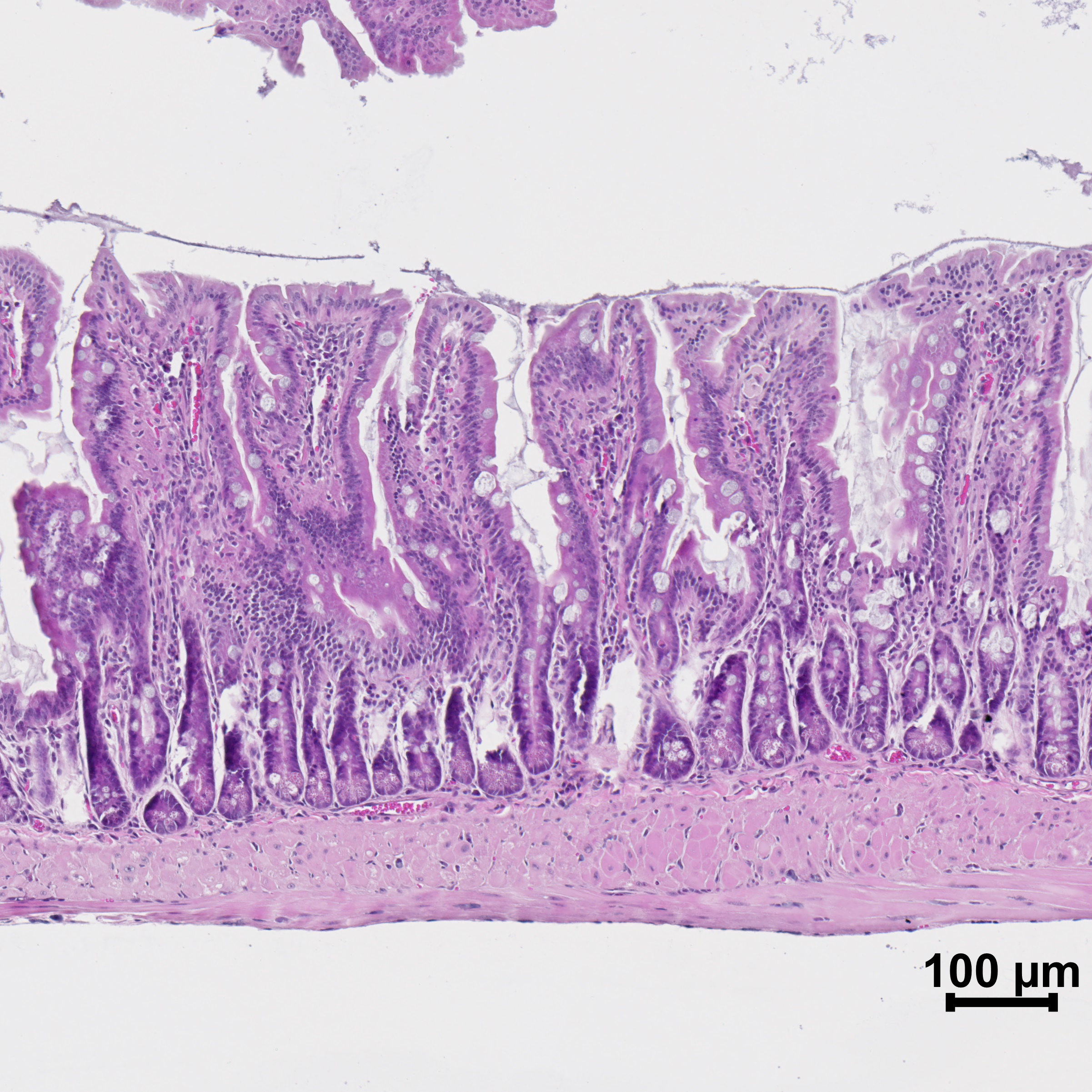

Supplement: Supplementary file 8 — Source data Fig. 7 [file 44319_2025_441_MOESM8_ESM.zip › Figure 7/7E/Duodenum_H&E_R64_mouse 11_KO.jpg]

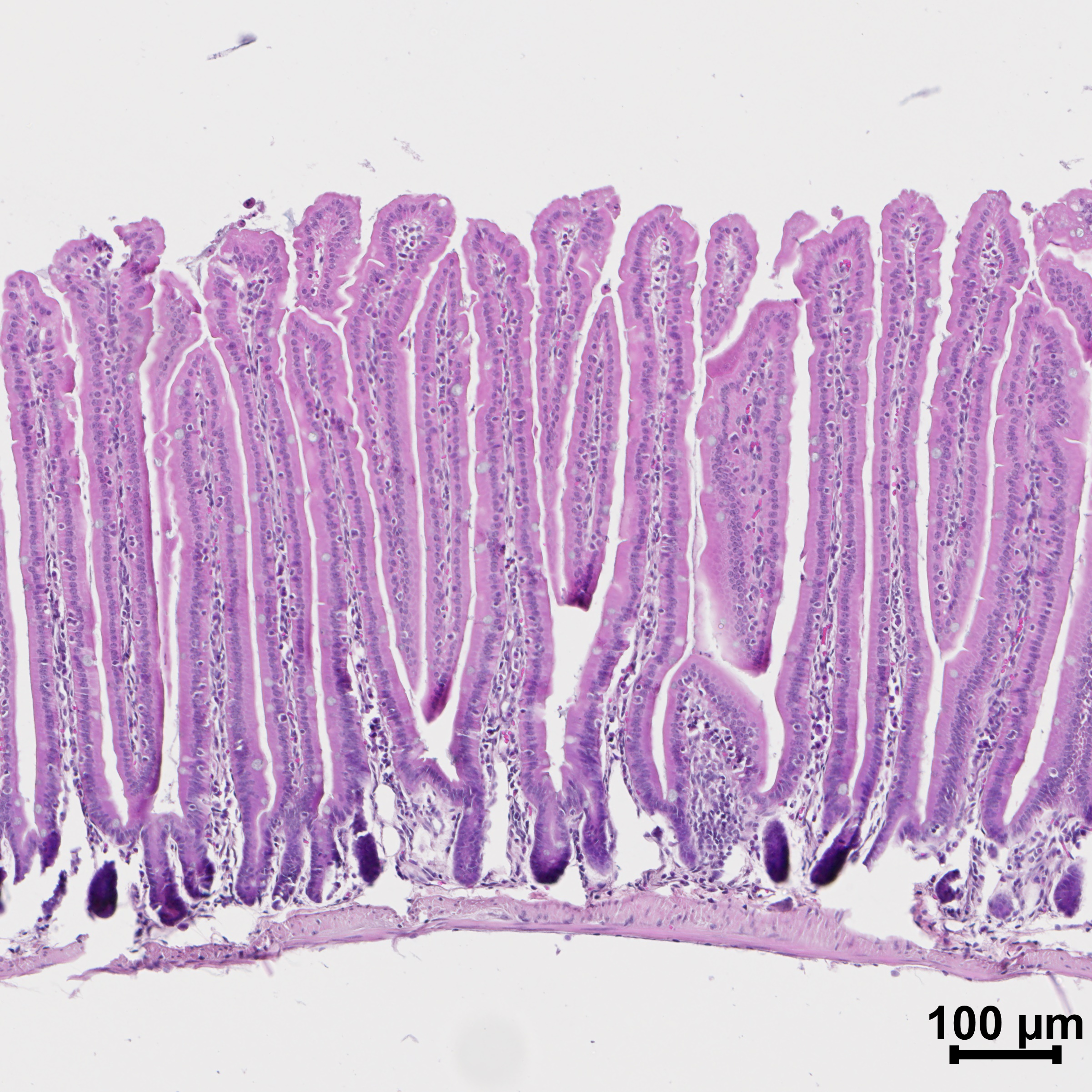

Supplement: Supplementary file 8 — Source data Fig. 7 [file 44319_2025_441_MOESM8_ESM.zip › Figure 7/7E/Duodenum_H&E_R64_mouse 3_WT.jpg]

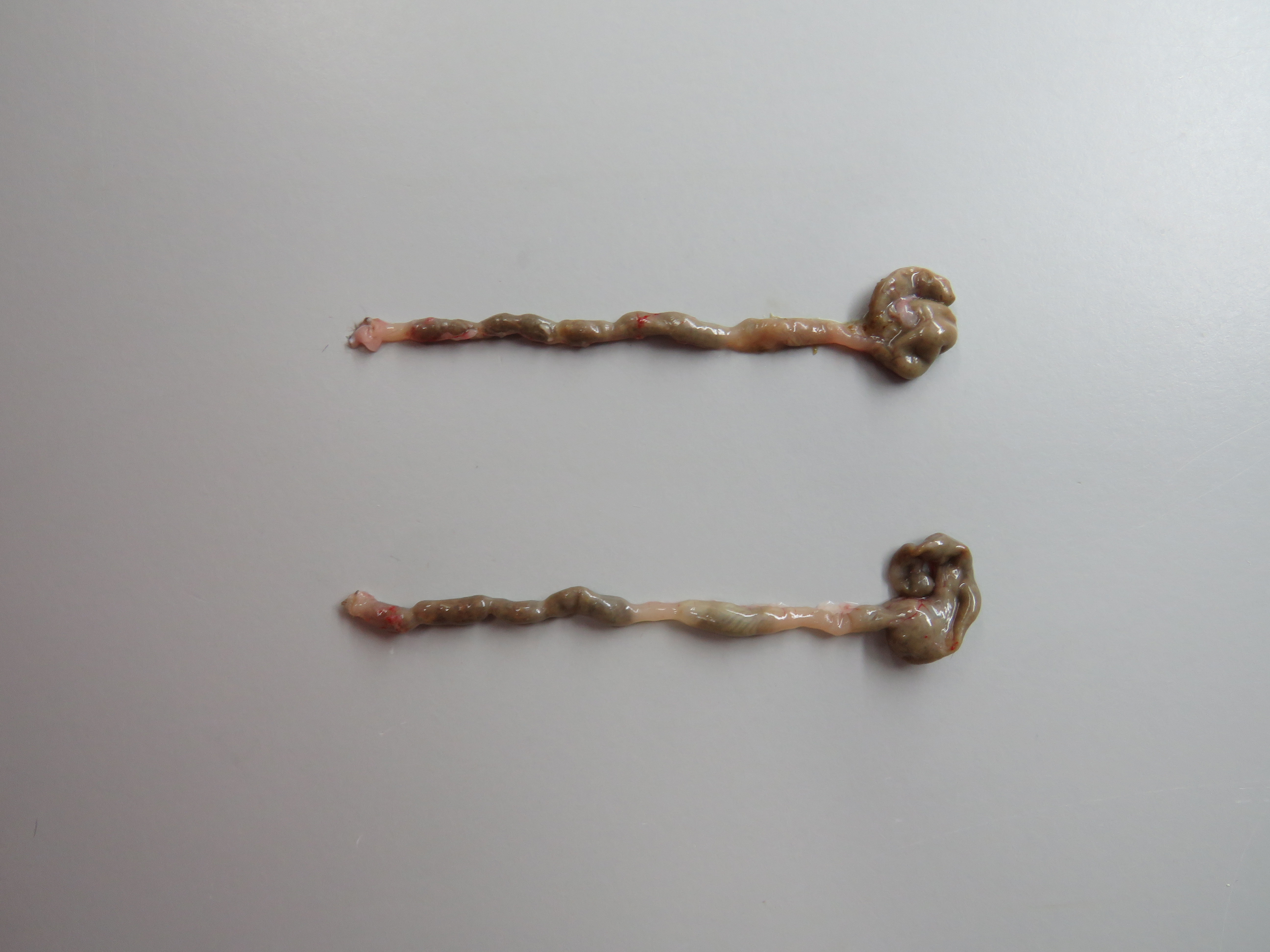

Supplement: Supplementary file 9 — Figure EV1-EV5 Source Data [file 44319_2025_441_MOESM9_ESM.zip › EV Figures/Figure EV1/EV1C/IMG_7715.JPG]

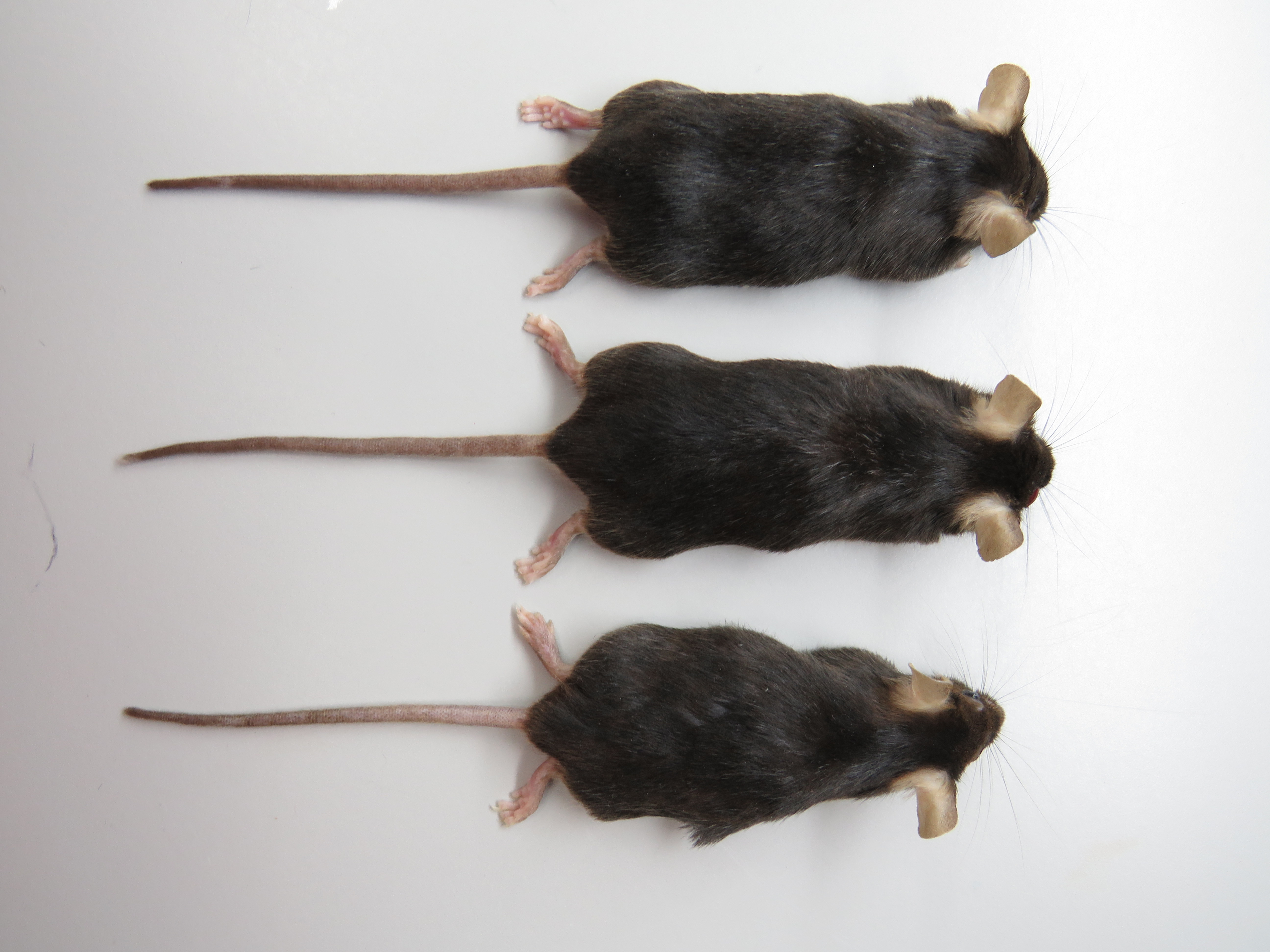

Supplement: Supplementary file 9 — Figure EV1-EV5 Source Data [file 44319_2025_441_MOESM9_ESM.zip › EV Figures/Figure EV1/EV1E/IMG_5757.JPG]

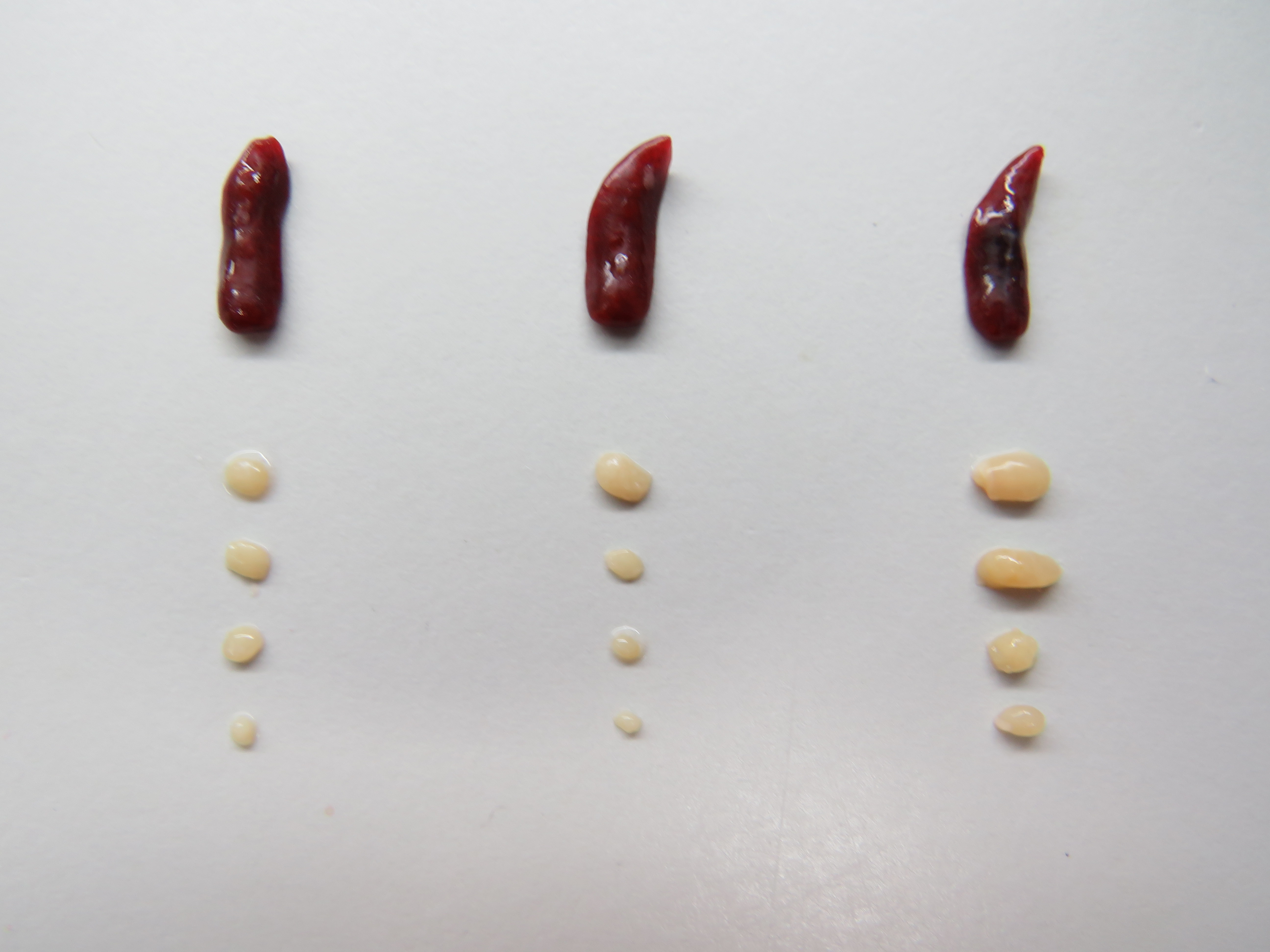

Supplement: Supplementary file 9 — Figure EV1-EV5 Source Data [file 44319_2025_441_MOESM9_ESM.zip › EV Figures/Figure EV1/EV1E/IMG_5759.JPG]

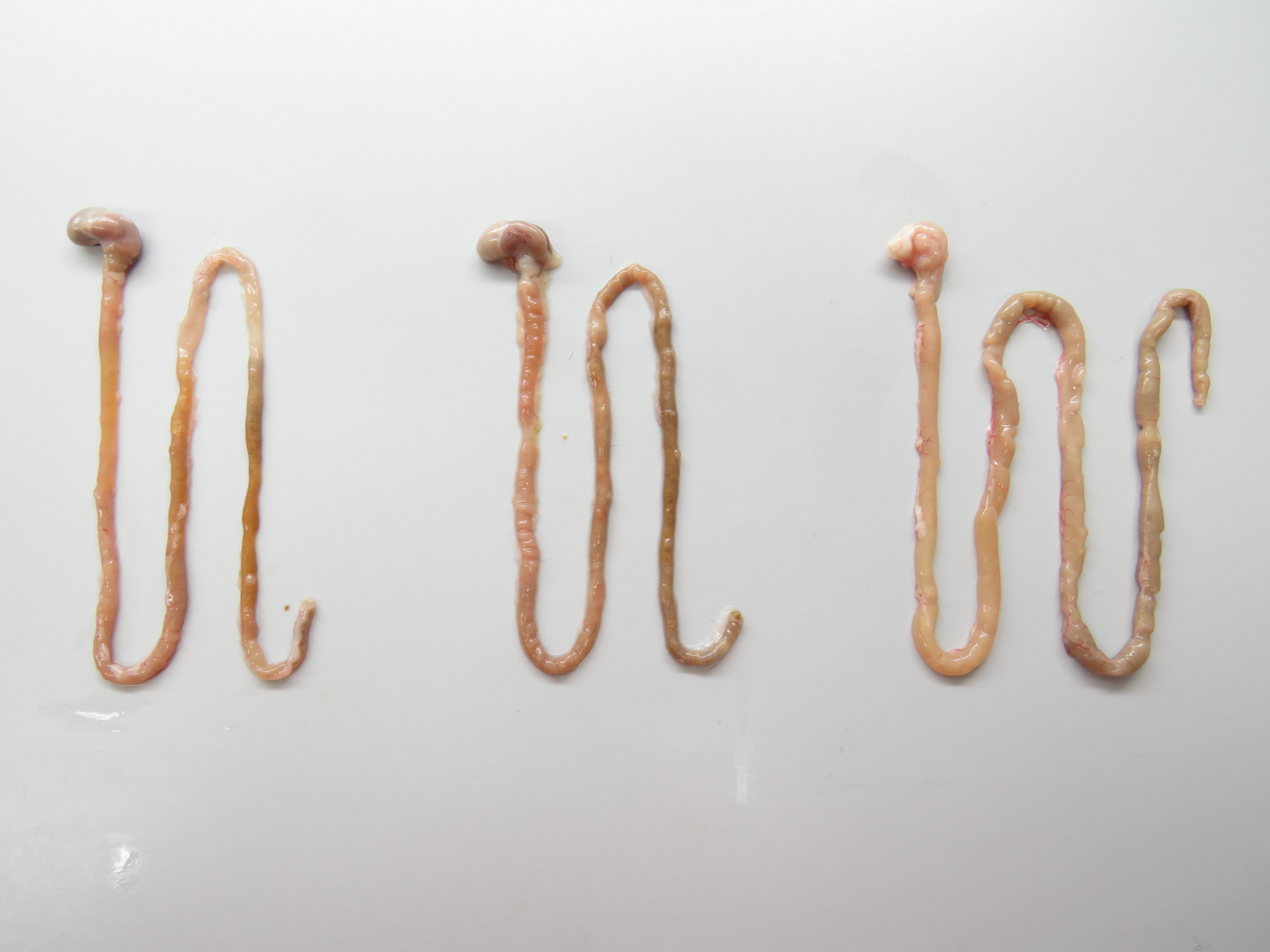

Supplement: Supplementary file 9 — Figure EV1-EV5 Source Data [file 44319_2025_441_MOESM9_ESM.zip › EV Figures/Figure EV1/EV1E/IMG_5771.JPG]

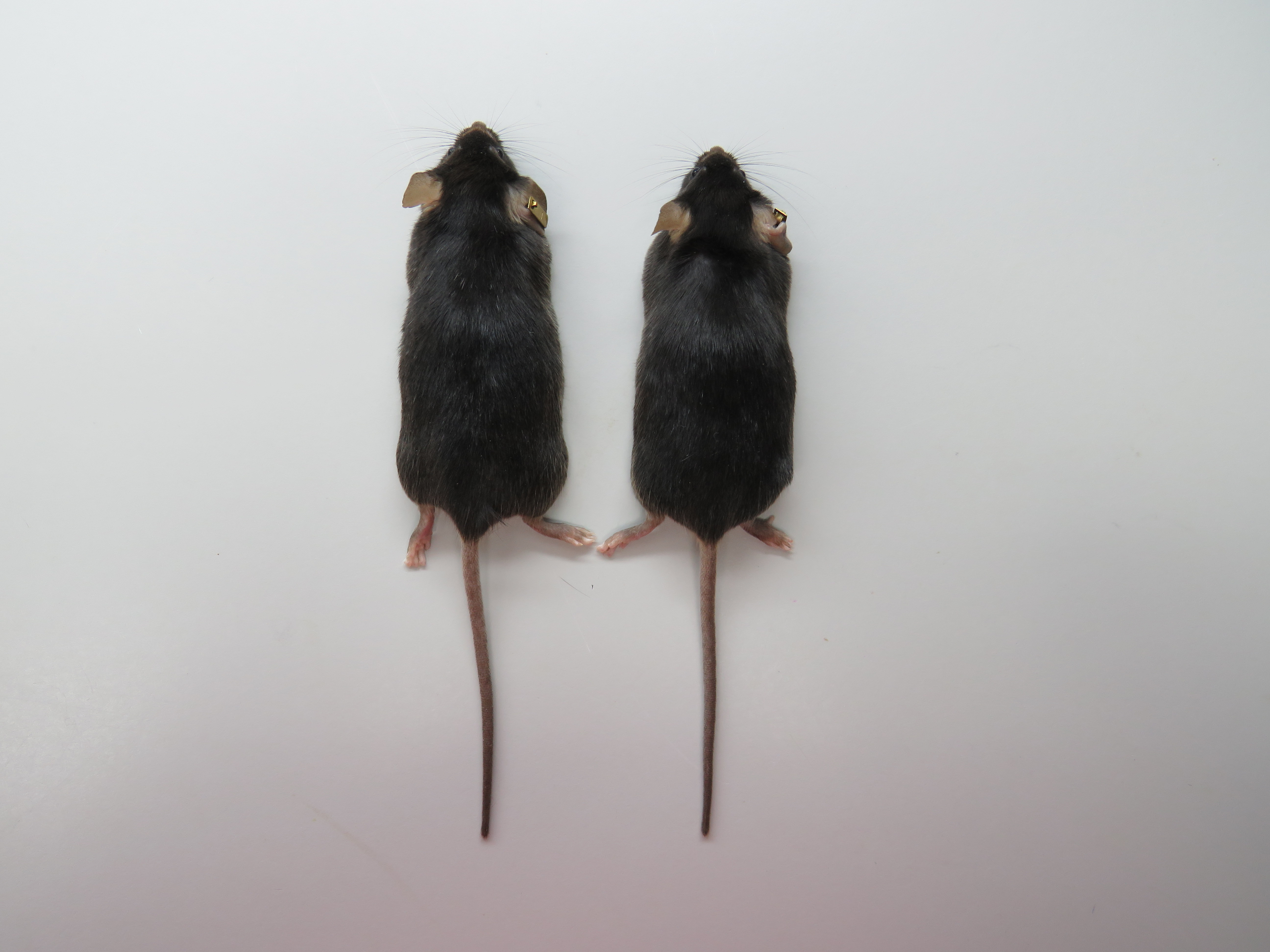

Supplement: Supplementary file 9 — Figure EV1-EV5 Source Data [file 44319_2025_441_MOESM9_ESM.zip › EV Figures/Figure EV1/EV1F/IMG_7165.JPG]

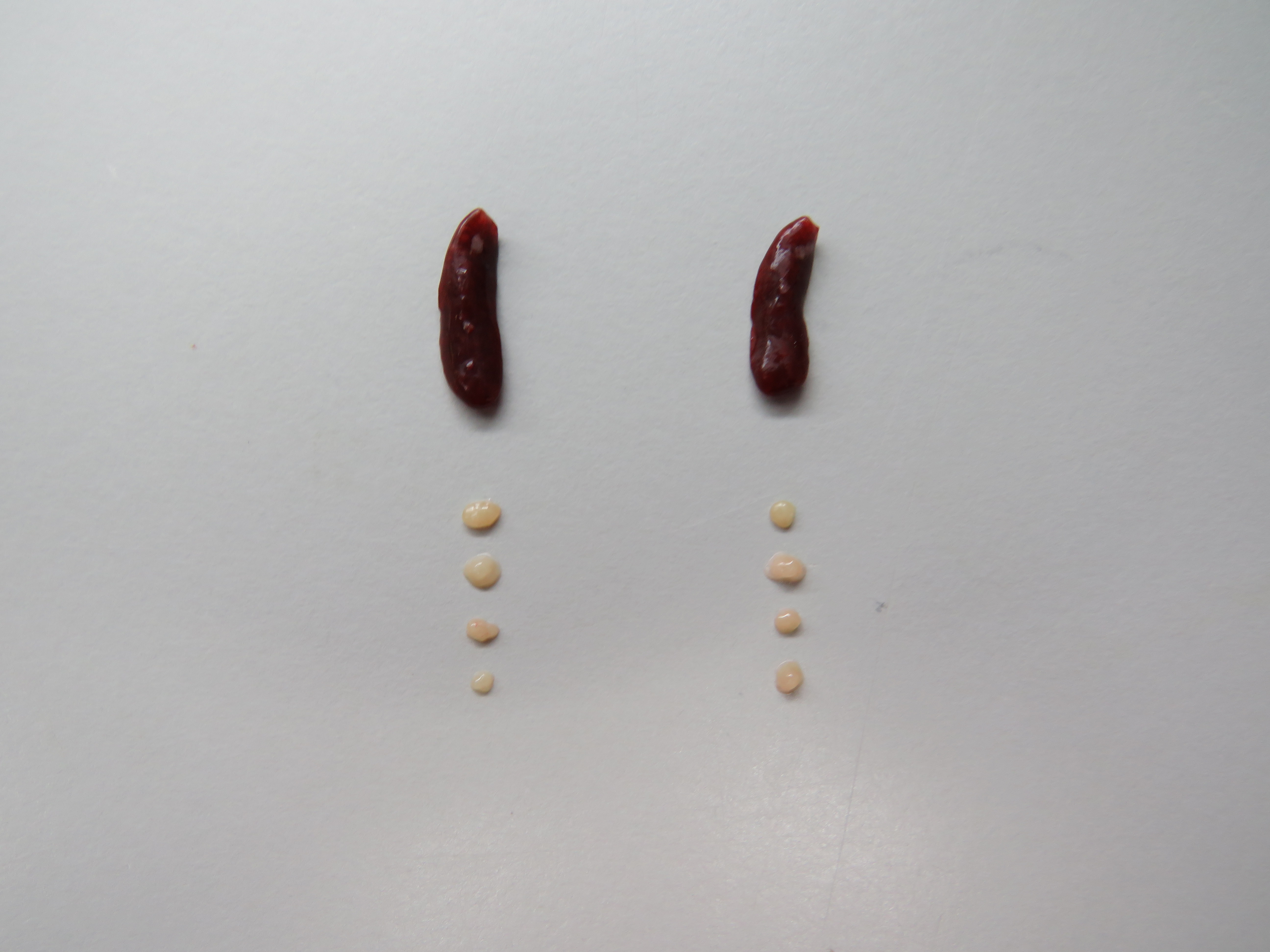

Supplement: Supplementary file 9 — Figure EV1-EV5 Source Data [file 44319_2025_441_MOESM9_ESM.zip › EV Figures/Figure EV1/EV1F/IMG_7187.JPG]

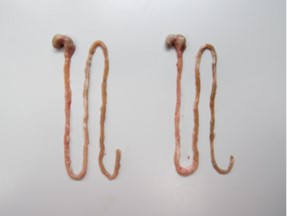

Supplement: Supplementary file 9 — Figure EV1-EV5 Source Data [file 44319_2025_441_MOESM9_ESM.zip › EV Figures/Figure EV1/EV1F/IMG_7203.jpg]

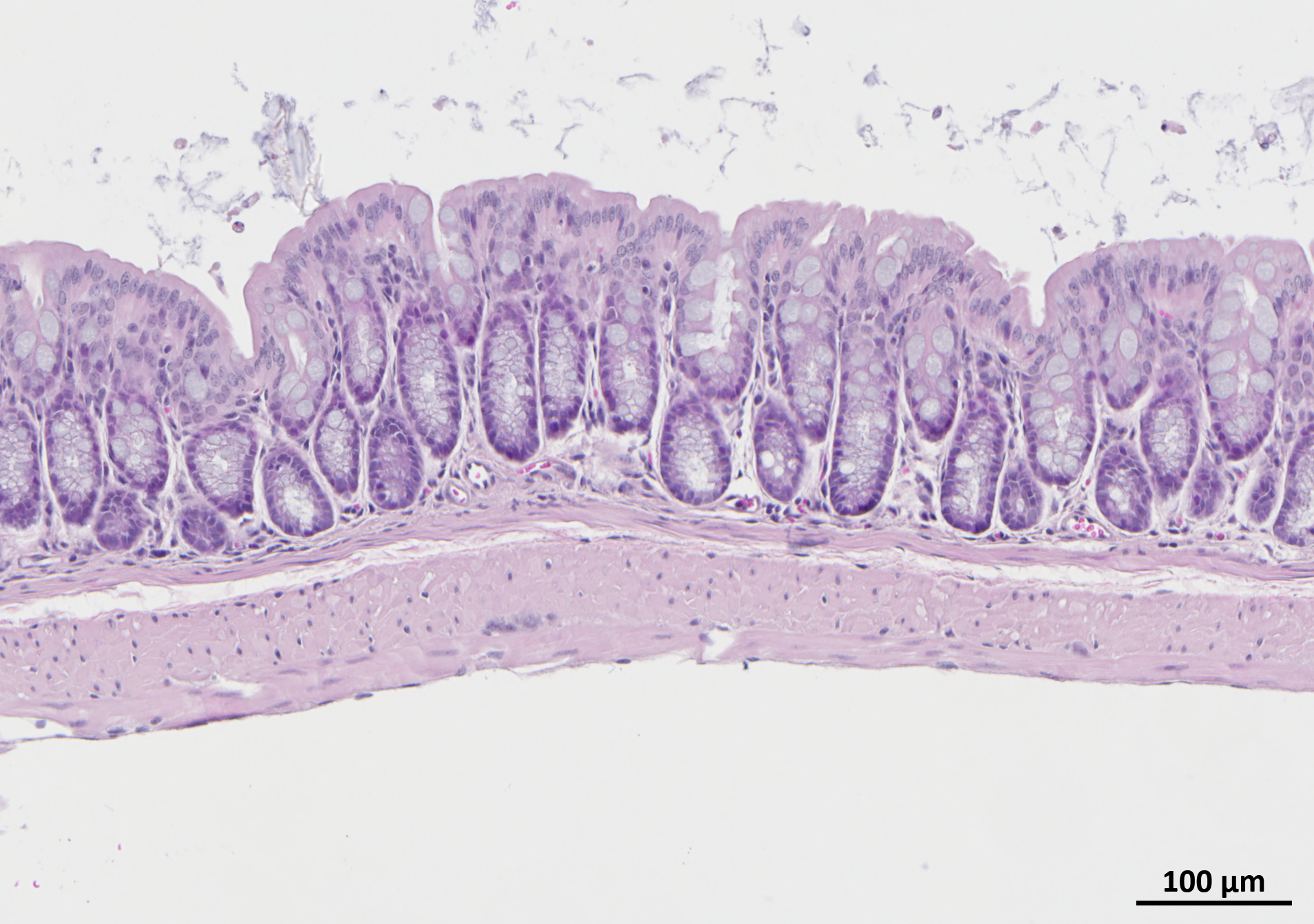

Supplement: Supplementary file 9 — Figure EV1-EV5 Source Data [file 44319_2025_441_MOESM9_ESM.zip › EV Figures/Figure EV2/EV2C/R64_mouse 10_Colon.tif]

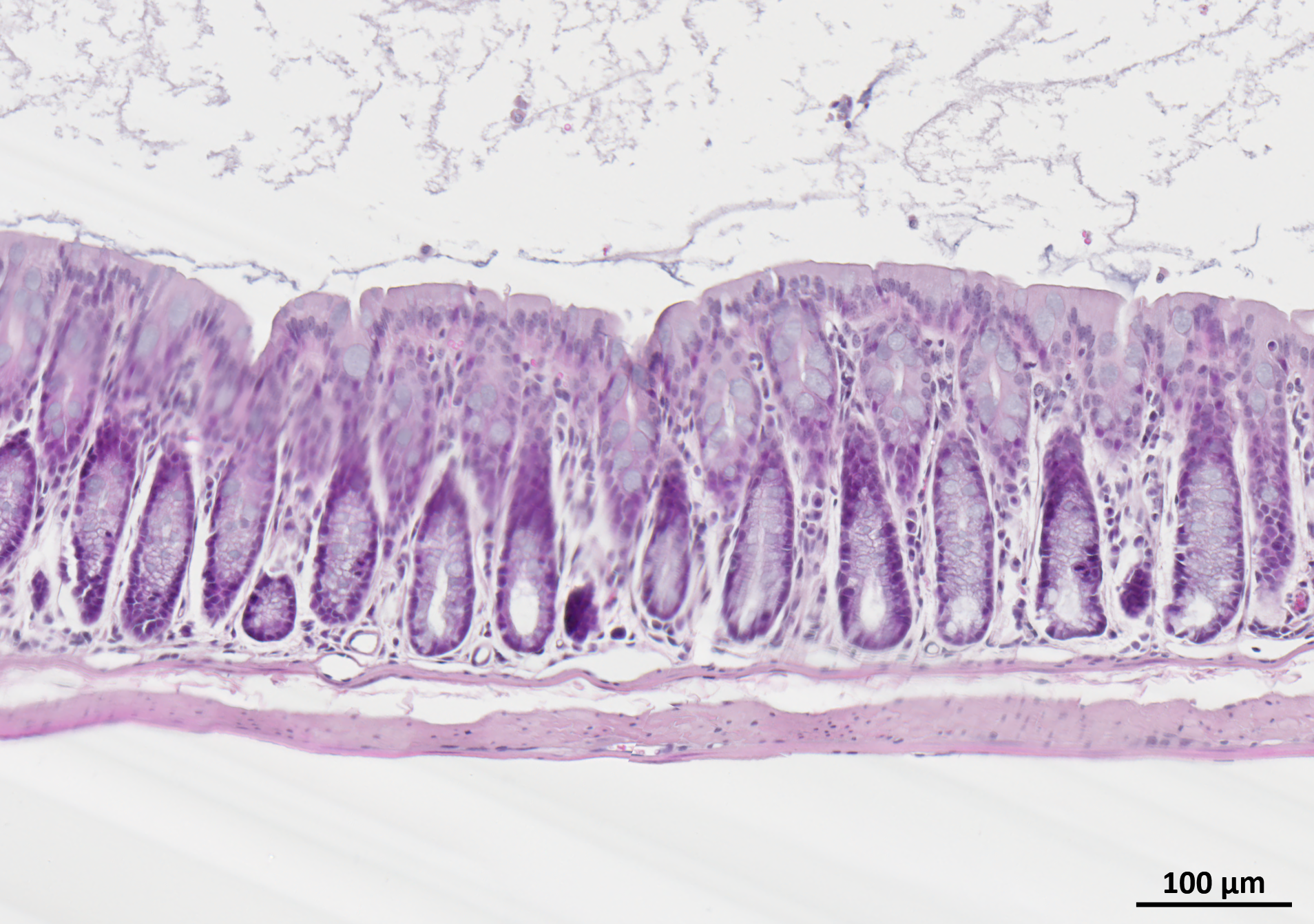

Supplement: Supplementary file 9 — Figure EV1-EV5 Source Data [file 44319_2025_441_MOESM9_ESM.zip › EV Figures/Figure EV2/EV2C/R64_mouse 8_Colon.tif]

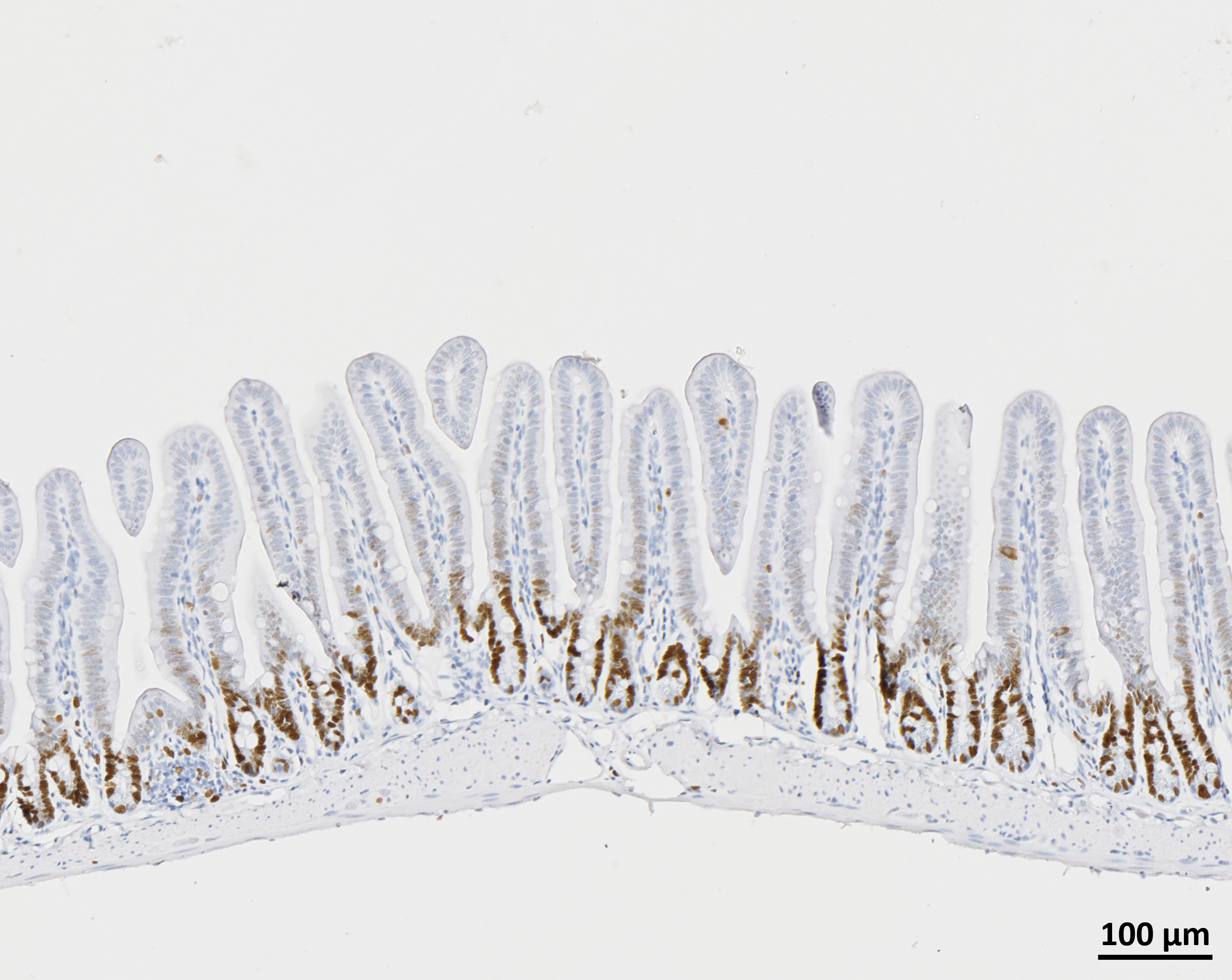

Supplement: Supplementary file 9 — Figure EV1-EV5 Source Data [file 44319_2025_441_MOESM9_ESM.zip › EV Figures/Figure EV2/EV2D/Ileum_Ki67_R64_mouse 4_WT.tif]

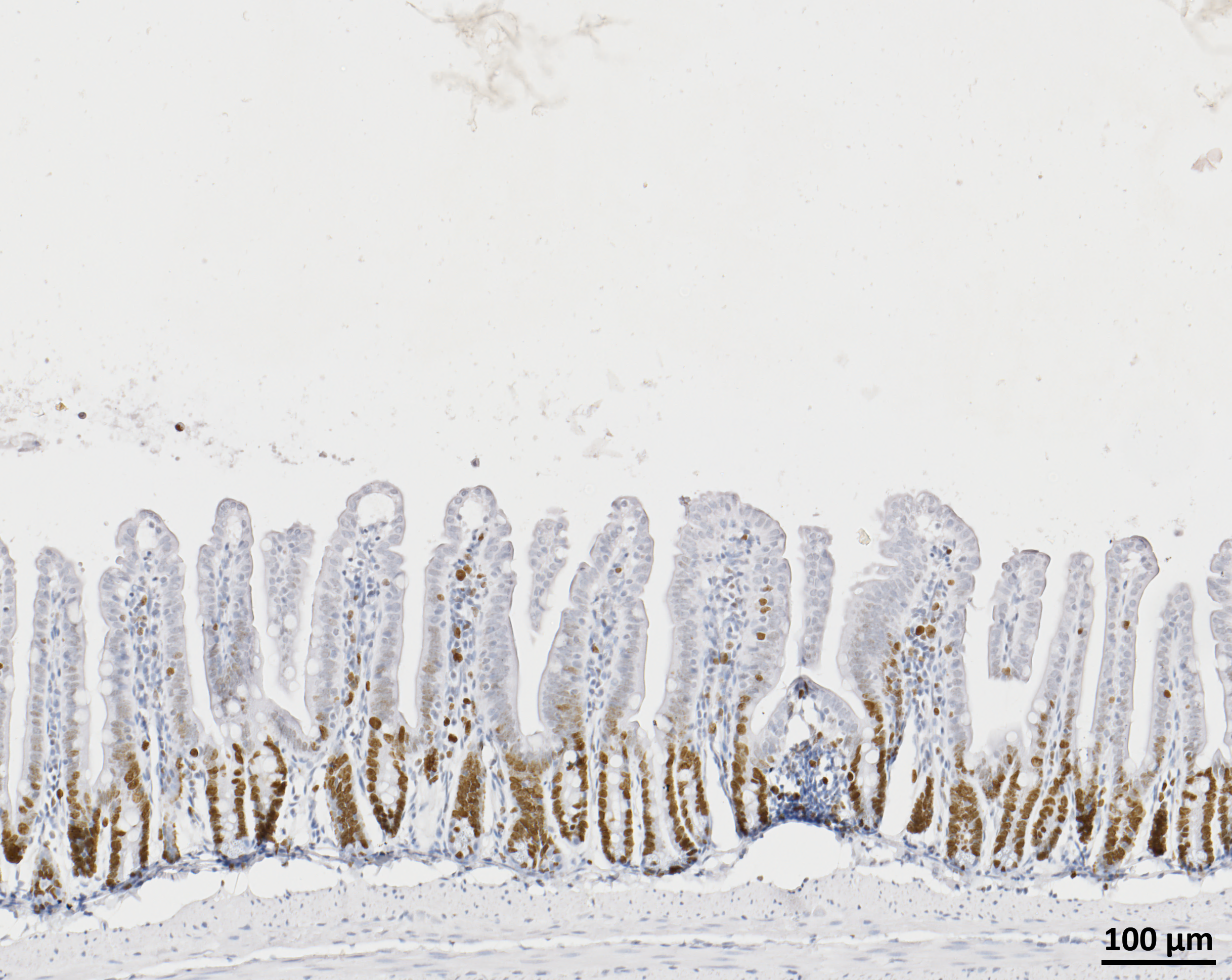

Supplement: Supplementary file 9 — Figure EV1-EV5 Source Data [file 44319_2025_441_MOESM9_ESM.zip › EV Figures/Figure EV2/EV2D/Ileum_Ki67_R64_mouse 5_KO.tif]

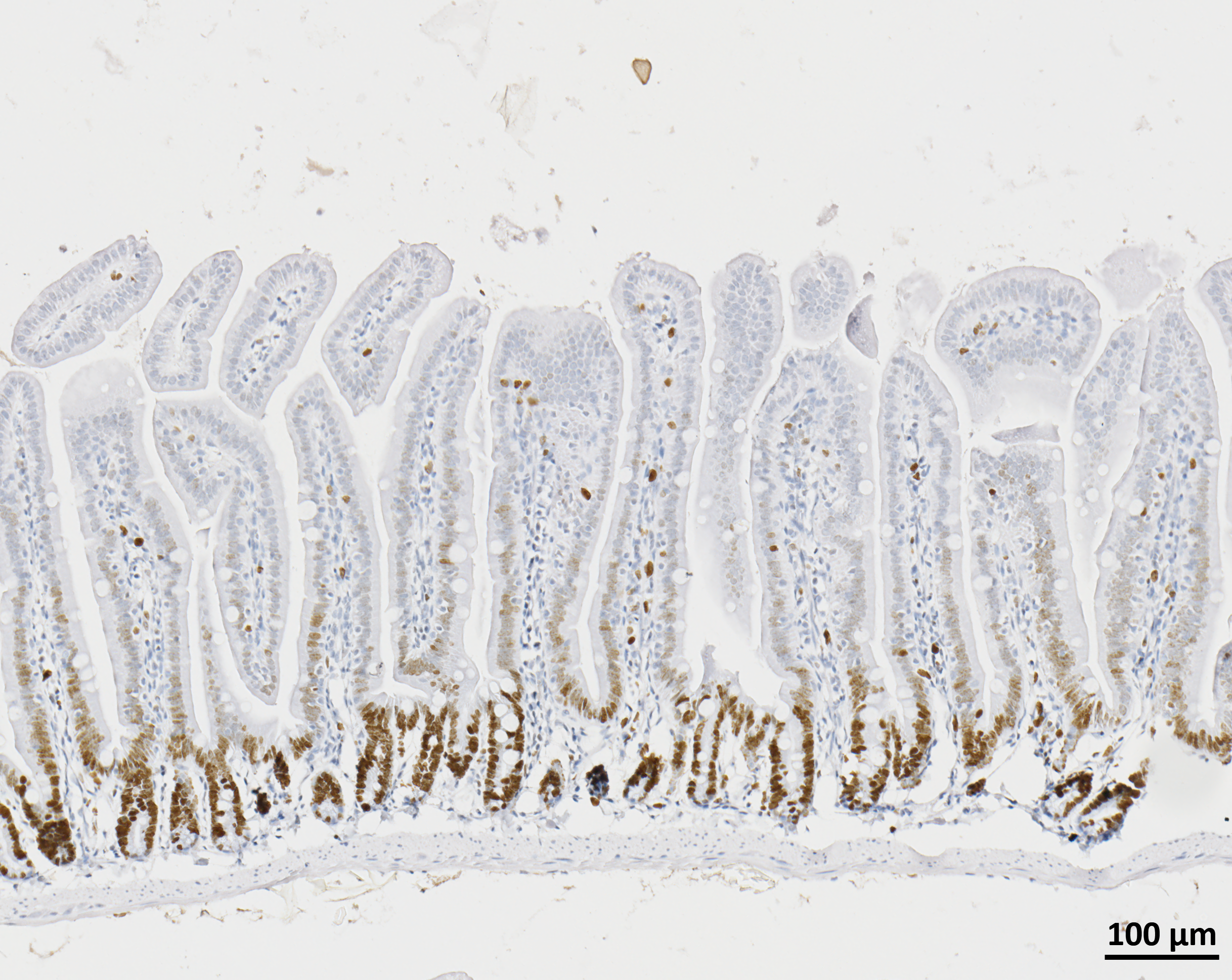

Supplement: Supplementary file 9 — Figure EV1-EV5 Source Data [file 44319_2025_441_MOESM9_ESM.zip › EV Figures/Figure EV2/EV2D/Jejunum_Ki67_R64_mouse 4_WT.tif]

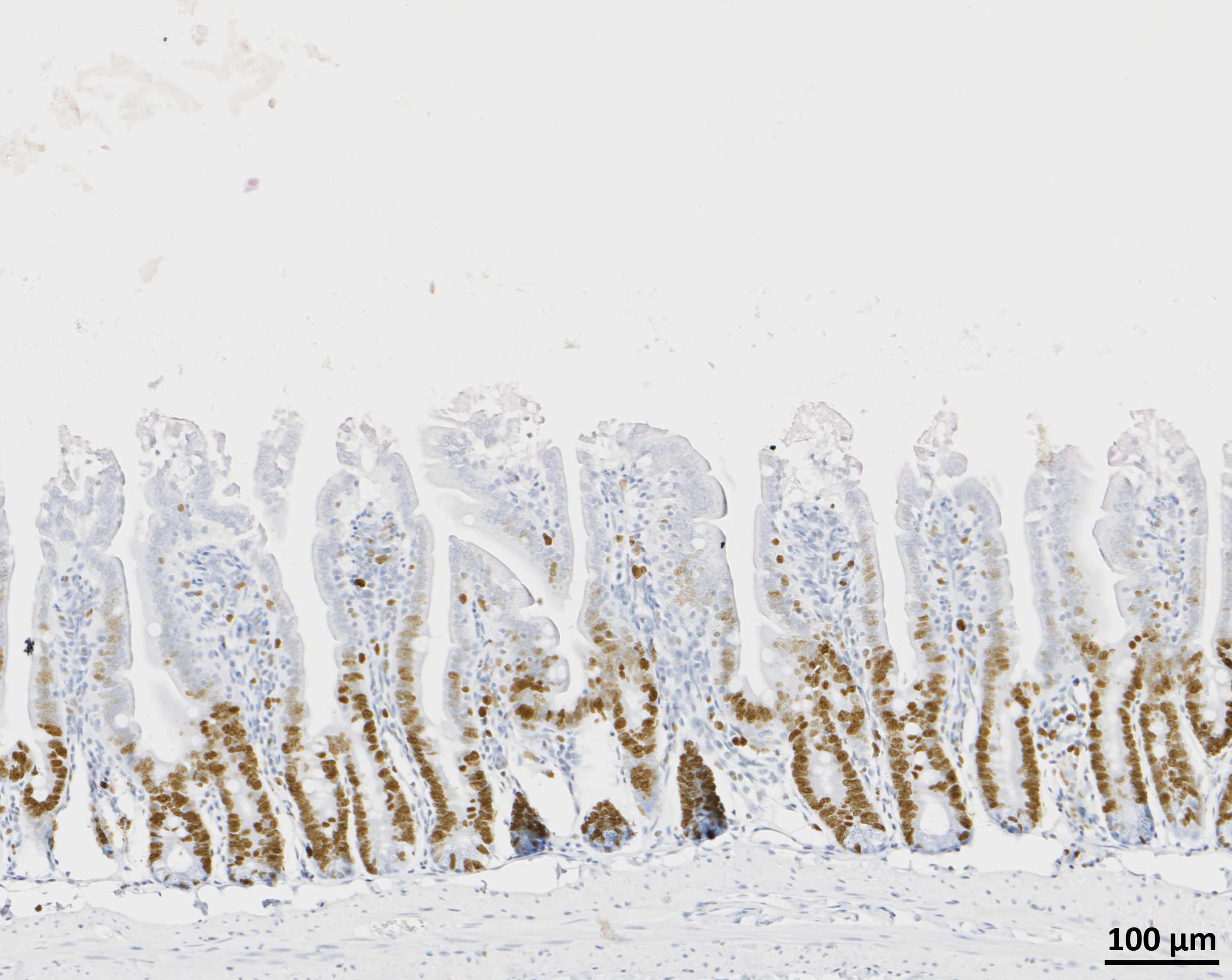

Supplement: Supplementary file 9 — Figure EV1-EV5 Source Data [file 44319_2025_441_MOESM9_ESM.zip › EV Figures/Figure EV2/EV2D/Jejunum_Ki67_R64_mouse 5_KO.tif]

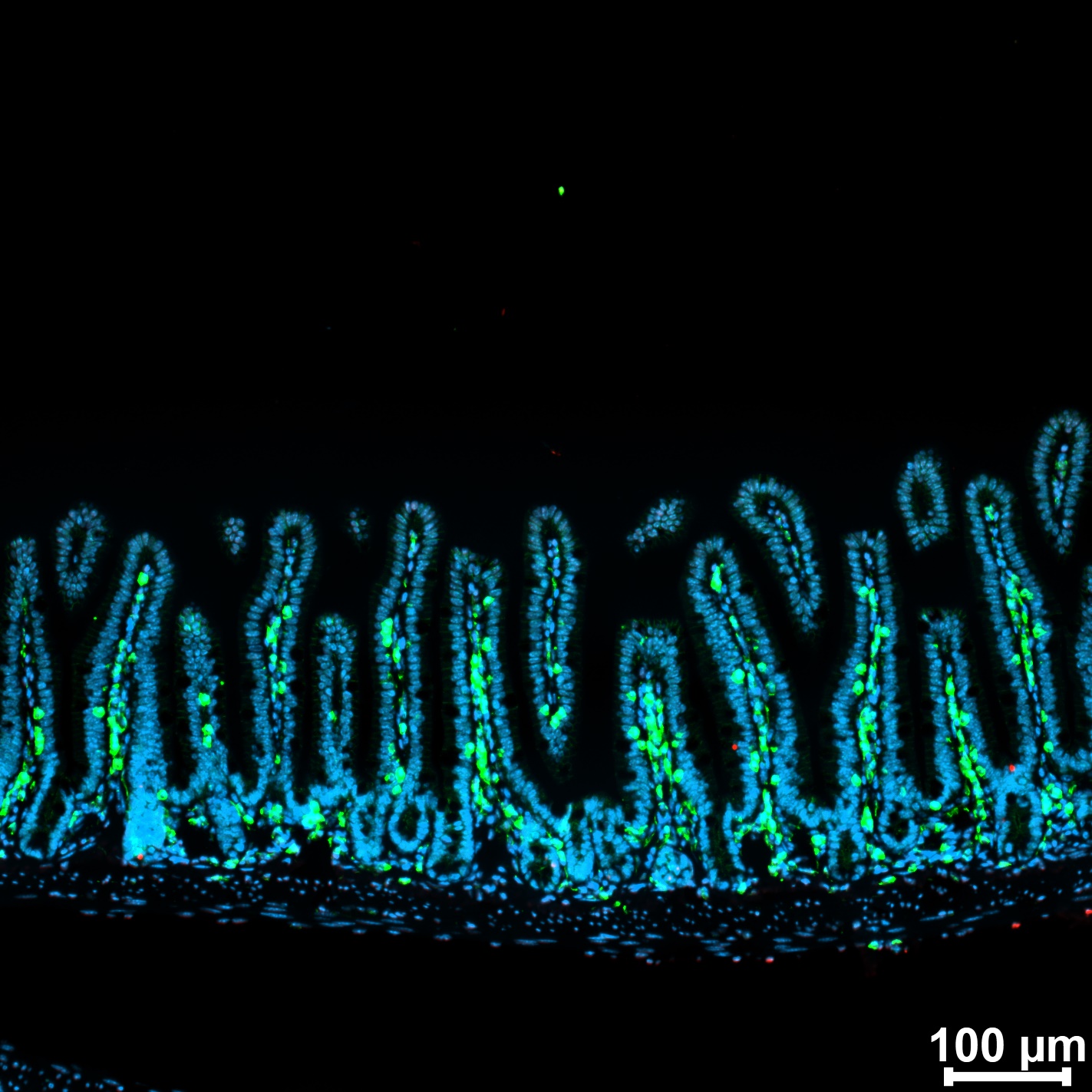

Supplement: Supplementary file 9 — Figure EV1-EV5 Source Data [file 44319_2025_441_MOESM9_ESM.zip › EV Figures/Figure EV2/EV2F/Ileum_CD45_R64_mouse 4_WT.jpg]

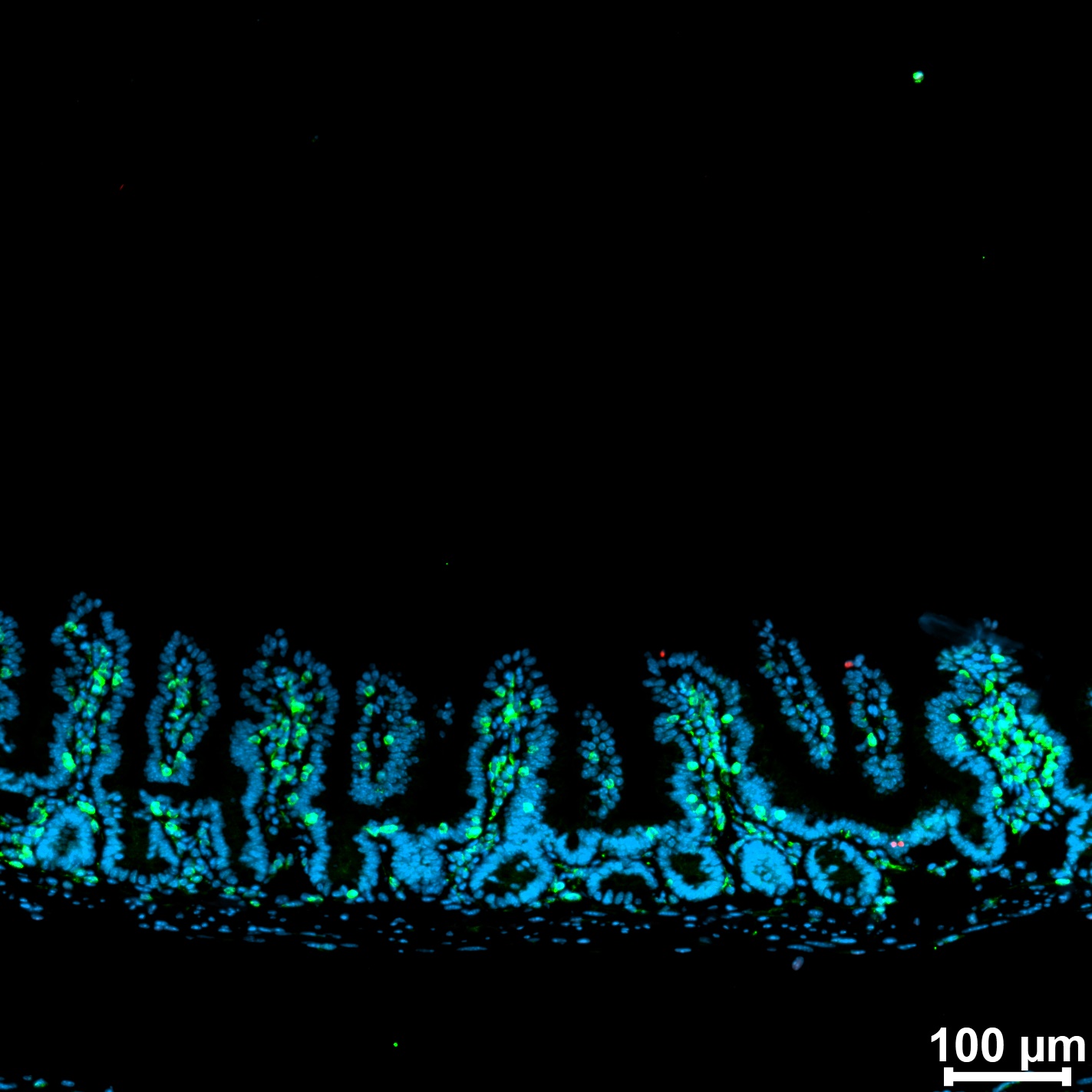

Supplement: Supplementary file 9 — Figure EV1-EV5 Source Data [file 44319_2025_441_MOESM9_ESM.zip › EV Figures/Figure EV2/EV2F/Ileum_CD45_R64_mouse 5_KO.jpg]

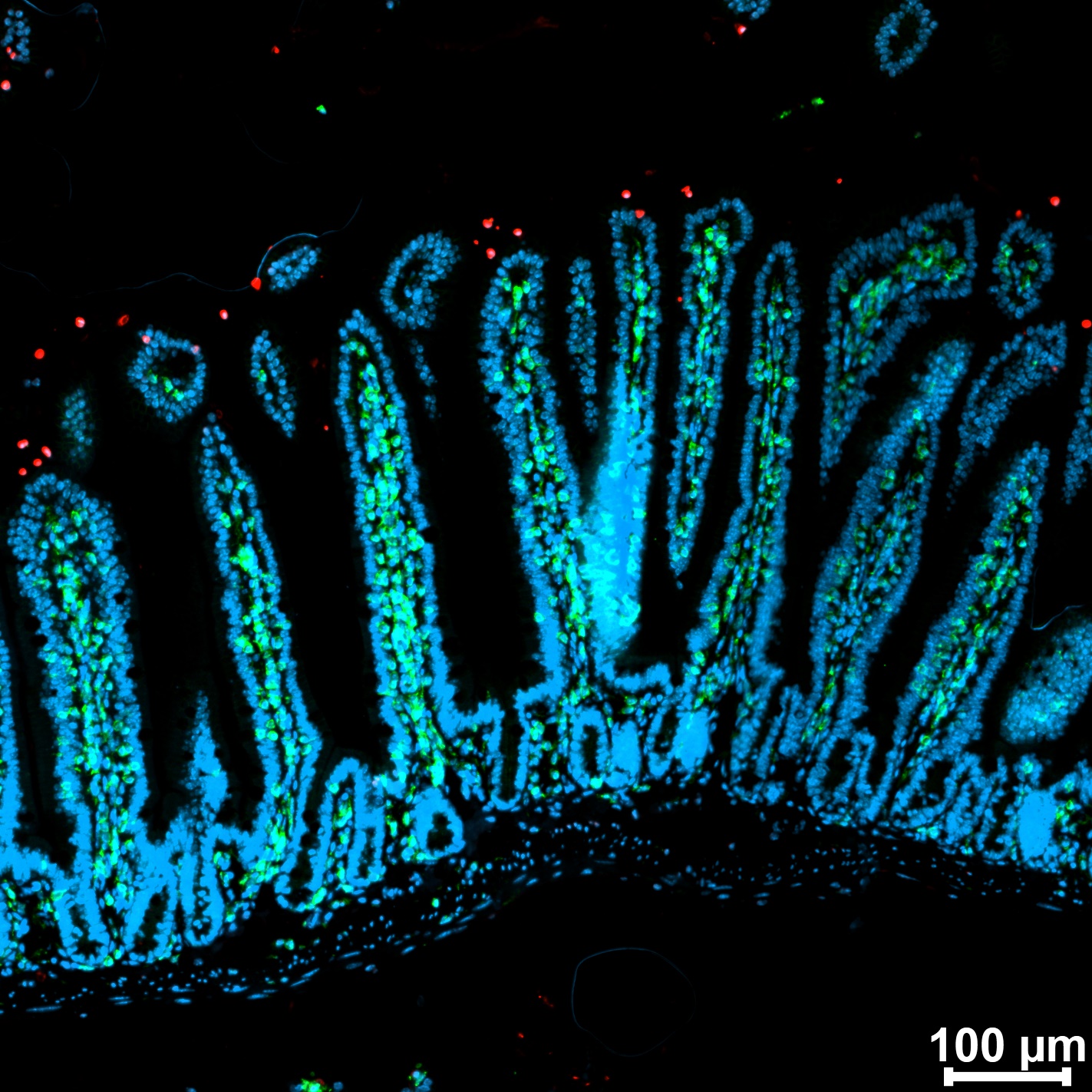

Supplement: Supplementary file 9 — Figure EV1-EV5 Source Data [file 44319_2025_441_MOESM9_ESM.zip › EV Figures/Figure EV2/EV2F/Jejunum_CD45_R64_mouse 4_WT.jpg]

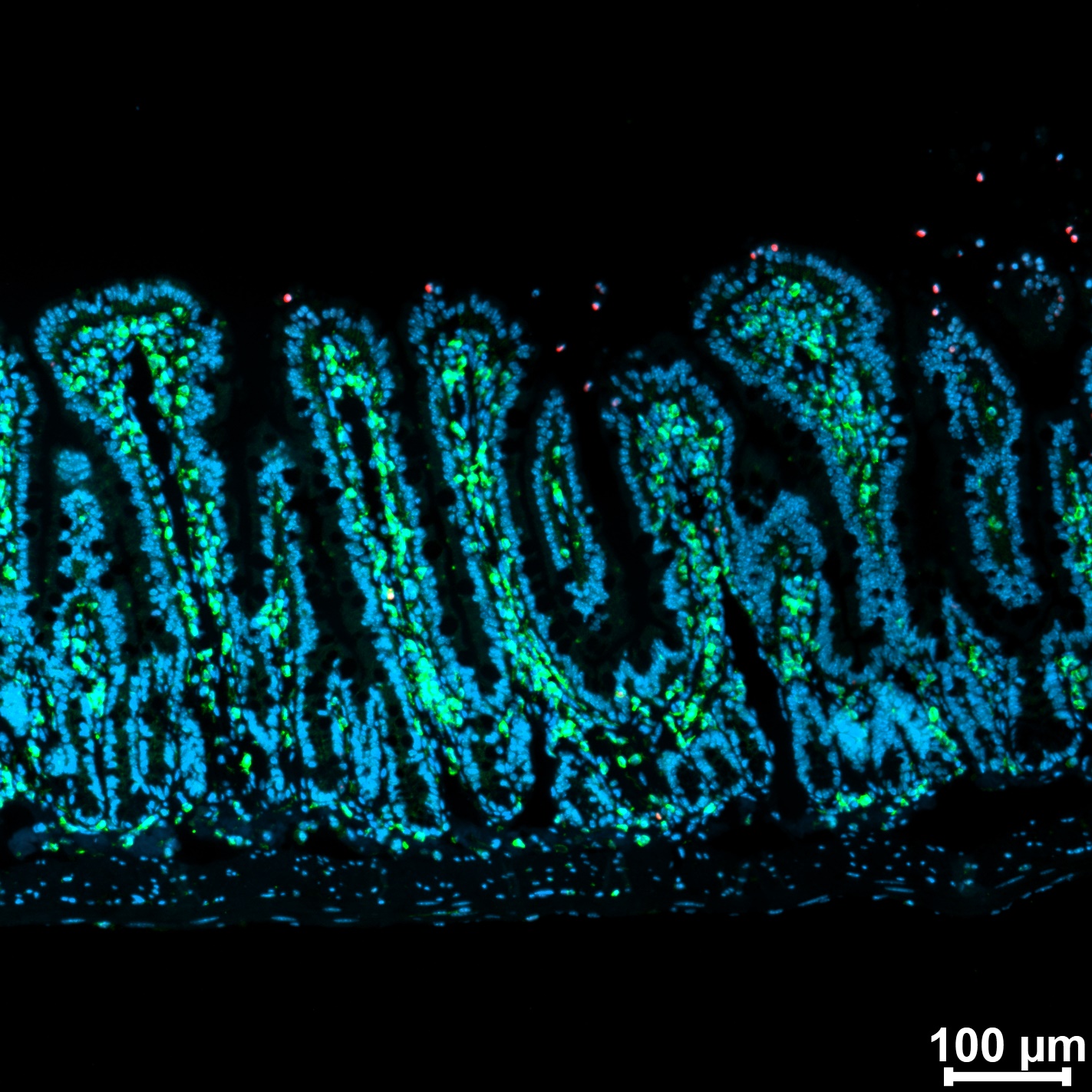

Supplement: Supplementary file 9 — Figure EV1-EV5 Source Data [file 44319_2025_441_MOESM9_ESM.zip › EV Figures/Figure EV2/EV2F/Jejunum_CD45_R64_mouse 6_KO.jpg]

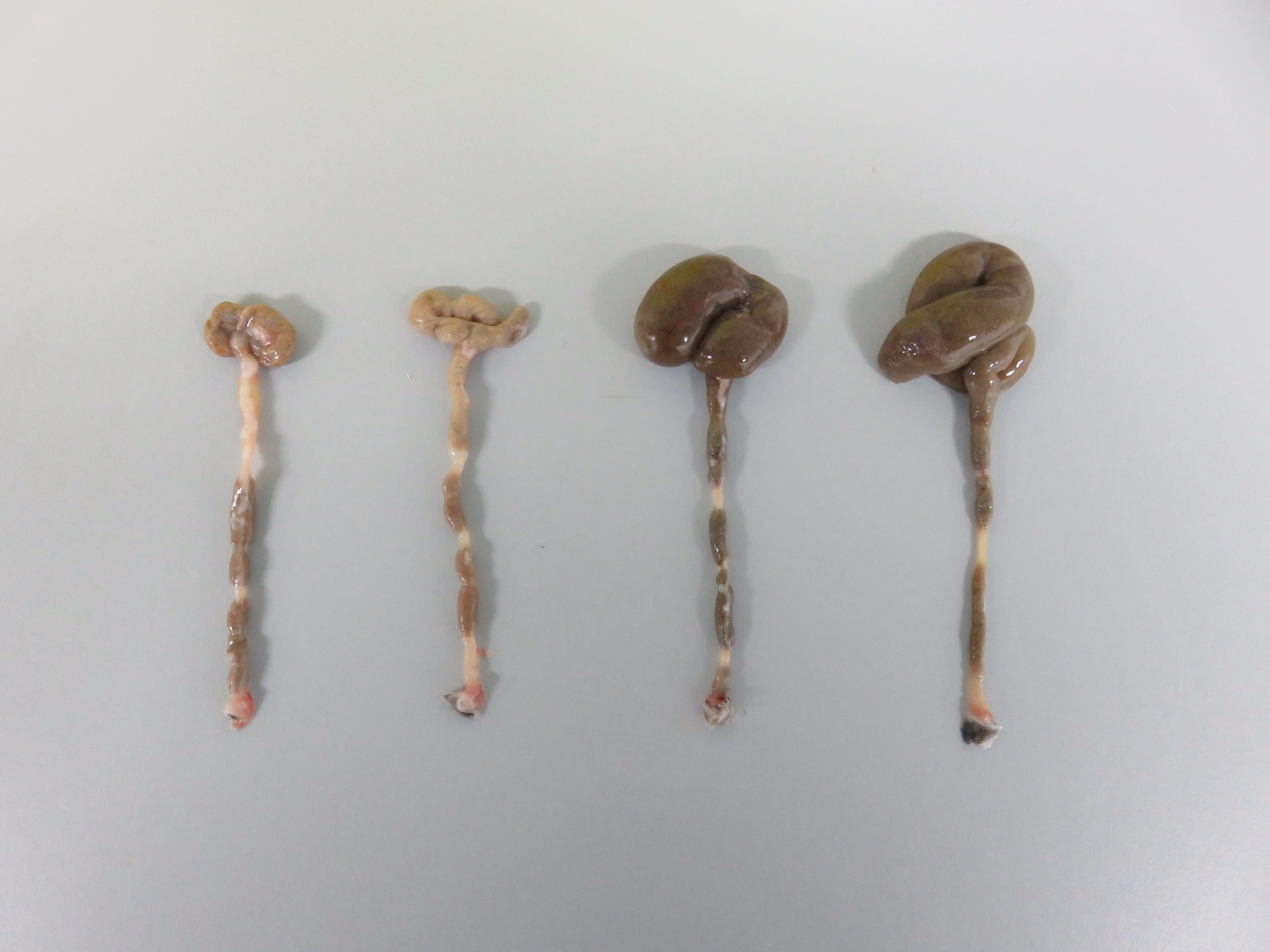

Supplement: Supplementary file 9 — Figure EV1-EV5 Source Data [file 44319_2025_441_MOESM9_ESM.zip › EV Figures/Figure EV4/EV4B/IMG_3601.JPG]

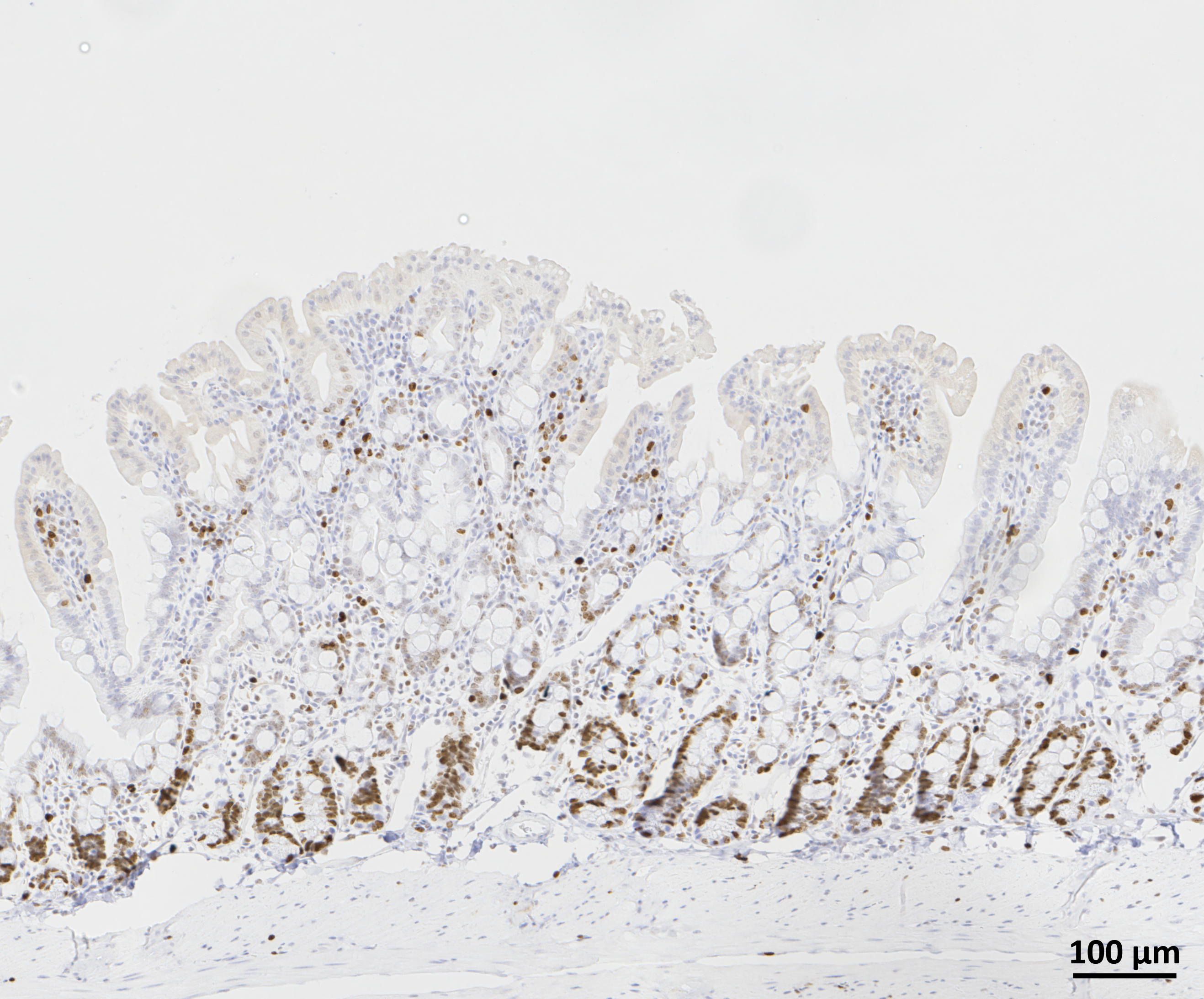

Supplement: Supplementary file 9 — Figure EV1-EV5 Source Data [file 44319_2025_441_MOESM9_ESM.zip › EV Figures/Figure EV4/EV4C/Duodenum_KO_ABX_Ki-67_R88_mouse 7.tif]

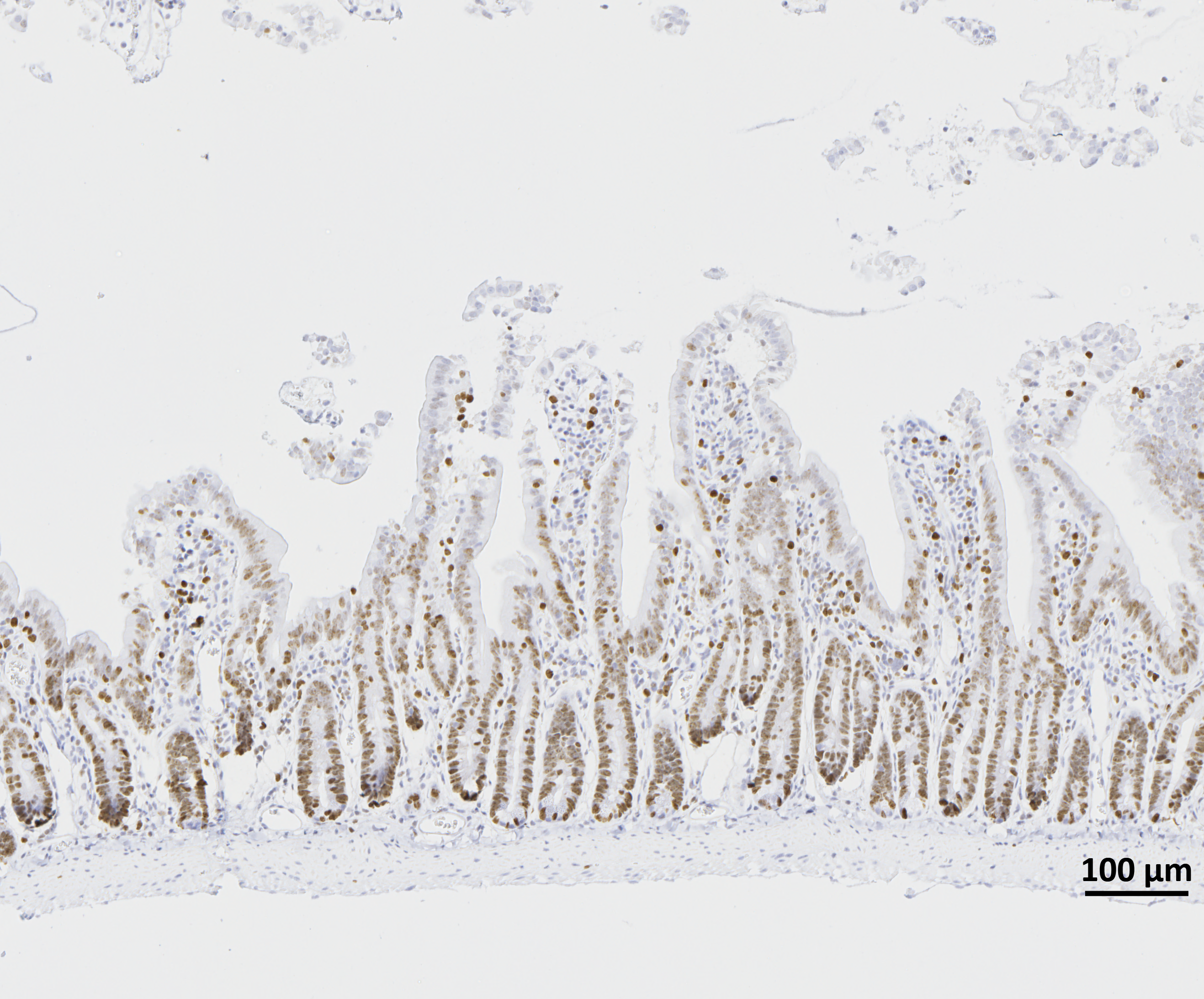

Supplement: Supplementary file 9 — Figure EV1-EV5 Source Data [file 44319_2025_441_MOESM9_ESM.zip › EV Figures/Figure EV4/EV4C/Duodenum_KO_Control_Ki-67_R88_mouse 11.tif]

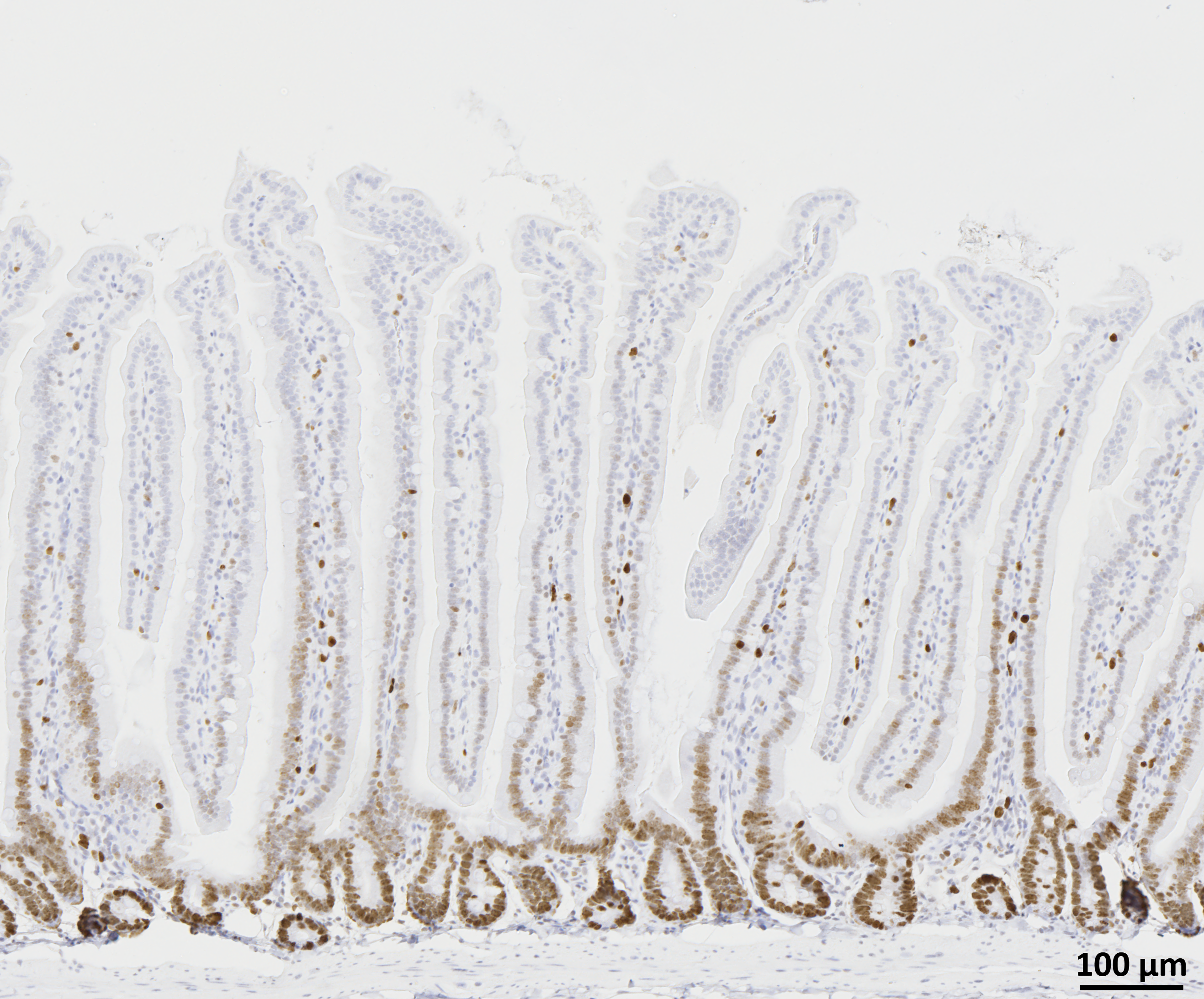

Supplement: Supplementary file 9 — Figure EV1-EV5 Source Data [file 44319_2025_441_MOESM9_ESM.zip › EV Figures/Figure EV4/EV4C/Duodenum_WT_ABX_Ki-67_R88_mouse 6.tif]

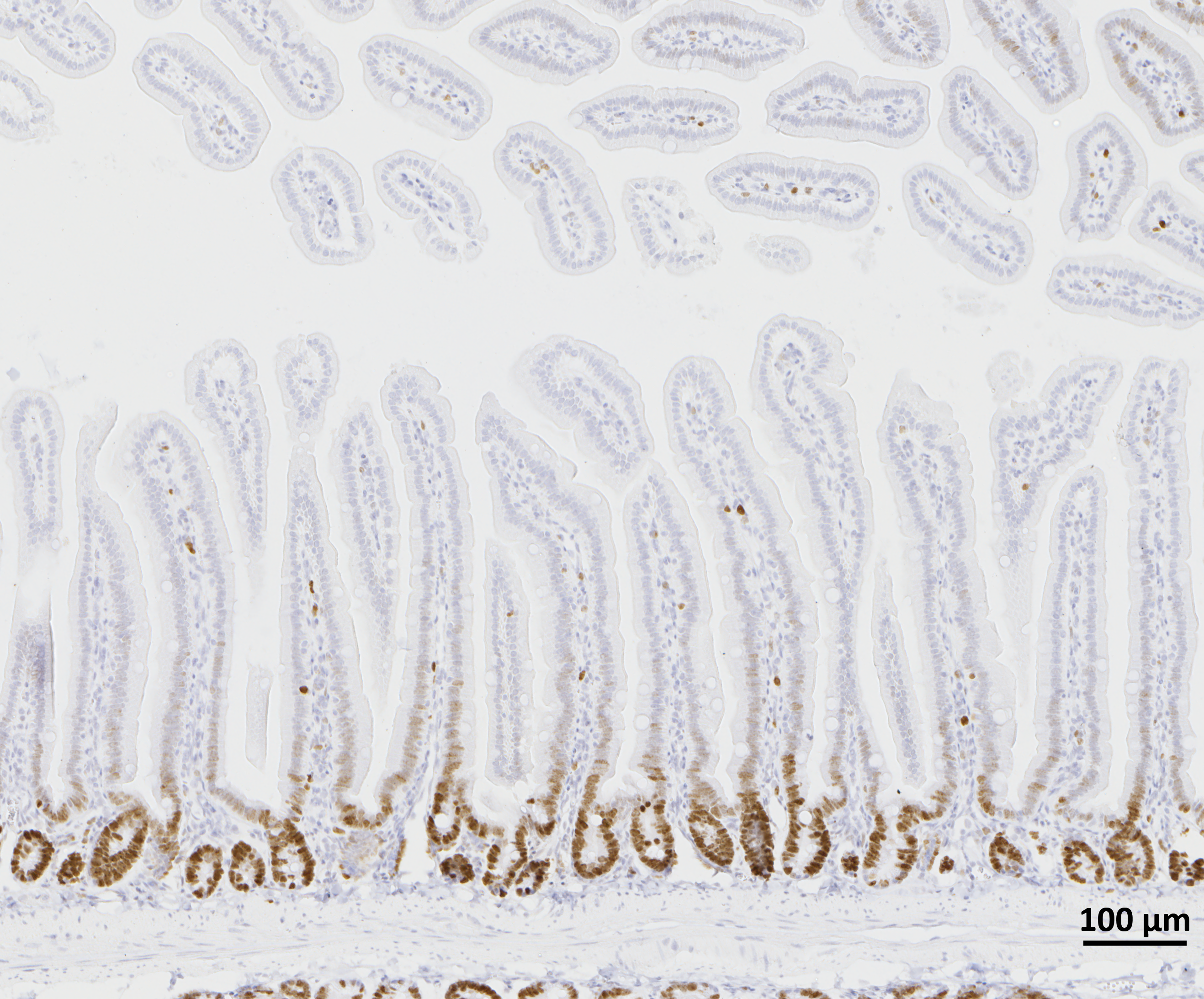

Supplement: Supplementary file 9 — Figure EV1-EV5 Source Data [file 44319_2025_441_MOESM9_ESM.zip › EV Figures/Figure EV4/EV4C/Duodenum_WT_Control_Ki-67_R88_mouse 10.tif]

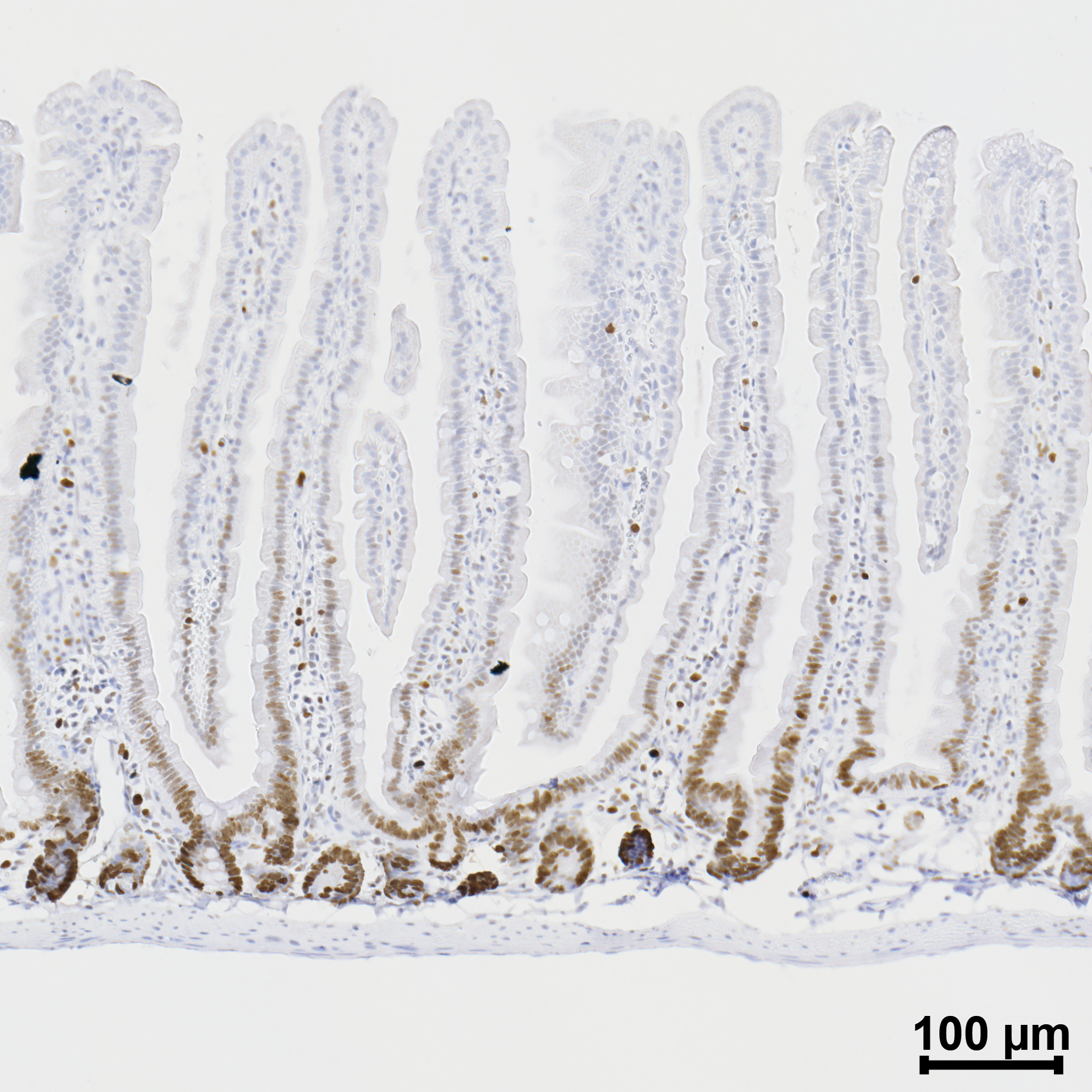

Supplement: Supplementary file 9 — Figure EV1-EV5 Source Data [file 44319_2025_441_MOESM9_ESM.zip › EV Figures/Figure EV5/EV5B/Duodenum_Ki-67_R117_mouse 1_DWT.bmp]

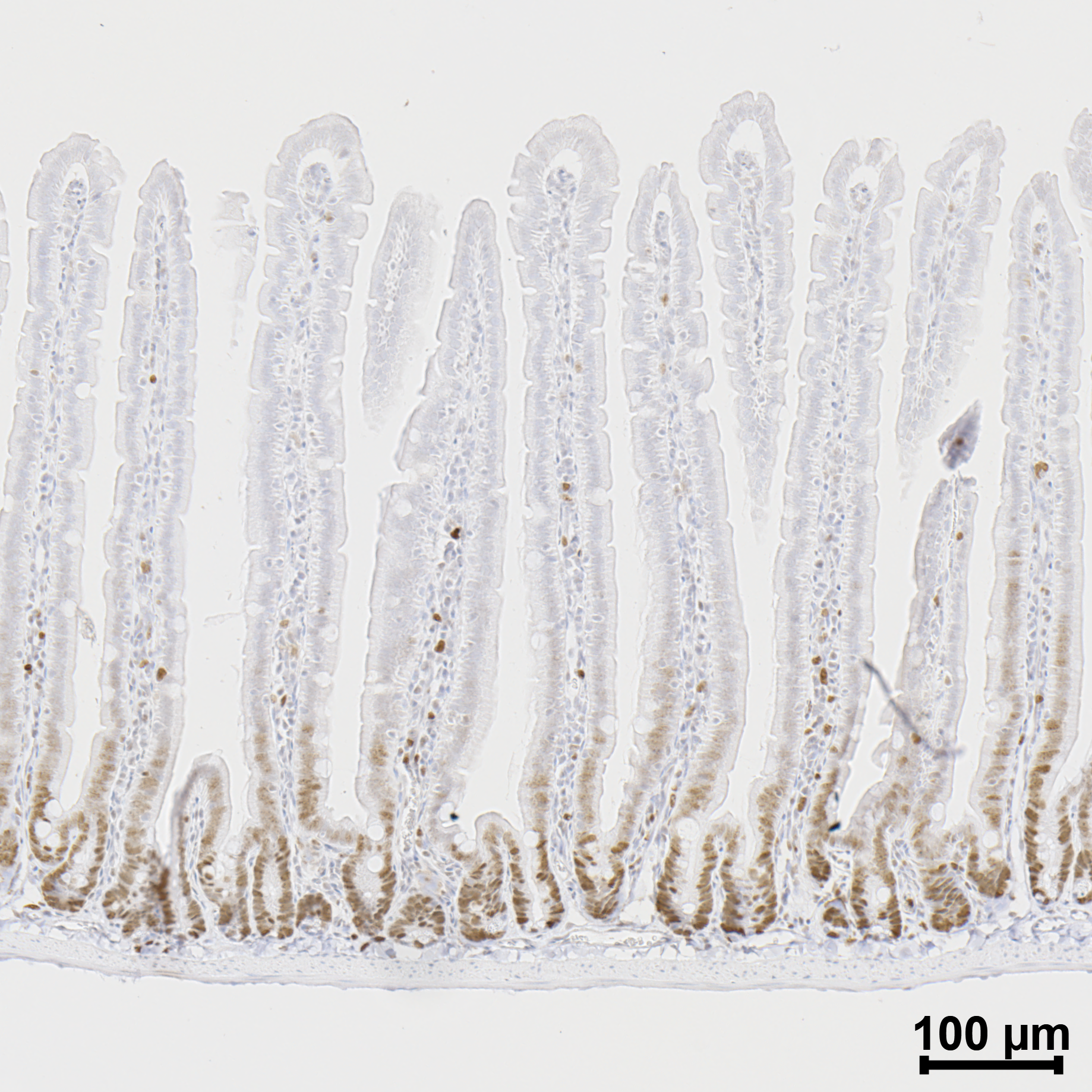

Supplement: Supplementary file 9 — Figure EV1-EV5 Source Data [file 44319_2025_441_MOESM9_ESM.zip › EV Figures/Figure EV5/EV5B/Duodenum_Ki-67_R117_mouse 3_DKO.bmp]

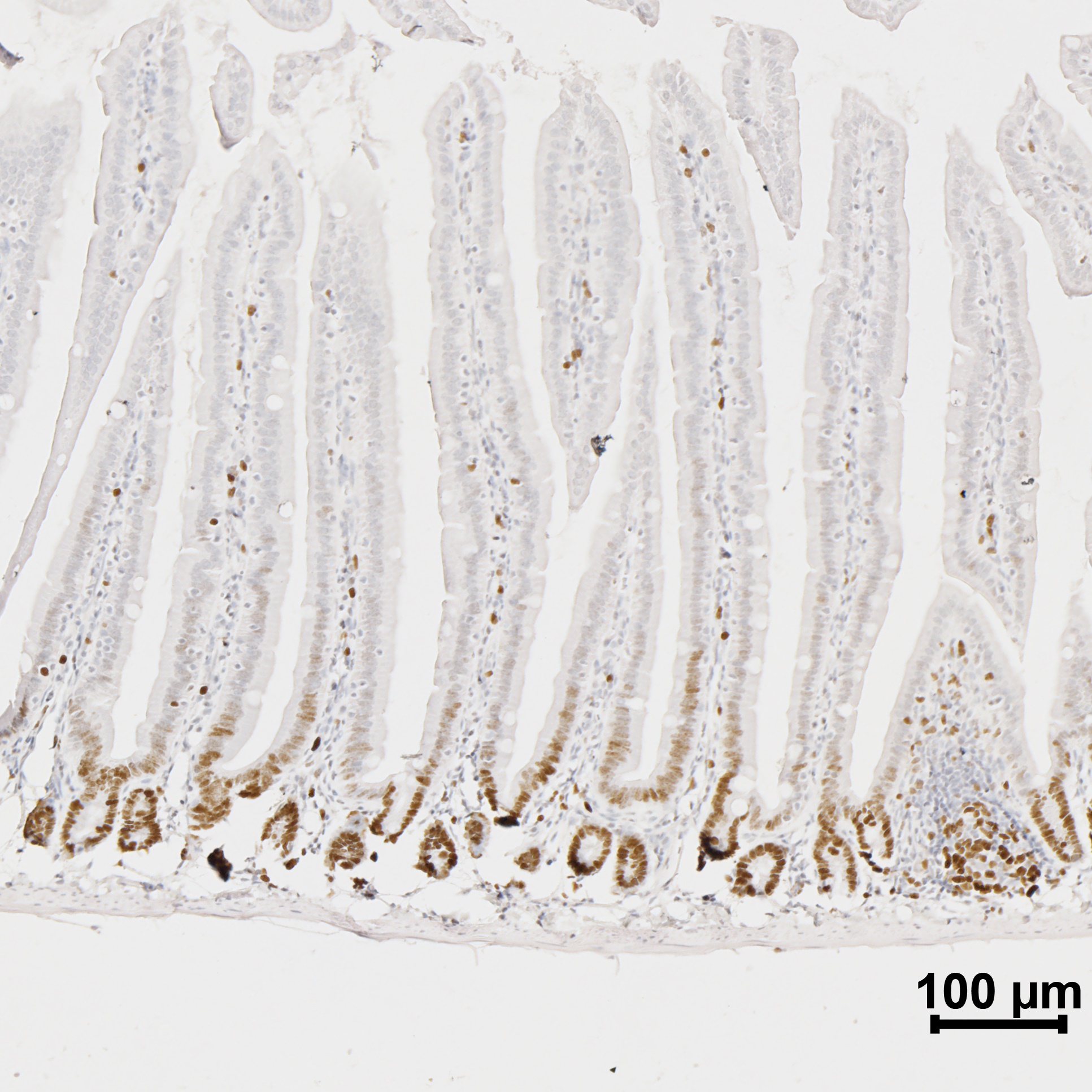

Supplement: Supplementary file 9 — Figure EV1-EV5 Source Data [file 44319_2025_441_MOESM9_ESM.zip › EV Figures/Figure EV5/EV5B/Duodenum_Ki-67_R64_mouse 3_WT.jpg]

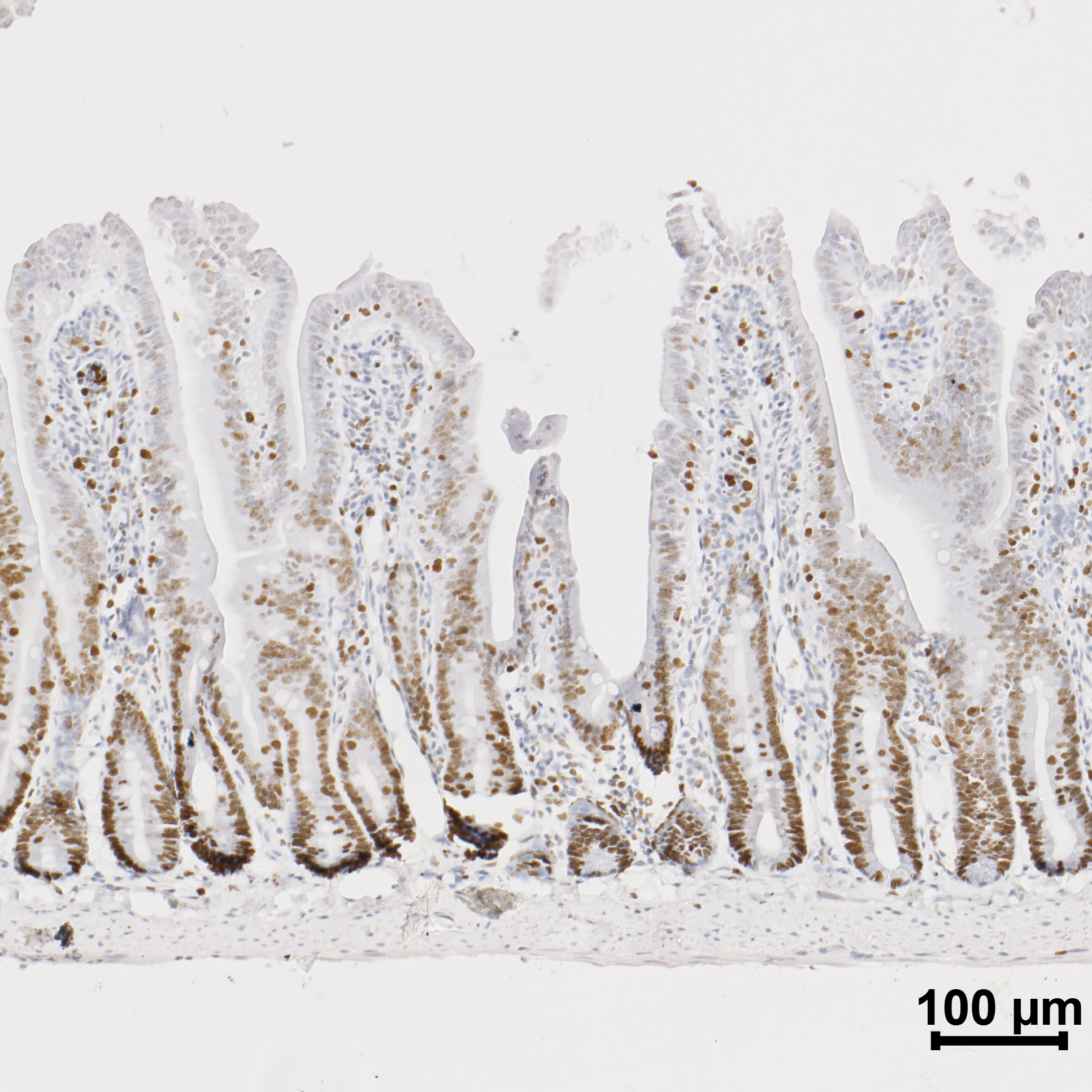

Supplement: Supplementary file 9 — Figure EV1-EV5 Source Data [file 44319_2025_441_MOESM9_ESM.zip › EV Figures/Figure EV5/EV5B/Duodenum_Ki-67_R64_mouse 8_KO.jpg]

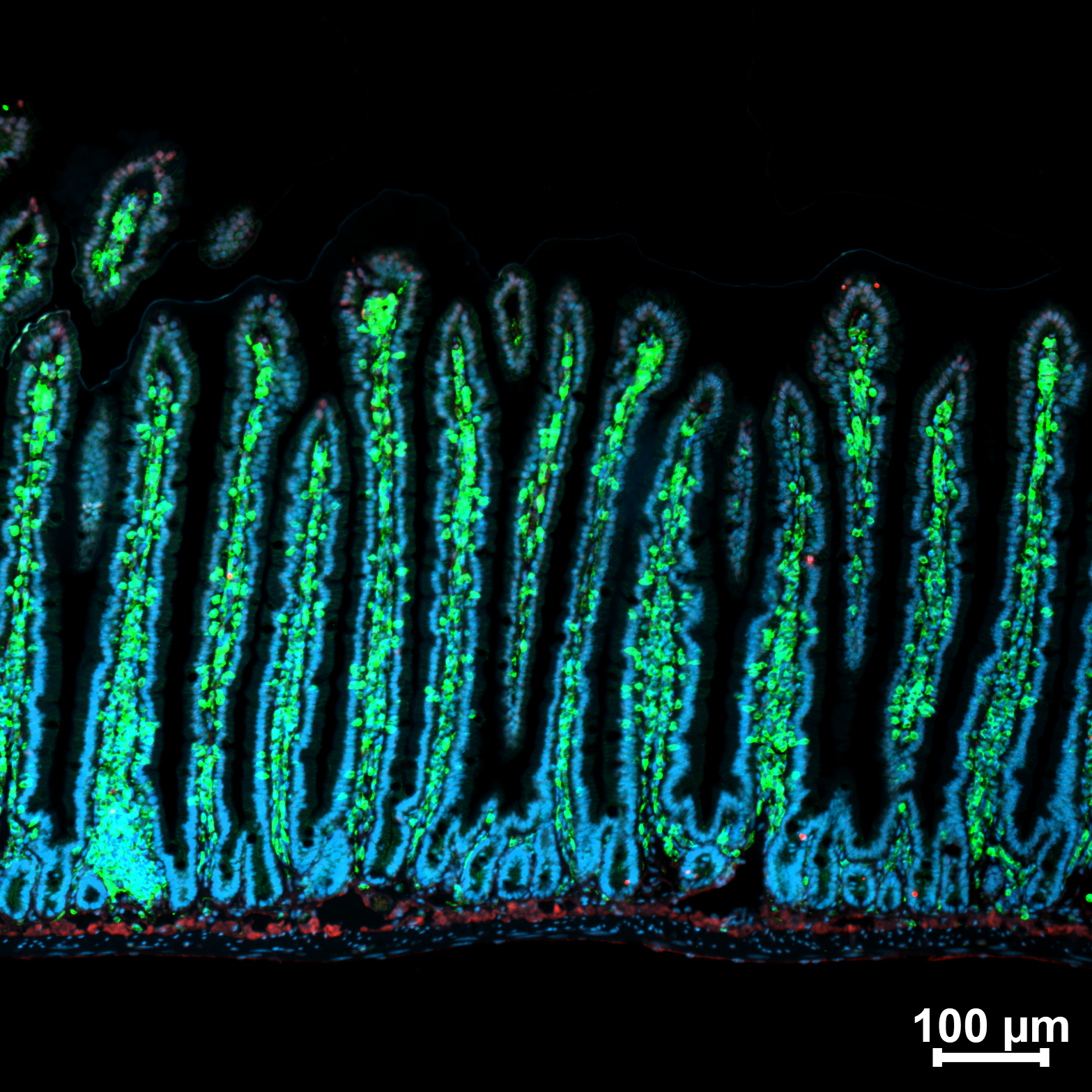

Supplement: Supplementary file 9 — Figure EV1-EV5 Source Data [file 44319_2025_441_MOESM9_ESM.zip › EV Figures/Figure EV5/EV5C/Duodenum_CD45_R117_mouse 1_DWT.bmp]

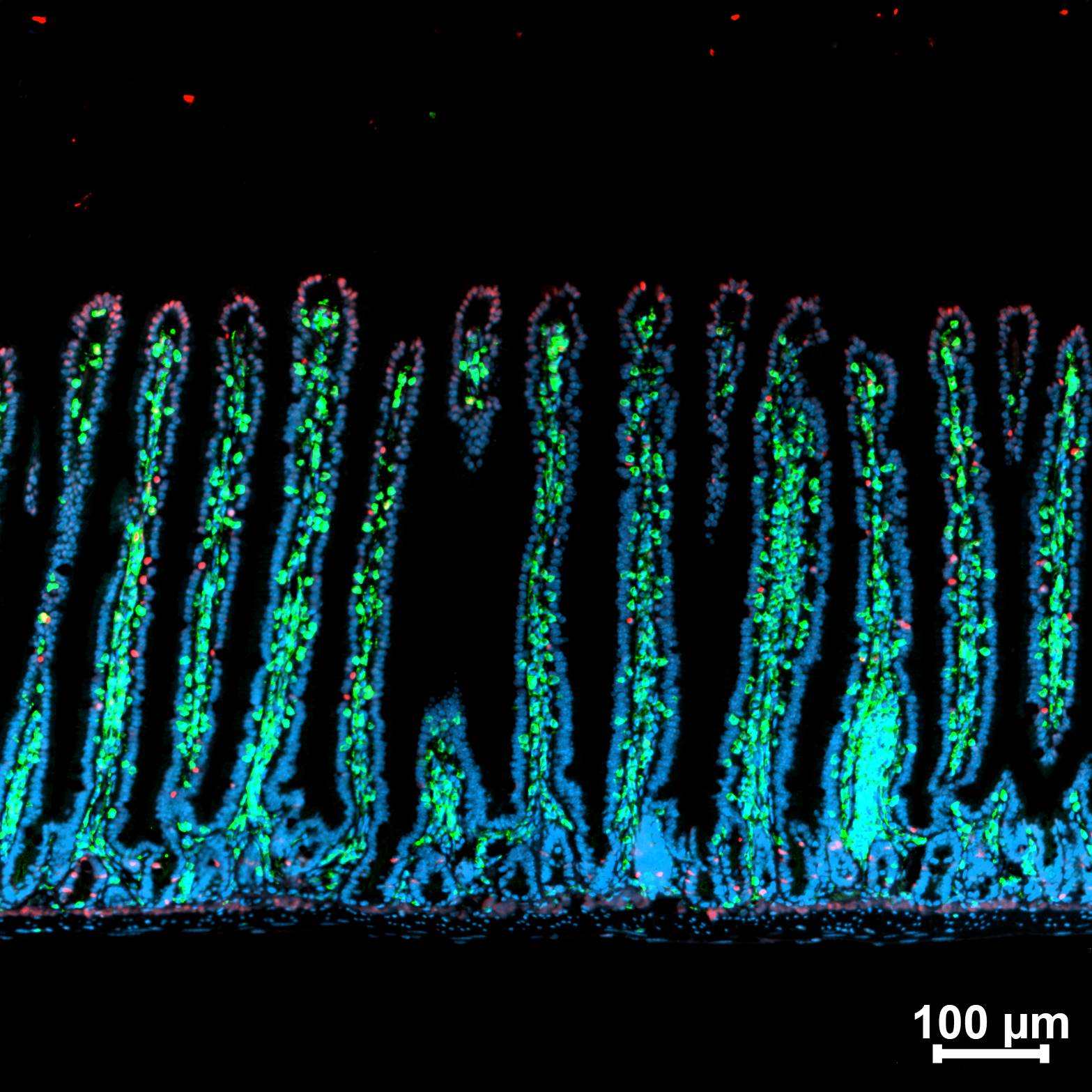

Supplement: Supplementary file 9 — Figure EV1-EV5 Source Data [file 44319_2025_441_MOESM9_ESM.zip › EV Figures/Figure EV5/EV5C/Duodenum_CD45_R117_mouse 3_DKO.bmp]

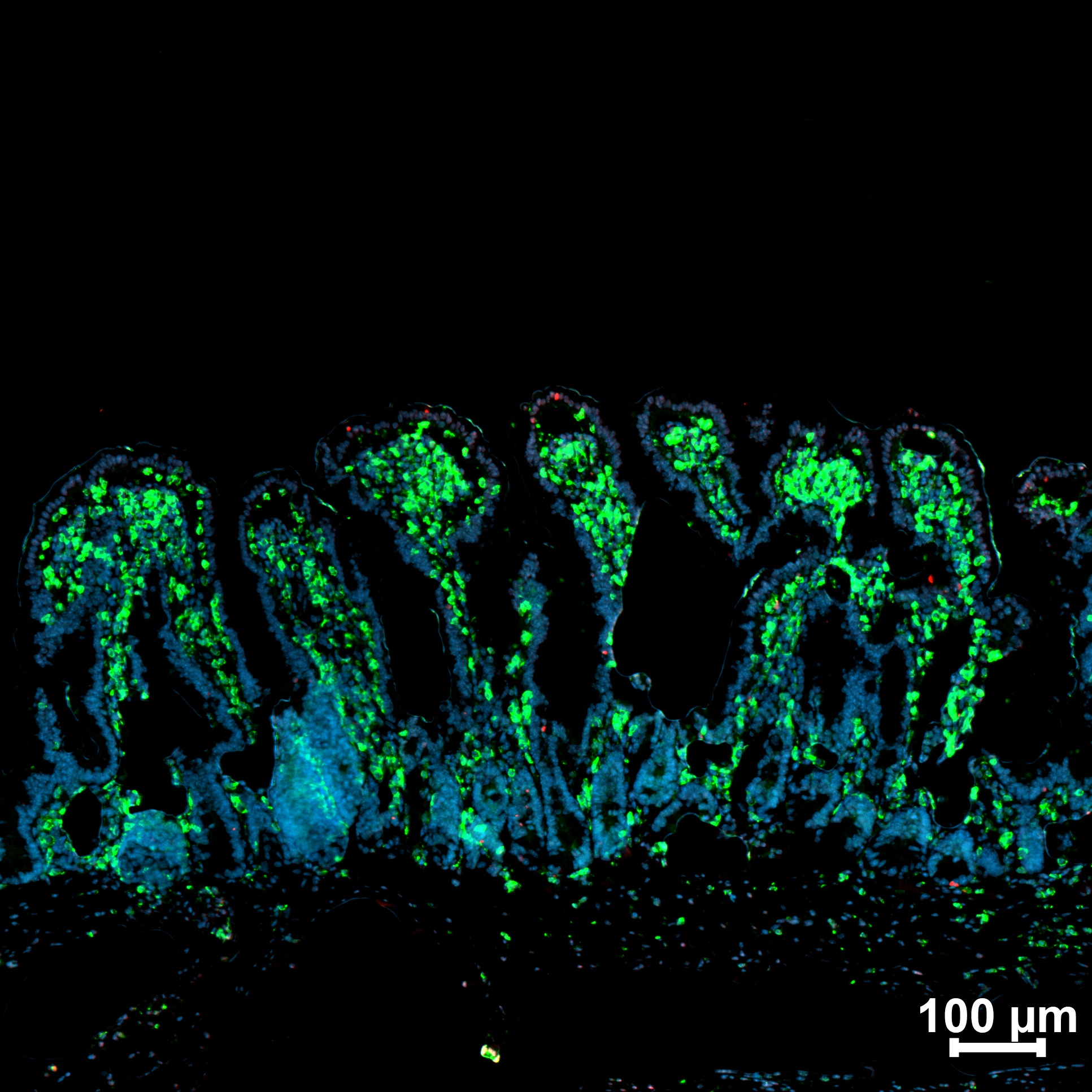

Supplement: Supplementary file 9 — Figure EV1-EV5 Source Data [file 44319_2025_441_MOESM9_ESM.zip › EV Figures/Figure EV5/EV5C/Duodenum_CD45_R64_mouse 11_KO.jpg]

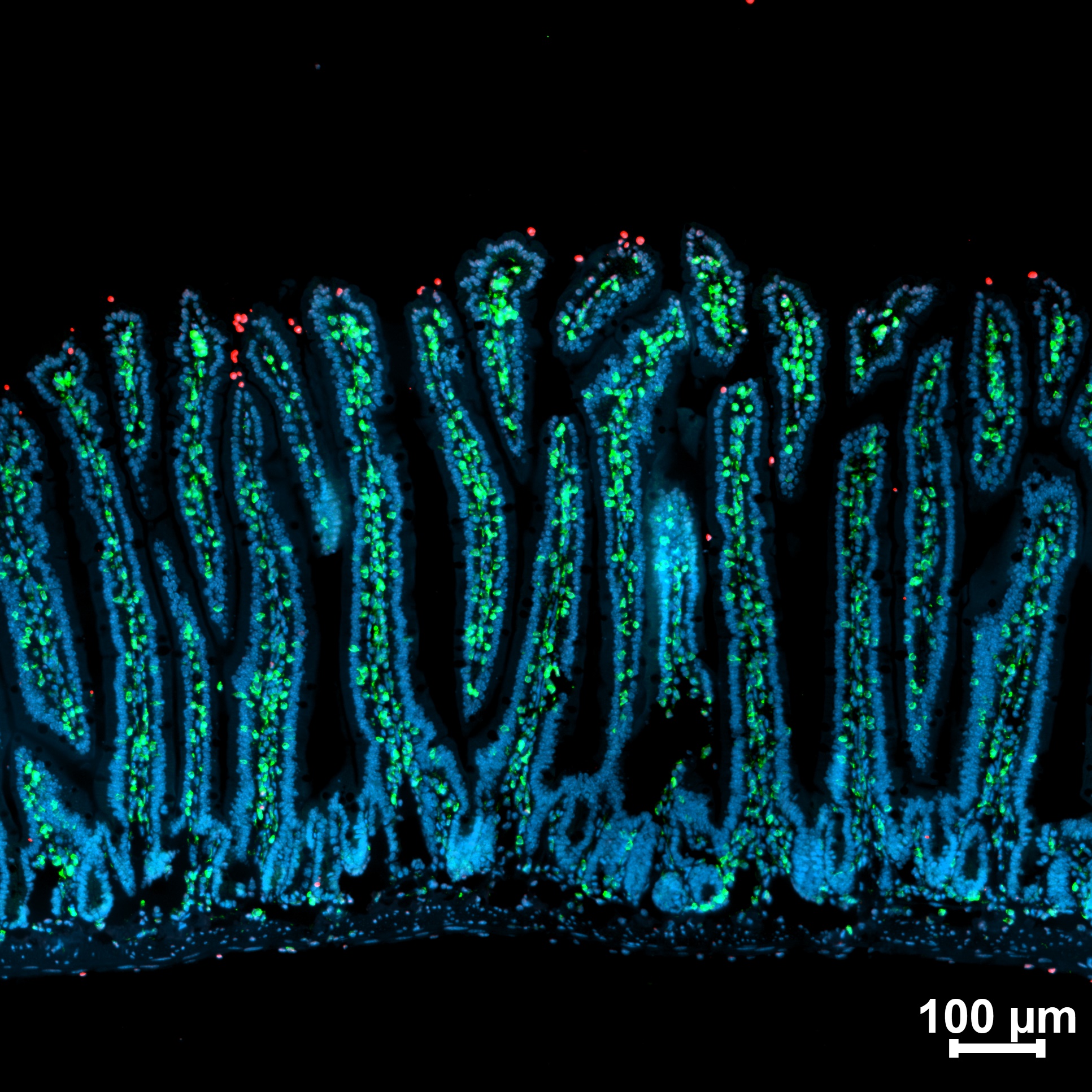

Supplement: Supplementary file 9 — Figure EV1-EV5 Source Data [file 44319_2025_441_MOESM9_ESM.zip › EV Figures/Figure EV5/EV5C/Duodenum_CD45_R64_mouse 3_WT.jpg]
