# Supplementary material for: RIPK1 ablation in T cells results in spontaneous enteropathy and TNF-driven villus atrophy
Source: EMBO Rep. 2025 Apr 30;26(10):2654–82. doi: 10.1038/s44319-025-00441-5 (PMC12117051; doi:10.1038/s44319-025-00441-5)
Supplement: Supplementary file 10 — Expanded View Figures [file 44319_2025_441_MOESM10_ESM.pdf]

Expanded View Figures

Figure EV1. Selective deletion of *Ripk1* in conventional T cells results in small intestinal inflammation and elongation.

(**A**) Distribution of pups with indicated genotypes from crosses between *Ripk1*$^{FL/FL}$; *Cd4-Cre*$^{+/+}$ and *Ripk1*$^{FL/+}$; *Cd4-Cre*$^{Tg/+}$ parents. Bars represent the observed percentage of mice with each genotype within a total population of $n = 205$ mice. The expected percentage is denoted by the horizontal dotted line, and the *p*-value resulting from a chi-square test are indicated. (**B**) Absolute numbers of CD4^{+} and CD8^{+} T cells in the mesenteric lymph nodes (mLN) of *Ripk1*$^{\Delta CD4}$ mice and *Ripk1*$^{FL/FL}$ littermates, measured by flow cytometry. (**C**) Representative image of the full colon and caecum of an aged *Ripk1*$^{\Delta CD4}$ mouse and *Ripk1*$^{FL/FL}$ littermate. (**D**) Quantification of the absolute length of the colon of young *Ripk1*$^{\Delta CD4}$ mice ($n = 22$) and *Ripk1*$^{FL/FL}$ littermates ($n = 22$) and aged *Ripk1*$^{\Delta CD4}$ mice ($n = 25$) and *Ripk1*$^{FL/FL}$ littermates ($n = 22$). (**E**) Representative images of an aged homozygous *Ripk1*$^{\Delta CD4}$ mouse, heterozygous *Ripk1*$^{Fl/+}$;*Cd4-Cre*$^{Tg/+}$ mouse and *Ripk1*$^{FL/FL}$ littermate with respective spleen, mLN and small intestine (SI). (**F**) Representative images of an aged *Ripk1*^{K45A} mouse and *Ripk1*$^{+/+}$ littermate with respective spleen, mLN and small intestine (SI). (**G**) Quantification of the absolute SI length of young *Ripk1*^{K45A} mice ($n = 4$) and *Ripk1*$^{+/+}$ littermates ($n = 3$) and aged *Ripk1*^{K45A} mice ($n = 7$) and *Ripk1*$^{+/+}$ littermates ($n = 6$). (**H**) Tissue concentrations of IL-4, IL-5, IL-6, IL-10 IL-13, and TGF-β in the SI of young *Ripk1*$^{\Delta CD4}$ mice ($n = 13$) and *Ripk1*$^{FL/FL}$ littermates ($n = 11$), and aged *Ripk1*$^{\Delta CD4}$ mice ($n = 17$) and *Ripk1*$^{FL/FL}$ littermates ($n = 16$), were measured using multiplex assays (Meso Scale Discovery). Data are shown as mean ± SEM, with means represented by bars or horizontal lines and each dot representing an individual mouse. Data are representative of at least three independent repeats (**C**, **F**), or are combined from two independent repeats (**B**). Statistical significance was calculated in Graphpad Prism by (**A**) chi-square test, (**B**) Fisher's LSD two-way ANOVA on Log_2-transformed data CD4^{+}: $p = 0.0006$, CD8β^{+}: $p < 0.0001$, (**D**, **G**) Fisher's LSD two-way ANOVA on absolute values, or (**H**) two-sided unpaired T test with Welch's correction. ns non-significant, na not applicable, ***$p < 0.001$, ****$p < 0.0001$. Young mice: 8–12 weeks old. Aged mice: >6 months old. Source data are available online for this figure.

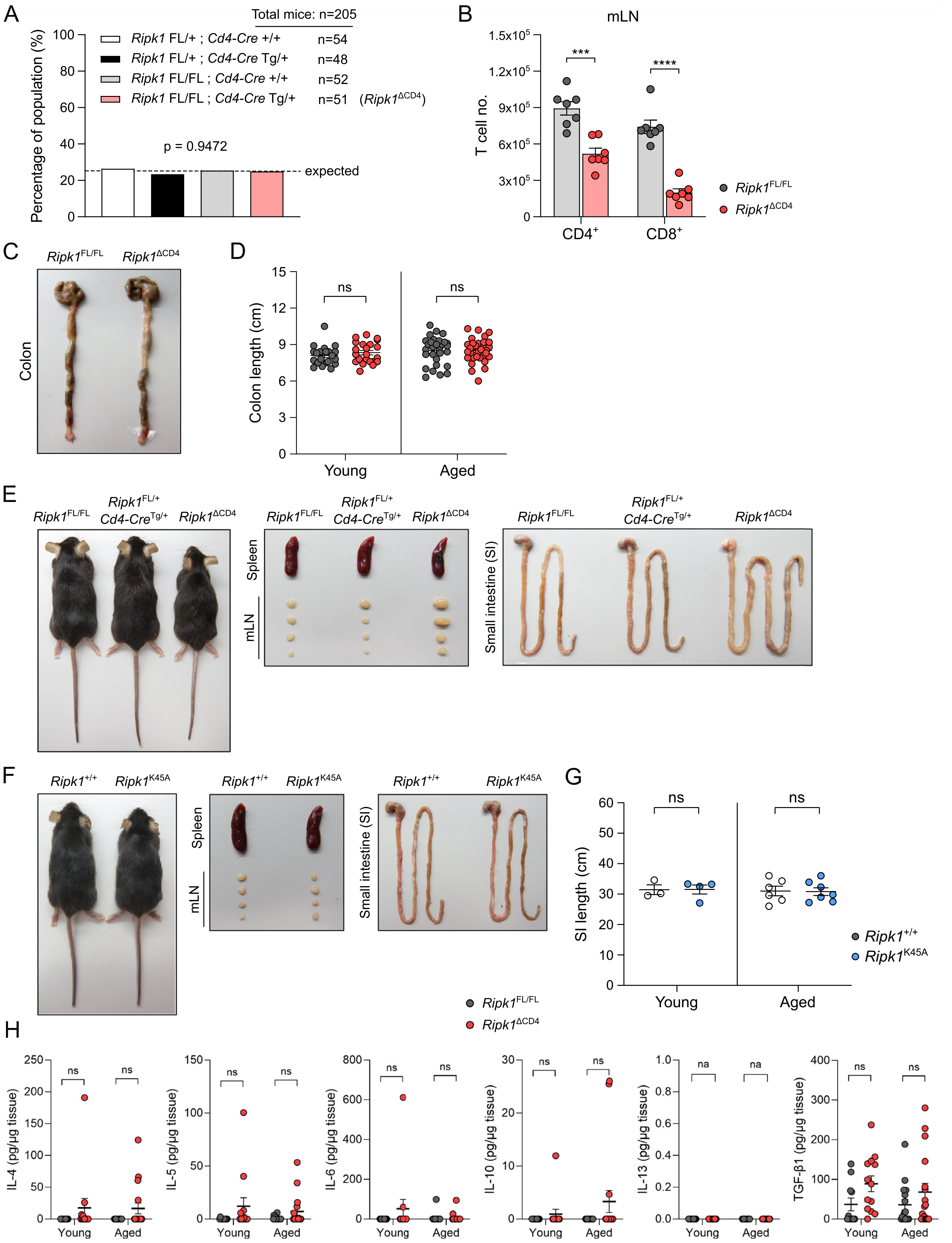
A
Total mice: n=205
Ripk1 FL/+ ; Cd4-Cre +/+ n=54
Ripk1 FL/+ ; Cd4-Cre Tg/+ n=48
Ripk1 FL/FL ; Cd4-Cre +/+ n=52
Ripk1 FL/FL ; Cd4-Cre Tg/+ n=51 (Ripk1ΔCD4)
Percentage of population (%)
p = 0.9472
expected
B
mLN
T cell no.
***
****
CD4+
CD8+
Ripk1FL/FL
Ripk1ΔCD4
C
Ripk1FL/FL
Ripk1ΔCD4
Colon
D
Colon length (cm)
ns
ns
Young
Aged
E
Ripk1FL/FL
Ripk1FL/+ Cd4-CreTg/+
Ripk1ΔCD4
Spleen
mLN
Small intestine (SI)
F
Ripk1+/+
Ripk1K45A
Spleen
mLN
Small intestine (SI)
G
SI length (cm)
ns
ns
Young
Aged
Ripk1+/+
Ripk1K45A
H
Ripk1FL/FL
Ripk1ΔCD4
IL-4 (pg/µg tissue)
IL-5 (pg/µg tissue)
IL-6 (pg/µg tissue)
IL-10 (pg/µg tissue)
IL-13 (pg/µg tissue)
TGF-β1 (pg/µg tissue)
ns
na
Young
Aged

 © The Author(s)

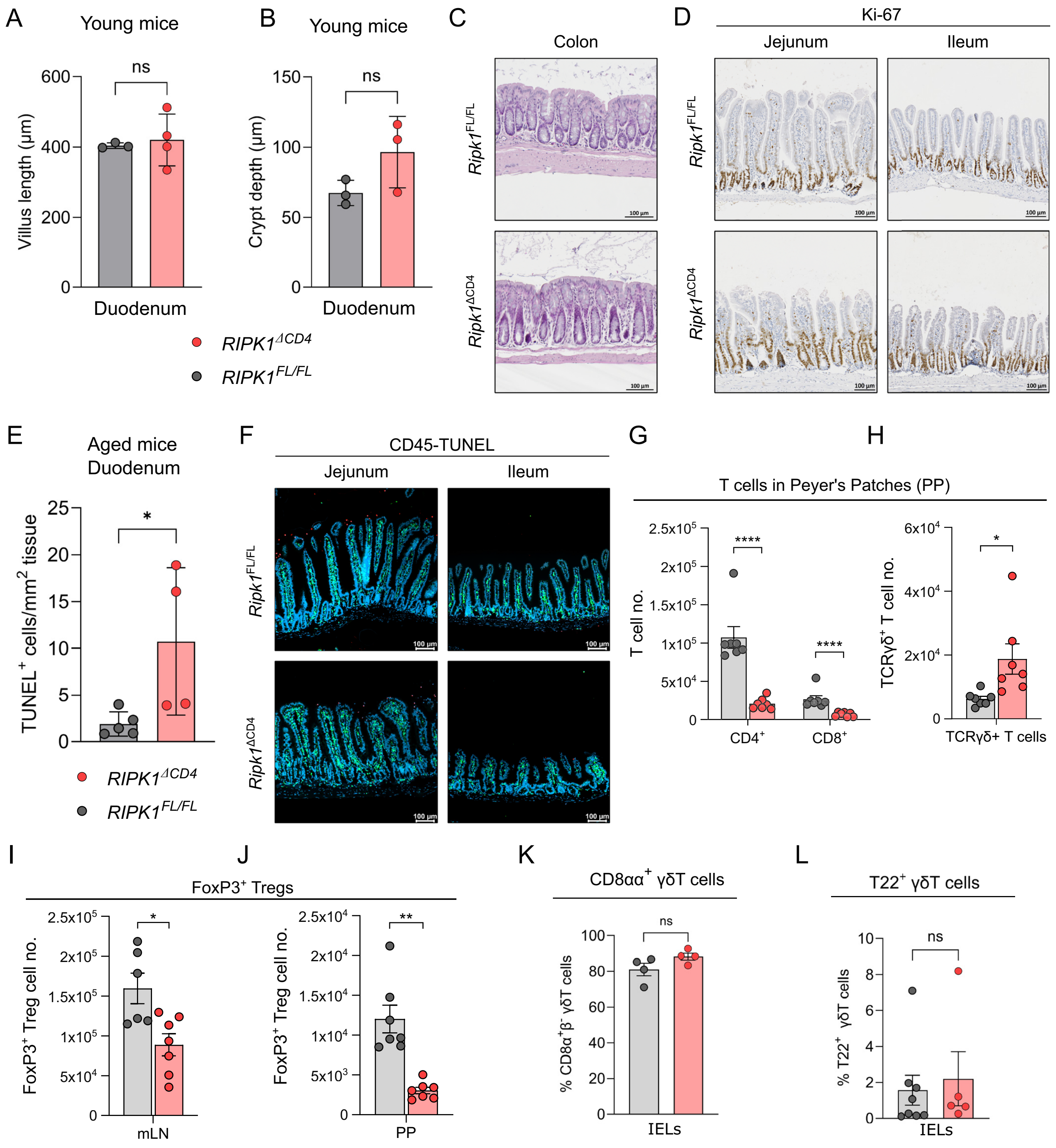
A
Young mice
Villus length (µm)
Duodenum
ns
B
Young mice
Crypt depth (µm)
Duodenum
ns
RIPK1ΔCD4
RIPK1FL/FL
C
Colon
Ripk1FL/FL
Ripk1ΔCD4
D
Ki-67
Jejunum
Ileum
E
Aged mice
Duodenum
TUNEL+ cells/mm2 tissue
*
F
CD45-TUNEL
Jejunum
Ileum
G
H
T cells in Peyer's Patches (PP)
T cell no.
CD4+
CD8+
****
TCRγδ+ T cell no.
TCRγδ+ T cells
I
J
FoxP3+ Tregs
FoxP3+ Treg cell no.
mLN
PP
**
K
CD8αα+ γδT cells
% CD8α+β- γδT cells
IELs
L
T22+ γδT cells
% T22+ γδT cells

Figure EV2. The duodenum of *Ripk1*$^{\Delta CD4}$ mice displays villus atrophy, crypt hyperplasia, and immune cell infiltration.

(**A**, **B**) Quantification of (**A**) the average villus length and (**B**) the average crypt depth in the duodenum of young *Ripk1*$^{\Delta CD4}$ and *Ripk1*$^{FL/FL}$ littermates ($n = 3$). (**C**) H&E staining on sections of the colon of an aged *Ripk1*$^{\Delta CD4}$ mouse and *Ripk1*$^{FL/FL}$ littermate. (**D**) Ki-67 staining on sections of the jejunum and ileum of aged *Ripk1*$^{\Delta CD4}$ mice and *Ripk1*$^{FL/FL}$ littermates. (**E**) Quantification of TUNEL staining in the duodenum of aged *Ripk1*$^{\Delta CD4}$ mice ($n = 4$) and *Ripk1*$^{FL/FL}$ littermates ($n = 5$), representing TUNEL-positive cells/mm^2 tissue area. (**F**) CD45-TUNEL staining on sections of the jejunum and ileum of aged *Ripk1*$^{\Delta CD4}$ mice ($n = 4$) and *Ripk1*$^{FL/FL}$ littermates ($n = 5$). (**C**, **D**, **F**) Images are representative of *Ripk1*$^{\Delta CD4}$ mice ($n = 4$) and *Ripk1*$^{FL/FL}$ littermates ($n = 5$). (**G**) Absolute numbers of CD4$^+$ and CD8$^+$ T cells in the Peyer's patches (PP) of young *Ripk1*$^{\Delta CD4}$ mice and *Ripk1*$^{FL/FL}$ littermates, measured by flow cytometry. (**H**) Absolute numbers of TCRγδ$^+$ T cells in the PP of young *Ripk1*$^{\Delta CD4}$ mice and *Ripk1*$^{FL/FL}$ littermates, measured by flow cytometry. Data are obtained from $n = 7$ mice per group (**G**, **H**). (**I**, **J**) Absolute numbers of FoxP3$^+$ Tregs in the mLN (**I**) and PP (**J**) of young *Ripk1*$^{\Delta CD4}$ mice and *Ripk1*$^{FL/FL}$ littermates, measured by flow cytometry. (**I**, **J**) Data are obtained from *Ripk1*$^{\Delta CD4}$ mice ($n = 6$) and *Ripk1*$^{FL/FL}$ littermates ($n = 7$). (**K**) Proportions of TCRγδ$^+$ IELs expressing the CD8αα receptor in young *Ripk1*$^{\Delta CD4}$ mice ($n = 4$) and *Ripk1*$^{FL/FL}$ littermates ($n = 4$), measured by flow cytometry. (**L**) Proportions of TCRγδ$^+$ IELs expressing the T22 TCR of young *Ripk1*$^{\Delta CD4}$ mice ($n = 5$) and *Ripk1*$^{FL/FL}$ littermates ($n = 8$), measured by flow cytometry. (**G**, **L**) Data are representative of at least two independent repeats. Data are shown as mean ± SEM, with means being represented by bars or horizontal lines and each dot representing an individual mouse. Statistical significance was calculated in Graphpad Prism by Fisher's LSD two-way ANOVA on Log_2-transformed data (**G**) CD4$^+$ and CD8$^+$ T cells: $p < 0.0001$, Mann-Whitney test (**A**) $p = 0.6286$, (**B**) $p = 0.200$, and (**E**) $p = 0.0317$, or two-sided unpaired T test with Welch's correction (**H**) $p = 0.039$, (**I**) $p = 0.0141$, (**J**) $p = 0.0018$, (**K**) $p = 0.1345$, (**L**) $p = 0.7231$. ns = non-significant, *$p < 0.05$, **$p < 0.01$, ****$p < 0.0001$. Young mice: 8–12 weeks old. Aged mice: >6 months old. Source data are available online for this figure.

 © The Author(s)

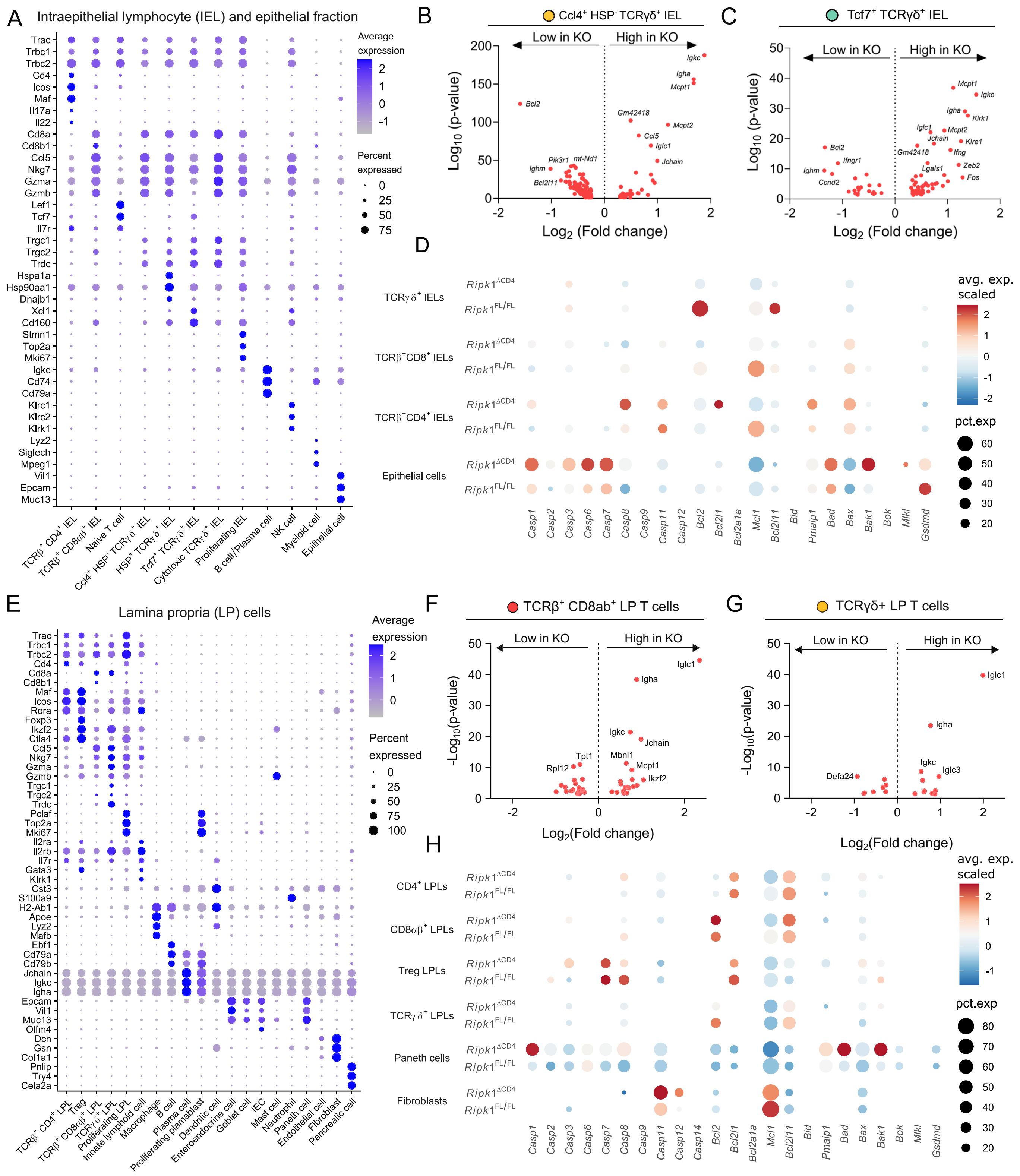
A
Intraepithelial lymphocyte (IEL) and epithelial fraction
Average expression
Percent expressed
TCRβ+ CD4+ IEL
TCRβ+ CD8αβ+ IEL
Naive T cell
Ccl4+ HSP- TCRγδ+ IEL
HSP+ TCRγδ+ IEL
Tcf7+ TCRγδ+ IEL
Cytotoxic TCRγδ+ IEL
Proliferating IEL
B cell/Plasma cell
NK cell
Myeloid cell
Epithelial cell
B
Ccl4+ HSP- TCRγδ+ IEL
Low in KO
High in KO
Log10 (p-value)
Log2 (Fold change)
C
Tcf7+ TCRγδ+ IEL
Low in KO
High in KO
Log10 (p-value)
Log2 (Fold change)
D
TCRγδ+ IELs
TCRβ+CD8+ IELs
TCRβ+CD4+ IELs
Epithelial cells
Ripk1ΔCD4
Ripk1FL/FL
avg. exp. scaled
pct.exp
E
Lamina propria (LP) cells
Average expression
Percent expressed
TCRβ+ CD4+ LPL
Treg
TCRβ+ CD8αβ+ LPL
TCRγδ+ LPL
Proliferating LPL
Innate lymphoid cell
Macrophage
B cell
Plasma cell
Proliferating plamablast
Dendritic cell
Enteroendocrine cell
Goblet cell
IEC
Mast cell
Neutrophil
Paneth cell
Endothelial cell
Fibroblast
Pancreatic cell
F
TCRβ+ CD8ab+ LP T cells
Low in KO
High in KO
-Log10(p-value)
Log2(Fold change)
G
TCRγδ+ LP T cells
Low in KO
High in KO
-Log10(p-value)
Log2(Fold change)
H
CD4+ LPLs
CD8αβ+ LPLs
Treg LPLs
TCRγδ+ LPLs
Paneth cells
Fibroblasts
Ripk1ΔCD4
Ripk1FL/FL
avg. exp. scaled
pct.exp

Figure EV3. *Ripk1*$^{\Delta CD4}$ mice display altered expression of cell death genes in intraepithelial and lamina propria cells.

(**A**) Cells were isolated from the epithelial layer (IEL) of young *Ripk1*$^{\Delta CD4}$ *and Ripk1*$^{FL/FL}$ littermates and subjected to single-cell RNA sequencing (scRNA-seq). (**B**, **C**) Volcano plots representing significant differentially expressed (DE) genes in the clusters of (B) *Ccl4*$^{+}$ HSP^{-} TCRγδ^{+} IELs, and (C) *Tcf7*$^{+}$ TCRγδ^{+} IELs of *Ripk1*$^{\Delta CD4}$ mice compared to *Ripk1*$^{FL/FL}$ littermates. Significance ($-$Log$_{10}$ of the *p*-value) is indicated on the y-axis, and Log$_2$ of the fold change in gene expression is indicated on the x-axis. (**D**) Dot plot displaying gene expression of Caspases, intrinsic apoptosis genes, and pore-forming proteins in T cell populations and epithelial cells in the IEL fraction. Size of dots represents the fraction of cells expressing a particular marker, and color intensity indicates mean-normalized scaled expression levels. Cells were isolated from the lamina propria (LP) of young *Ripk1*$^{\Delta CD4}$ *and Ripk1*$^{FL/FL}$ littermates and subjected to single-cell RNA sequencing (scRNA-seq). (**E**) Dot plot displaying expression of marker genes per cluster used for cluster annotation. The size of dots represents the fraction of cells expressing a particular marker and color intensity indicates mean-normalized scaled expression levels. (**F**, **G**) Volcano plots representing significant differentially expressed (DE) genes in the clusters of TCRβ^{+}CD8^{+} LP T cells (**B**) and TCRγδ^{+} LP T cells (**C**) of *Ripk1*$^{\Delta CD4}$ mice compared to *Ripk1*$^{FL/FL}$ littermates. Significance ($-$Log$_{10}$ of the *p*-value) is indicated on the y-axis, and Log$_2$ of the fold change in gene expression is indicated on the x-axis. (**H**) Dot plot displaying gene expression of Caspases, intrinsic apoptosis genes, and pore-forming proteins in T cell populations, Paneth cells, and Fibroblasts in the LP fraction. Size of dots represents the fraction of cells expressing a particular marker, and color intensity indicates mean-normalized scaled expression levels. Data were obtained from $n = 3$ mice per group (**A**–**H**). (**B**, **C**, **F**, **G**) Wilcox test was used to determine DE genes and *p*-values were adjusted using Bonferroni correction. Young mice: 8–12 weeks old. IEL intraepithelial lymphocytes, LP lamina propria. Source data are available online for this figure.

 © The Author(s)

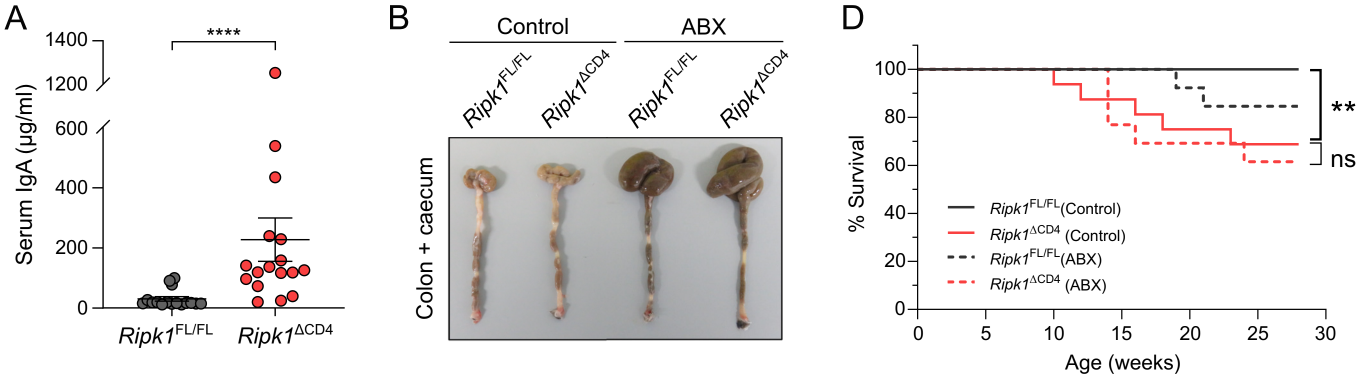

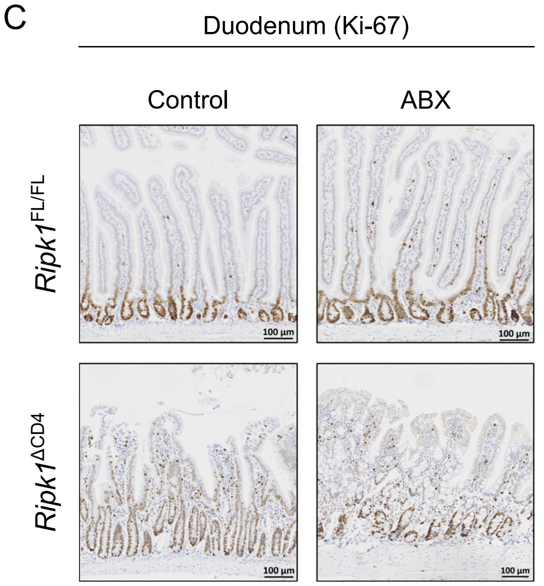

E

IELs (Fig. 6J)

Absolute number ($\times 10^5$) (±SEM)		TCRβ+ CD4+	TCRβ+ CD8β+	TCRβ+ CD4- CD8β-	TCRγδ+
Control	*Ripk1*$^{FL/FL}$	1.32 (±0.81)	0.84 (±0.22)	2.12 (±0.40)	5.75 (±0.98)
	Ripk1$^{\Delta CD4}$	3.13 (±0.38)	15.5 (±2.5)	1.09 (±0.71)	19.5 (±2.01)
Antibiotics (ABX)	*Ripk1*$^{FL/FL}$	0.58 (±0.12)	1.26 (±0.28)	1.67 (±0.22)	7.50 (±1.45)
	Ripk1$^{\Delta CD4}$	0.85 (±0.32)	2.36 (±0.87)	0.37 (±0.07)	18.9 (±5.34)
**p-value (significance)**					
Ripk1$^{FL/FL}$ (Control) vs. *Ripk1*$^{\Delta CD4}$ (Control)		0.0007 ***	<0.0001 ****	0.01 *	0.004 **
Ripk1$^{FL/FL}$ (ABX) vs. *Ripk1*$^{\Delta CD4}$ (ABX)		0.66 ns	0.22 ns	0.002 **	0.05 ns
Ripk1$^{FL/FL}$ (Control) vs. *Ripk1*$^{FL/FL}$ (ABX)		0.62 ns	0.29 ns	0.69 ns	0.53 ns
Ripk1$^{\Delta CD4}$ (Control) vs. *Ripk1*$^{\Delta CD4}$ (ABX)		0.003 **	0.0002 ***	0.24 ns	0.81 ns

Figure EV4. SI elongation in *Ripk1*$^{\Delta CD4}$ mice is a response to the intestinal microbiome, while villus atrophy and TCRγδ$^+$ IEL expansion are not.

(**A**) Serum IgA concentrations in aged *Ripk1*$^{\Delta CD4}$ mice ($n = 17$) and *Ripk1*$^{FL/FL}$ littermates ($n = 16$), measured by multiplex analysis (Meso Scale Discovery). Data are shown as mean ± SEM, with means represented by bars and each dot representing an individual mouse. (**B**) Representative images of the colon with caecum isolated from aged *Ripk1*$^{\Delta CD4}$ mice and *Ripk1*$^{FL/FL}$ littermates, with and without ABX treatment. (**C**) Ki-67 staining on the duodenum of aged *Ripk1*$^{\Delta CD4}$ mice and *Ripk1*$^{FL/FL}$ littermates, with and without ABX treatment. Images are representative of two independent repeats with ($n = 3$) *Ripk1*$^{\Delta CD4}$ mice (both ABX and control groups) or ($n = 4$) *Ripk1*$^{FL/FL}$ littermates (both ABX and control groups). (**D**) Kaplan-Meier survival analysis of *Ripk1*$^{\Delta CD4}$ mice ($n = 16$) and *Ripk1*$^{FL/FL}$ littermates ($n = 21$) without ABX control, and *Ripk1*$^{\Delta CD4}$ mice ($n = 13$) and *Ripk1*$^{FL/FL}$ mice ($n = 13$) on ABX. (**E**) Table corresponding to Fig. 6J, indicating the absolute numbers of the different IEL populations and resulting *p*-values of the statistical analysis (Fisher's LSD two-way ANOVA), with ($n = 4$) *Ripk1*$^{\Delta CD4}$ mice (control) ($n = 3$) *Ripk1*$^{\Delta CD4}$ mice (ABX) or ($n = 5$) *Ripk1*$^{FL/FL}$ littermates (both ABX and control groups). (**A**, **D**) Data are combined from two independent repeats, or (**B**, **C**, **E**) representative of two independent repeats. Statistical significance was calculated in Graphpad Prism by (**A**) Mann-Whitney test $p < 0.0001$, (**D**) Gehan-Wilcoxon test for survival, *Ripk1*$^{FL/FL}$ vs *Ripk1*$^{\Delta CD4}$ (Control): $p = 0.0067$, Control vs ABX (*Ripk1*$^{\Delta CD4}$): $p = 0.8301$, or (**E**) two-way ANOVA on Log_2-transformed data with *p*-values indicated in table. ns = non-significant, $^*p < 0.05$, $^{**}p < 0.01$, $^{***}p < 0.001$, $^{****}p < 0.0001$. Aged mice: >6 months old. IEL intraepithelial lymphocytes. Source data are available online for this figure.

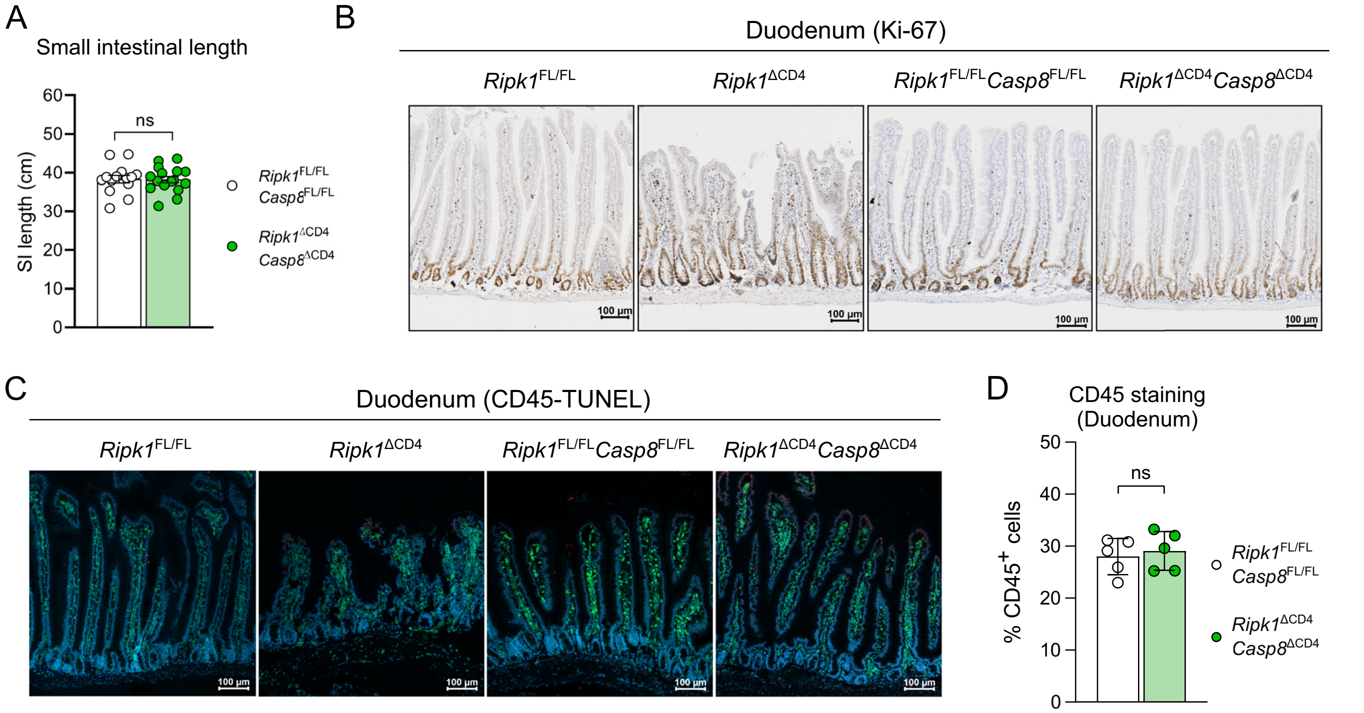

E

IELs (Fig. 7I)

Absolute number (x10⁵) (±SEM)	TCRβ+ CD4+	TCRβ+ CD8β+	TCRβ+ CD4- CD8β-	TCRγδ+
*Ripk1*FL/FL	0.60 (±0.11)	1.93 (±0.28)	3.68 (±0.35)	12.5 (±1.23)
*Ripk1*ΔCD4	1.59 (±0.23)	6.80 (±2.56)	0.85 (±0.07)	41.2 (±6.10)
*Ripk1*FL/FL*Casp8*FL/FL	0.37 (±0.05)	1.36 (±0.06)	4.40 (±1.53)	8.17 (±0.88)
*Ripk1*ΔCD4*Casp8*ΔCD4	0.64 (±0.09)	1.52 (±0.48)	2.35 (±1.31)	15.5 (±2.57)
p-value (significance)				
*Ripk1*FL/FL vs. *Ripk1*ΔCD4	0.002 **	0.0009 ***	<0.0001 ****	0.0005 ***
*Ripk1*FL/FL*Casp8*FL/FL vs. *Ripk1*ΔCD4*Casp8*ΔCD4	0.13 ns	0.93 ns	0.009 **	0.08 ns
*Ripk1*ΔCD4 vs. *Ripk1*ΔCD4*Casp8*ΔCD4	0.005 **	<0.0001 ****	0.05 ns	0.003 **

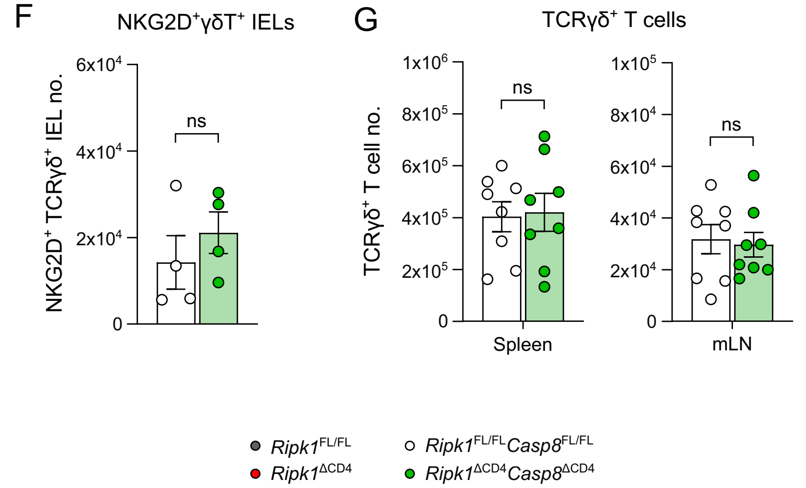

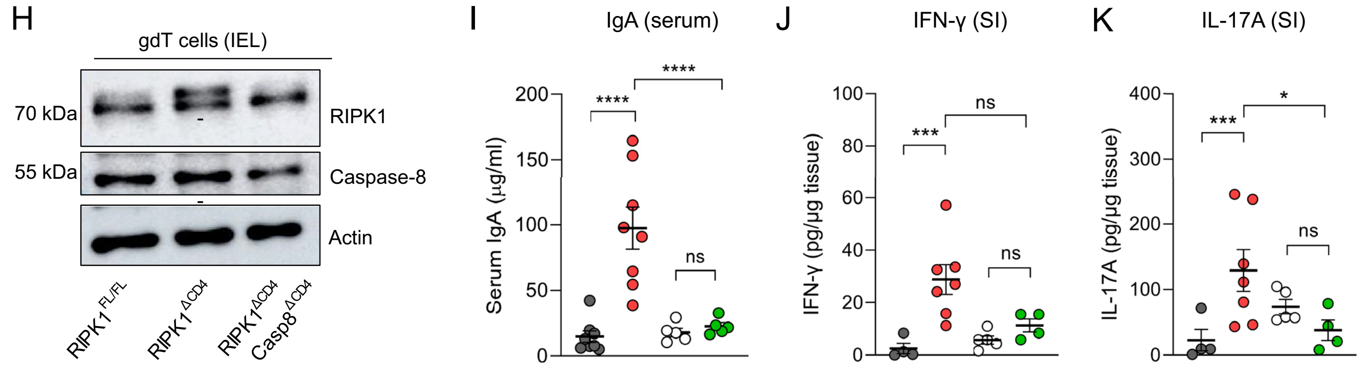

 © The Author(s)

Figure EV5. Villus atrophy is TNFR1-dependent, and T cell ablation of caspase-8 prevents the development of intestinal pathology.

(**A**) Quantification of the absolute small intestine (SI) length of *Ripk1*$^{\Delta CD4}$*Casp8*$^{\Delta CD4}$ mice ($n = 16$) and *Ripk1*$^{FL/FL}$*Casp8*$^{FL/FL}$ littermates ($n = 14$). (**B**) Ki-67 staining on duodenum sections of aged *Ripk1*$^{\Delta CD4}$*Casp8*$^{\Delta CD4}$ mice and *Ripk1*$^{FL/FL}$*Casp8*$^{FL/FL}$ littermates. (**C**) CD45-TUNEL staining on duodenum sections of aged *Ripk1*$^{\Delta CD4}$ and *Ripk1*$^{\Delta CD4}$*Casp8*$^{\Delta CD4}$ mice and their respective *Ripk1*$^{FL/FL}$ and *Ripk1*$^{FL/FL}$*Casp8*$^{FL/FL}$ littermates (CD45 = Green; TUNEL = Red). (**D**) Quantification of CD45 staining in the duodenum of aged *Ripk1*$^{\Delta CD4}$*Casp8*$^{\Delta CD4}$ mice and *Ripk1*$^{FL/FL}$*Casp8*$^{FL/FL}$ littermates ($n = 5$), represented as the percentage of CD45-positive cells within the total number of cells (determined by DAPI staining). (**E**) Table corresponding to Fig. 7I, indicating the absolute numbers of the different IEL populations and their resulting *p*-values of the statistical analysis (Fisher's LSD two-way ANOVA). (**F**) Absolute numbers of NKG2D^{+} TCRγδ^{+} IELs in the SI epithelial layer of young *Ripk1*$^{\Delta CD4}$*Casp8*$^{\Delta CD4}$ mice and *Ripk1*$^{FL/FL}$*Casp8*$^{FL/FL}$ littermates ($n = 4$), measured by flow cytometry. (**G**) Absolute numbers of TCRγδ^{+} T cells in the spleen (left) and mLN (right) of young *Ripk1*$^{\Delta CD4}$*Casp8*$^{\Delta CD4}$ mice and *Ripk1*$^{FL/FL}$*Casp8*$^{FL/FL}$ littermates ($n = 8$), measured by flow cytometry. (**H**) Western blot on FACS-sorted TCRγδ^{+} IELs, showing RIPK1, Caspase-8, and Actin in *Ripk1*$^{FL/FL}$, *Ripk1*$^{\Delta CD4}$ and *Ripk1*$^{\Delta CD4}$*Casp8*$^{\Delta CD4}$ mice. (**I**) Serum IgA concentrations in young *Ripk1*$^{\Delta CD4}$ mice ($n = 8$) and *Ripk1*$^{FL/FL}$ littermates ($n = 8$), and aged *Ripk1*$^{\Delta CD4}$*Casp8*$^{\Delta CD4}$ mice ($n = 5$) and *Ripk1*$^{FL/FL}$*Casp8*$^{FL/FL}$ littermates ($n = 5$), measured by multiplex analysis (Meso Scale Discovery). (**J**, **K**) Tissue concentrations of (**J**) IFN-γ, and (K) IL-17A in the SI of young *Ripk1*$^{\Delta CD4}$ ($n = 7$) and *Ripk1*$^{\Delta CD4}$*Casp8*$^{\Delta CD4}$ ($n = 5$) mice and their respective *Ripk1*$^{FL/FL}$ ($n = 4$) and *Ripk1*$^{FL/FL}$*Casp8*$^{FL/FL}$ ($n = 5$) littermates, measured by multiplex analysis (Meso Scale Discovery). Data are representative of two (**E**) or three (**A**) independent repeats, or (**G**) are combined from two independent repeats. Data are shown as mean ± SEM, with means being represented by bars or horizontal lines and each dot representing an individual mouse. Statistical significance was calculated in Graphpad Prism by unpaired T-test with Welch's correction (**A**, **D**, **F**, **G**), or Fisher's LSD two-way ANOVA on Log_2-transformed data (**E**) with p-values indicated in table (**I**) *Ripk1*$^{FL/FL}$ vs *Ripk1*$^{\Delta CD4}$: $p < 0.0001$, *Ripk1*$^{\Delta CD4}$ vs *Ripk1*$^{\Delta CD4}$*Casp8*$^{\Delta CD4}$: $p < 0.0001$, *Ripk1*$^{FL/FL}$*Casp8*$^{FL/FL}$ vs *Ripk1*$^{\Delta CD4}$*Casp8*$^{\Delta CD4}$: $p = 0.7831$; (**J**) *Ripk1*$^{FL/FL}$ vs *Ripk1*$^{\Delta CD4}$: $p = 0.0002$, *Ripk1*$^{\Delta CD4}$ vs *Ripk1*$^{\Delta CD4}$*Casp8*$^{\Delta CD4}$: $p = 0.1071$, *Ripk1*$^{FL/FL}$*Casp8*$^{FL/FL}$ vs *Ripk1*$^{\Delta CD4}$*Casp8*$^{\Delta CD4}$: $p = 0.1931$; (**K**) *Ripk1*$^{FL/FL}$ vs *Ripk1*$^{\Delta CD4}$: $p = 0.0009$, *Ripk1*$^{\Delta CD4}$ vs *Ripk1*$^{\Delta CD4}$*Casp8*$^{\Delta CD4}$: $p = 0.0401$, *Ripk1*$^{FL/FL}$*Casp8*$^{FL/FL}$ vs *Ripk1*$^{\Delta CD4}$*Casp8*$^{\Delta CD4}$: $p = 0.1621$. ns = non-significant, *$p < 0.05$, **$p < 0.01$, ***$p < 0.001$, ****$p < 0.0001$. Young mice: 8–12 weeks old, aged mice: >6 months old. IEL intraepithelial lymphocytes. Source data are available online for this figure.
